# Selective oxidation of silanes into silanols with water using [MnBr(CO)_5_] as a precatalyst†

**DOI:** 10.1039/d2sc05959b

**Published:** 2022-12-01

**Authors:** Emanuele Antico, Markus Leutzsch, Niklas Wessel, Thomas Weyhermüller, Christophe Werlé, Walter Leitner

**Affiliations:** Max Planck Institute for Chemical Energy Conversion Stiftstr. 34–36 45470 Mülheim an der Ruhr Germany christophe.werle@cec.mpg.de walter.leitner@cec.mpg.de; Institut für Technische und Makromolekulare Chemie (ITMC), RWTH Aachen University Worringer Weg 2 52074 Aachen Germany; Max-Planck-Institut für Kohlenforschung Kaiser-Wilhelm-Platz 1 45470 Mülheim an der Ruhr Germany; Ruhr University Bochum Universitätsstr. 150 44801 Bochum Germany

## Abstract

The development of earth-abundant catalysts for the selective conversion of silanes to silanols with water as an oxidant generating valuable hydrogen as the only by-product continues to be a challenge. Here, we demonstrate that [MnBr(CO)_5_] is a highly active precatalyst for this reaction, operating under neutral conditions and avoiding the undesired formation of siloxanes. As a result, a broad substrate scope, including primary and secondary silanes, could be converted to the desired products. The turnover performances of the catalyst were also examined, yielding a maximum TOF of 4088 h^−1^. New light was shed on the debated mechanism of the interaction between [MnBr(CO)_5_] and Si–H bonds based on the reaction kinetics (including KIEs of PhMe_2_SiD and D_2_O) and spectroscopic techniques (FT-IR, GC-TCD, ^1^H-, ^29^Si-, and ^13^C-NMR). The initial activation of [MnBr(CO)_5_] was found to result from the formation of a manganese(i) hydride species and R_3_SiBr, and the experimental data are most consistent with a catalytic cycle comprising a cationic tricarbonyl Mn(i) unit as the active framework.

## Abstract 2

This study presents the use of MnBr(CO)_5_ for the selective conversion of silanes to silanols with water as an oxidant generating valuable hydrogen as the only by-product.

## Graphical abstract

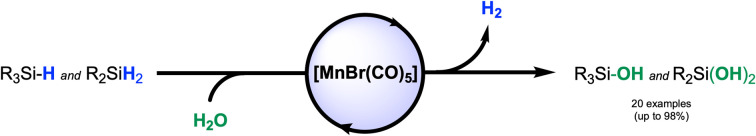

## Introduction

**Scheme 1:**
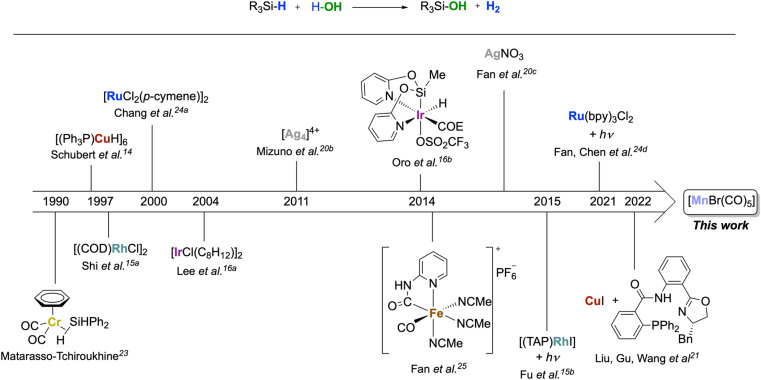
Homogeneous transition metal complexes as catalysts for the oxidation of silanes to silanol with H_2_O.

The oxidation of silanes to silanols is an important reaction due to the wide range of fields in which these products can be applied, including material sciences^1^ and medicinal chemistry,^2^ as well as in synthetic chemistry as directing groups,^3^ organocatalysts^4^ or cross-coupling partners.^5^ Silanols are traditionally prepared by hydrolysis of chloro- or alkoxysilanes under strict buffering conditions and with stoichiometric amounts of base.^6^ Alternatively, they can be obtained by oxidation of organosilanes using stoichiometric oxidants such as KMnO_4_,^7^ OsO_4_,^8^ dioxiranes,^9^ or O_3_.^10^ There is, therefore, a strong interest in developing alternative protocols based on the principles of *Green Chemistry*.^11^ In this respect, H_2_O as an oxidant^12^ is an ideal candidate for this reaction as the reaction results in the formation of H_2_ as a valuable by-product.^6*c*,12,13^ However, the formation of siloxane side products from hydrolysis is a frequently encountered limitation.^13,14^ To make the Si–H bond sufficiently reactive, noble metals are typically required. Examples of homogeneously and heterogeneously catalyzed reactions are mostly based on metals of group 9–11, such as Rh,^15^ Ir,^16^ Pd,^17^ Pt,^18^ Au,^19^ or Ag^20^ (Scheme 1). Notably, Schubert^14^ reported a copper cluster [(Ph_3_P)CuH]_6_ converting a narrow scope of tertiary silanes to their respective silanols with, however, some selectivity issues. Another example of a copper catalyst was recently used at 10% loading for enantioselective hydroxylation of secondary silanes.^21^ The use of metals from groups 6 and 7 as homogenous catalysts for this reaction is comparatively rare.^22^ In 1990, Matarasso-Tchiroukhine studied a chromium(0) carbonyl complex where σ-coordination of Ph_2_SiH_2_ to the metal was inferred to activate the silane to become reactive with water and other nucleophiles.^23^ Moving to group 8, a number of Ru systems have been developed for silane oxidation.^24^ The 3d congener iron also shows promises, as demonstrated by Fan's report on a highly active Fe(ii) carbonyl complex optimized for the generation of H_2_ using excess water.^25^

In order to move away from noble metals to more environmentally friendly and cheaper metals, a possible approach is to search for chemical analogies within the periodic table *via* the so-called “diagonal relationship.” It compares a metal ion in the oxidation state *n*^+^ with a congener in the period above and the group to the left in the oxidation state (*n* − 1)^+^. While this analogy is mainly applied to main group metals, it is also reflected in a number of cases for transition metal catalysis.^26^ From ruthenium(ii), this leads to manganese(i) as a possible target, and the concept has proven particularly fruitful for hydrogen transfer processes.^27*a*^ Despite its low cost, non-toxicity, and widespread availability, manganese is under-represented in the field of silane oxidation, however. To the best of our knowledge, only one example has been reported to date using a manganese-based catalyst for silane oxidation applying the much stronger oxidant H_2_O_2_.^13^ On the other hand, manganese carbonyl complexes have shown good performance in hydrosilylation reactions^28^ and the formation of Si–O bonds from hydrosilanes,^29^ and different mechanisms have been proposed for the Si–H bond activation at Mn-complexes. Therefore, we decided to explore the activity of the simple and commercially available [MnBr(CO)_5_] for the oxidation of silanes with water as the oxidant (Scheme 1).

## Results and discussion

**Scheme 2:**
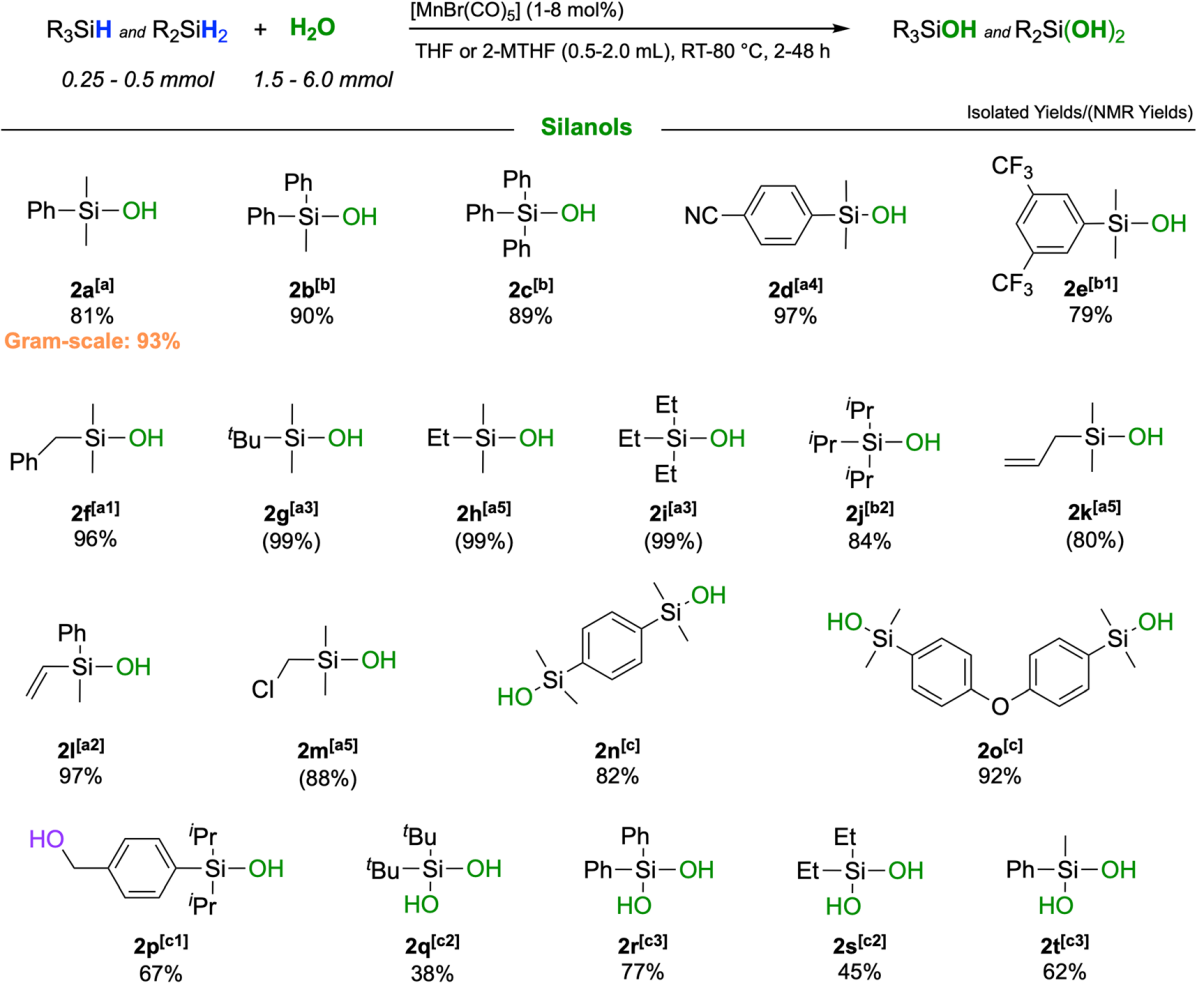
Scope of the reaction: isolated yields are given below each respective silanol 2a–t; NMR yields (in parentheses) were determined relative to mesitylene as an internal standard; the isolated gram-scale synthesis yield is indicated in orange. [a]: [MnBr(CO)_5_] = 1 mol%, H_2_O (1.5 mmol), *T* = 50 °C, silane (0.5 mmol), solvent = THF (0.5 mL), time = 1 h; [a1]: time = 2 h; [a2]: time = 4 h; [a3]: time = 24 h; [a4]: time = 4 h, *T* = 60 °C; [a5]: time = 24 h, *T* = RT; [b]: see [a] with deviations: solvent = 2-MTHF and *T* = 80 °C; [b1]: see [b] with deviations: [MnBr(CO)_5_] = 2 mol% [b2]: see [b] with deviations: [MnBr(CO)_5_] = 2 mol%, time = 5 h; [c]: [MnBr(CO)_5_] = 2 mol%, H_2_O (6 mmol), *T* = 50 °C, silane (0.25 mmol), solvent = THF (2.0 mL), time = 4 h; [c1]: see [c] with deviations: time = 48 h; [c2]: see [c1] with deviations: solvent = THF (1.0 mL), [MnBr(CO)_5_] = 4 mol%; [c3]: see [c2] with deviations: *T* = RT and [MnBr(CO)_5_] = 8 mol%.

Dimethylphenylsilane, one of the most sensitive substrates towards the formation of siloxane dimers^14,24*a*^ was chosen as a model substrate to test for activity and to identify suitable reaction conditions (Table S1†). After some optimization, the reaction of PhMe_2_SiH (1) in THF at 50 °C with H_2_O (5 equiv.) in the presence of [MnBr(CO)_5_] as a precatalyst afforded a quantitative yield of PhMe_2_SiOH (2a) already at a loading of only 1 mol% of [MnBr(CO)_5_] after 1 hour (Table 1, entry 2). While the reaction worked in a wide range of solvents, the best results were obtained in solvents of medium coordination strengths such as ethyl acetate, THF, and 2-MTHF. Interestingly, no hydrosilylated side-products were detected when employing acetone or EtOAc as reaction solvents, although manganese carbonyl complexes are known to be active catalysts in the hydrosilylation of carbonyl and carboxyl groups.^30^ The reaction proceeded to completion in THF even with only stoichiometric amounts of water, but a 3 : 1 ratio of H_2_O to silane was found to give optimum selectivity. The parameters of entry 11 in Table 1 were adopted as standard conditions and applied directly or with some modification to a scope of different silanes shown in Scheme 2.

**Table 1:** Optimization of solvents and H_2_O/substrate ratio for the oxidation of dimethylphenylsilane with H_2_O

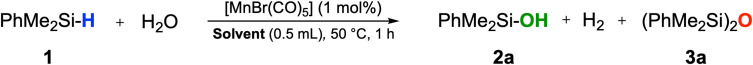				
Entry	Solvent	H_2_O equiv.	NMR yield (2a)	Conversion
1	Acetone	5	89	>99
2	THF	5	>99	>99
3	Heptane	5	40	>99
4	Toluene	5	55	>99
5	Chloroform	5	74	>99
6	Acetonitrile	5	27	>99
7	2-MTHF	5	99	>99
8	DMSO	5	62	>99
9	EtOAc	5	96	>99
10	THF	4	>99	>99
11	**THF**	**3**	**>99**	**>99**
12	THF	2	90	>99
13	THF	1	78	>99

The catalytic oxidation gave very high to excellent yields across a broad range of different functionalities. Replacing the methyl groups in 2a with phenyl rings (2b, 2c) as well as the presence of electron-withdrawing substituents such as cyano-(2d) or trifluoromethyl-(2e) was fully tolerated affording yields up to 97%. Also, silanes containing purely aliphatic substituents were converted to their respective silanols with excellent outcomes (2f–j). Notably, unsaturated groups like allyl- or vinyl-groups remained fully intact, and high yields of the respective silanols 2k and 2l were obtained. Difunctional silanes, more prone to forming siloxanes as oligomers, yielded silanols 2m–p in excellent yields when a larger excess of H_2_O and higher dilution were applied. Notably, the corresponding products would be difficult to obtain by employing traditional synthetic procedures. It was also possible to react secondary silanes to the respective silanediols, such as the bulky di-*tert*-butylsilanediol (2q) or diphenylsilanediol (2r), diethyl-(2s) and methylphenylsilanediol (2t), albeit the yields were slightly lower for these very challenging substrates. Finally, to probe the practicability of the protocol, a gram-scale reaction was performed with PhMe_2_SiH as a substrate affording pure PhMe_2_SiOH in 93% isolated yield (Section S6†). The turnover performances of the catalyst were examined, yielding a maximum TOF of 4088 h^−1^.

Experimental studies were then performed to shed light on the reaction mechanism. First, we examined the activity of other Mn(i) carbonyl complexes in the targeted reaction (Table S4†) and found them to be inert for the conversion of PhMe_2_SiH to any product. Furthermore, manganese salts and complexes in other oxidation states than +I, such as MnBr_2_, MnCl_2_, Mn(OAc)_3,_ and [Mn_2_(CO)_10_], also showed no conversion of the starting material (Table S5†). Similar observations were made previously in reduction reactions using silanes, indicating a particular ability of [MnBr(CO)_5_] to activate Si–H bonds.^28^ A stoichiometric reaction between [MnBr(CO)_5_] and PhMe_2_SiH led to an evolution of CO within seconds after the dissolution of both reagents in THF-*d*_8_ (Confirmed *via* GC-TCD, Section S9.3†). The experiment was followed *via*^1^H- and ^29^Si-NMR spectroscopy: after 15 minutes, two new singlets at −7.85 ppm and 0.81 ppm were detected in the ^1^H-NMR, corresponding to a hydride in a complex of general formula [MnH(CO)_*n*_(L)_*m*_] (L = solvent) and the methyl-groups of PhMe_2_SiBr, respectively (confirmed by ^29^Si-NMR, Fig. S4†). A catalytic reaction between PhMe_2_SiH and H_2_O at 2 mol% catalyst loading was followed *via* FT-IR. This demonstrated full conversion of [MnBr(CO)_5_] within 1 hour at 35 °C forming primarily species comprising a tricarbonyl unit “Mn(CO_3_)” (Section S10.1†). Gas-phase TCD analysis of the headspace of the reaction further supported this assignment showing an average loss of 2 CO per initially charged [MnBr(CO)_5_].

Taken together, the data indicate that the catalyst activation proceeds *via* loss of CO and formal “hydride-bromide” exchange to give tricarbonyl manganese hydride species of general formula [MnH(CO)_3_(L)_2_] (L = solvent or water). This differs from earlier literature reports using the dimeric Mn(i) precursor [{MnBr(CO_4_)}_2_] as a precatalyst for the alcoholysis of PhMe_2_SiH and suggesting an oxidative addition/elimination pathway to form [Mn(SiMe_2_Ph)(CO)_4_] as the active species.^29*a*^

**Fig. 1:**
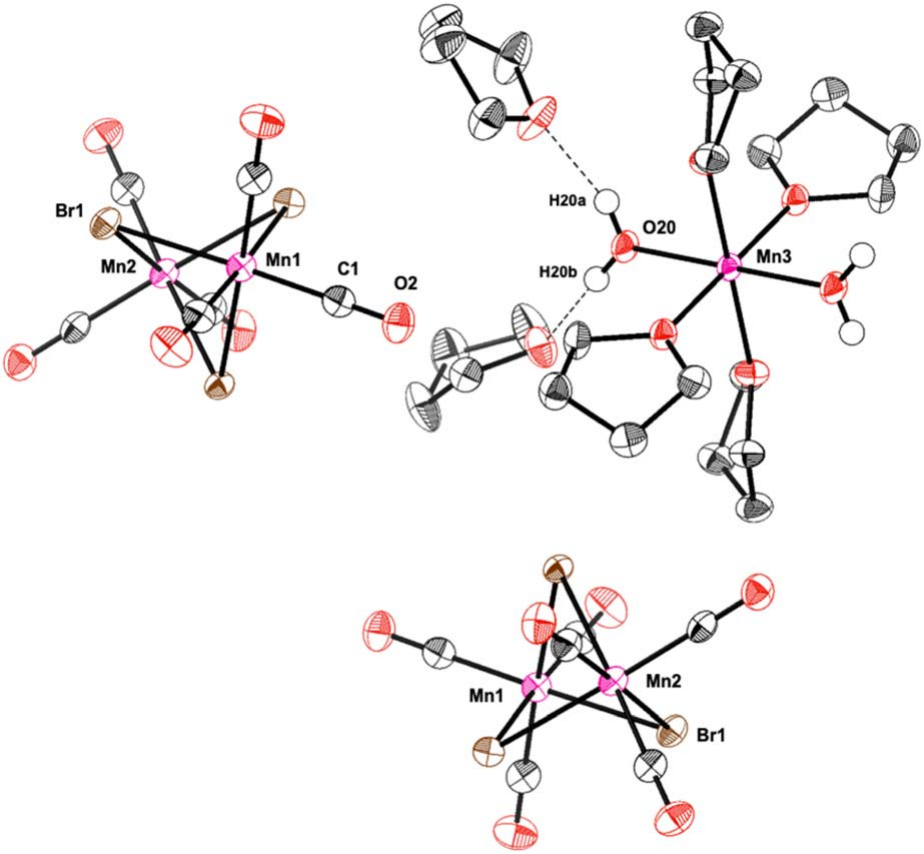
Molecular structure of Mn-5. The molecular structure is shown with thermal ellipsoids drawn at the 40% probability level. For clarity reasons, most of the hydrogen atoms were omitted.

Further evidence on the Mn-species formed during the catalytic reaction was obtained from analysis of the solid material obtained after water extraction of the reaction mixture and subsequent evaporation of all volatiles from the aqueous phase at the end of the reaction. The obtained red solid was found to retain some of the initial catalytic activity upon reuse in a second reaction with fresh solvent and substrate (61% conversion after 1 h). X-Ray diffraction of crystals obtained from a portion of the solid upon washing and recrystallization revealed the presence of Mn-5: a cationic mononuclear *trans*-[Mn(ii)(THF)_4_(OH_2_)_2_]^2+^ and two dinuclear anions [(CO)_6_Mn_2_(μ-Br)_3_]^−^ as counterions (Fig. 1). The oxidation of Mn(i) to Mn(ii) in the cation most likely results from adventitious oxygen during the crystallization attempts of the small amounts of solid. The presence of the Mn(i) tricarbonyl units in the anionic dimer is again consistent with the proposed key role of this fragment for the catalytic activity in the reaction.

**Fig. 2:**
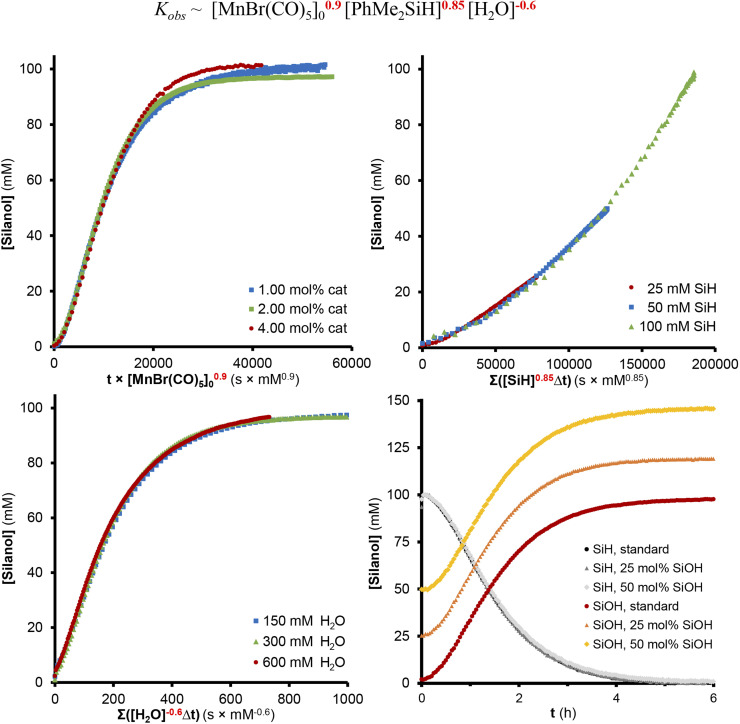
Rate equation and kinetic plots for the reaction obtained using VTNA analysis of the NMR data plotted with the coefficients affording the best overlap: the formation of PhMe_2_SiOH shows 0.9th order dependence in [MnBr(CO)_5_] (top-left), 0.85th in [PhMe_2_SiH] (top-right), −0.6th in [H_2_O] (bottom-left) and does not show product inhibition (bottom-right).

The rate of the reaction was studied in THF-*d*_8_ at 35 °C by variation of the relevant concentration of silane, H_2_O, and precatalyst (Fig. 2). Variable Time Normalization Analysis (VTNA)^31^ of the concentration profiles obtained by ^1^H-NMR spectroscopy was utilized to determine the overall reaction orders of the reaction components. The best curve overlaps showed a positive reaction order of 0.9 in [MnBr(CO)_5_] and 0.85 in PhMe_2_SiH close to unity, and a significant inhibiting effect with a negative reaction order of −0.6 in H_2_O while no inhibition was observed for the product PhMe_2_SiOH. Notably, neither PhMe_2_SiD nor D_2_O showed a measurable kinetic isotope effect (Sections S8.5 and S8.6†), suggesting that the cleavage of Si–H or O–H bonds are not involved in the rate-determining step of the reaction.

**Scheme 3:**
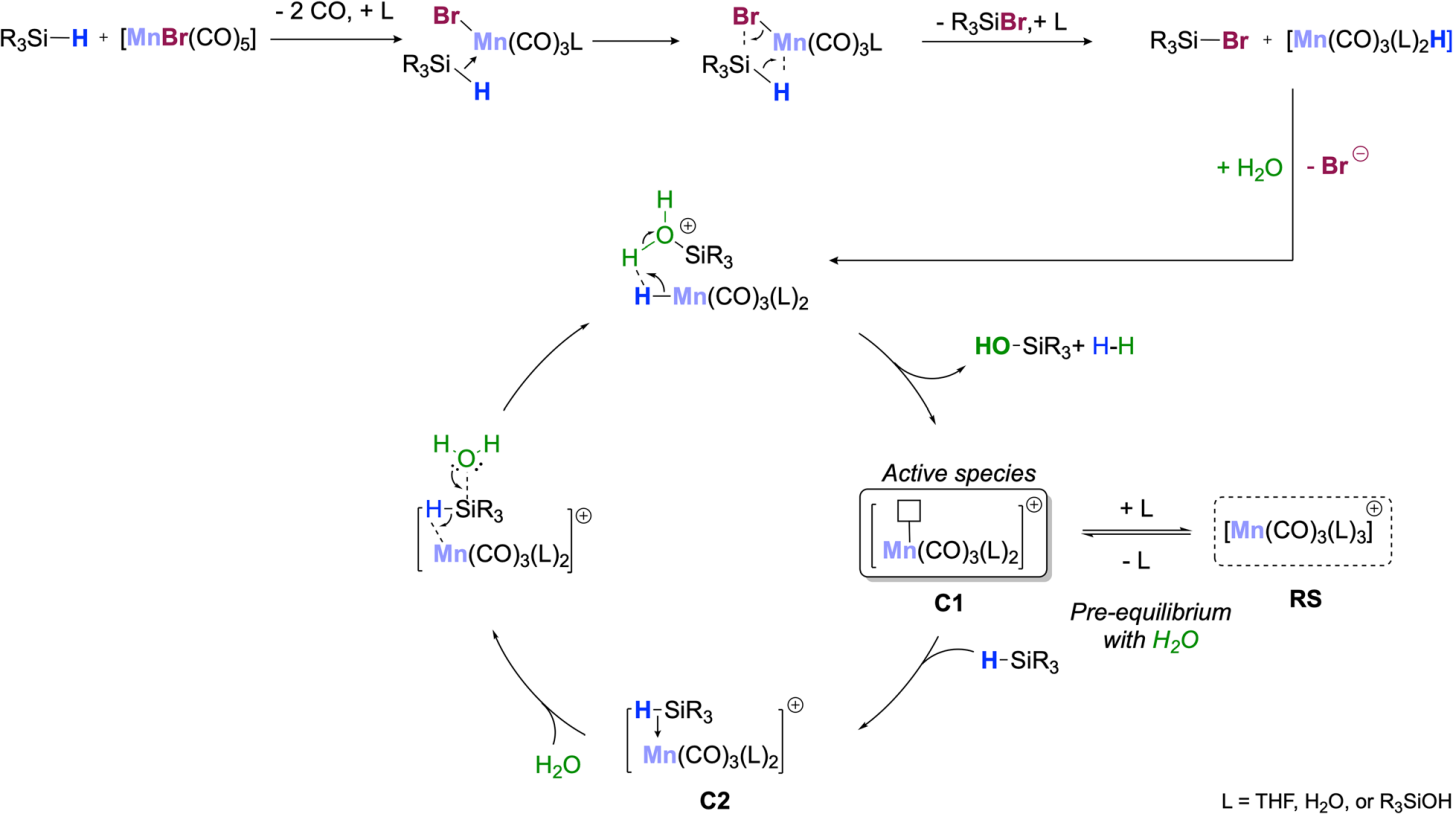
Reaction mechanism consistent with the experimental data.

Scheme 3 shows a proposed reaction mechanism for the catalytic oxidation of silanes to silanols using [MnBr(CO)_5_] as a precatalyst that is most consistent with the described experimental data. In the initial activation step, the silane reacts with the precatalyst to form the corresponding bromosilane R_3_SiBr and a hydride complex [MnH(CO)_5−*n*_(L)_*n*_] with *n* = 2 as the most likely composition. Hydrolysis of the Si–Br unit leads to the formation of the silanol, and protonation of the Mn–H bond liberates H_2_ as the second desired product. The resulting unsaturated complex of the type [Mn(CO)_3_(L)_2_]^+^ (C1, Scheme 3; L = solvent, H_2_O, or R_3_SiOH) can be viewed as the active species carrying the catalytic cycle. Coordination of additional water as L converts C1 into a 6-coordinated off-cycle resting state RS, explaining the negative reaction order in H_2_O. Alternatively, re-coordination of Br^−^ would also seem conceivable. This is less likely due to the large excess of water under turnover conditions but would explain the formation of the dimeric Mn(i)-tricarbonyl anion isolated after the reaction. In competition with the other neutral ligands L, σ-coordination of R_3_SiH generates the adduct C2 as the next productive species activating the silane for the nucleophilic attack of H_2_O. In line with this proposal, cationic and monomeric Mn(i) complexes such as [Mn(CO)_3_(L)_2_]^+^ are known to perform heterolytic cleavage of Si–H bonds already at RT,^29*b*,32^ and constitute, therefore, plausible candidates as active species in agreement with the absence of H/D kinetic isotope effects. The slight deviations in the reaction orders for [MnBr(CO)_5_] and PhMe_2_SiH from unity may indicate that the silane could also be involved in slow degradation of the catalyst to inactive species (Section S10.4†).

## Conclusions and outlook

We have shown that the commercial, simple, and bench-stable manganese complex [MnBr(CO)_5_] is an active precatalyst for the selective oxidation of silanes to silanols using H_2_O as the oxidant with stoichiometric hydrogen evolution under neutral conditions. The newly developed protocol allowed the preparation of primary and secondary silanols, including also di-silanols, some of which would be incompatible with traditional synthetic procedures. The reaction showed good to excellent selectivity for a broad range of functionalized products and was readily performed on gram-scale. The reaction mechanism has been thoroughly investigated, being most consistent with a catalytic cycle mediated by a Mn(i) tricarbonyl unit involving cationic complexes of general formula [Mn(CO)_3_(L)_3_]^+^ (L = solvent, H_2_O, or R_*n*_Si(OH)_4−*n*_) as active species.

The data presented here may help to rationalize the activity of [MnBr(CO)_5_] for catalytic reactions involving the activation and transfer of Si–H groups through further experimental and theoretical work. It will be interesting to see whether similar elementary steps can operate in other manganese catalysis involving E–H cleavage (*e.g.*, hydroboration or (transfer-)hydrogenation). While ligand cooperativity^33^ is a very important concept in manganese catalysis, the remarkable activity of ligand-free Mn-carbonyl complexes for σ-bond activation associated with the [Mn(CO)_3_]^+^ framework may offer alternative design criteria for future catalyst systems.

## Data availability

The data that support the findings of this study are available in the ESI.†

## Author contributions

E. A. performed the experiments, conducted the analytical characterization, and prepared the original draft. M. L. designed and performed the kinetic experiments and their data analysis for the mechanistic studies. N. W. provided assistance in the acquisition and processing of the FT-IR experiments. T. W. collected and analyzed the single-crystal X-ray diffraction data. C. W. and W. L. formulated, supervised, and directed the overall project. C. W. and W. L. revised the manuscript with the contribution of the other authors. All authors have approved the final version of the manuscript.

## Conflicts of interest

There are no conflicts to declare.

## Supplementary Material

SC-014-D2SC05959B-s001

SC-014-D2SC05959B-s002
