# Supplementary material for: Selective oxidation of silanes into silanols with water using [MnBr(CO)5] as a precatalyst
Source: Chem Sci. 2022 Dec 1;14(1):54–60. doi: 10.1039/d2sc05959b (PMC9769106; doi:10.1039/d2sc05959b)
Supplement: SC-014-D2SC05959B-s001 [file SC-014-D2SC05959B-s001.pdf]

Electronic Supplementary Material (ESI) for Chemical Science.
This journal is © The Royal Society of Chemistry 2022

Supporting Information

Selective Oxidation of Silanes into Silanols with Water using [MnBr(CO)$_5$] as a Precatalyst

Emanuele Antico,[1,2] Markus Leutzsch,[3] Niklas Wessel,[1,2] Thomas Weyhermüller,[1] Christophe Werlé[1,4]* and Walter Leitner[1,2]*

1 Max Planck Institute for Chemical Energy Conversion, Stiftstr. 34 – 36, 45470 Mülheim an der Ruhr, Germany.
2 Institut für Technische und Makromolekulare Chemie (ITMC), RWTH Aachen University, Worringer Weg 2, 52074 Aachen, Germany.
3 Max-Planck-Institut für Kohlenforschung, Kaiser-Wilhelm-Platz 1, 45470 Mülheim an der Ruhr, Germany.
4 Ruhr University Bochum, Universitätsstr. 150, 44801 Bochum, Germany.

* Emails: christophe.werle@cec.mpg.de, walter.leitner@cec.mpg.de

Table of Contents

1. **GENERAL CONSIDERATIONS** **S4**

2. **REACTION OPTIMIZATION** **S5**

   2.1. General Method and screening of catalyst loading S5
   2.2. Solvent screening S6
   2.3. H_2O Stoichiometry Screening S7
   2.4. Screening of other Manganese carbonyl complexes S8
   2.5. Screening of other Mn(X) complexes S9

3. **REACTION SCOPE** **S10**

   3.1. Procedure (a) S11
   3.2. Procedure (b) S11
   3.3. Procedure (c) S12
   3.4. Procedure (d) S12
   3.5. NMR yields S13

4. **SYNTHESIS OF NON-COMMERCIAL SUBSTRATES** **S13**

   4.1. Synthesis of $PhMe_2SiD$ S13
   4.2. Synthesis of ^{13}C-enriched $[MnBr(CO)_5]$ S14

5. **CHARACTERIZATION DATA** **S17**

6. **PREPARATIVE SCALE REACTION** **S22**

7. **STOICHIOMETRIC NMR EXPERIMENTS** **S23**

   7.1. Stoichiometric reaction between $PhMe_2SiH$ and $[MnBr(CO)_5]$ S23
   7.2. Comparison with the reaction with bromine S26

8. **KINETIC STUDIES** **S28**

   8.1. General procedure S28
   8.2. Order in $[MnBr(CO)_5]$ S30
   8.3. Order in $PhMe_2SiH$ S33
   8.4. Order in H_2O S36
   8.5. Kinetic Isotope Effect of $PhMe_2SiD$ S39
   8.6. Kinetic Isotope Effect of D_2O S39
   8.7. Product inhibition S40
   8.8. Experiment with enriched $[MnBr(^{13}CO)_5]$ S41

9. GAS-PHASE ANALYSIS STUDIES S43

9.1. STUDY ON [$MnBr(CO)_5$] S43
9.2. STUDY ON **MN-4** S44
9.3. STUDY ON THE ACTIVATION OF [$MnBr(CO)_5$], THE PRECATALYST S45

10. MECHANISTIC STUDIES S46

10.1. GENERAL PROCEDURE FOR THE IR EXPERIMENTATION S46
10.2. FT-IR SPECTRA OF RELEVANT SUBSTANCES IN THF S47
10.3. KEY SPECTRA AND SIGNIFICANT ABSORBANCE-TIME PLOTS S48
10.4. CRYSTALLIZATION OF **MN-5** S49
10.5. CATALYTIC ACTIVITY OF THE SOLID OBTAINED IN SECTION 10.4 ...... S50

11. TON AND TOF STUDIES S51

12. NMR SPECTRA S52

12.1. NMR YIELDS S52
12.2. NMR SPECTRA OF ISOLATED SILANOLS S61
12.3. FAILED SUBSTRATES S84
12.4. GRAM SCALE REACTION S87
12.5. $PhMe_2SiD$ S89

13. X-RAY CRYSTALLOGRAPHY AND REFINEMENT OF STRUCTURES S92

13.1. MOLECULAR STRUCTURE OF COMPLEX **MN-5** (CCDC 2190208) S94
13.2. MOLECULAR STRUCTURE OF SILANOL **2P** (CCDC 2190209) S95
13.3. MOLECULAR STRUCTURE OF SILANOL **2O** (CCDC 2190210) S96

14. REFERENCES S97

1. General Considerations

Unless stated otherwise, all air-sensitive experiments were conducted under an argon atmosphere using standard Schlenk techniques or an MBraun inert-gas glovebox. Solvents for air- and moisture-sensitive experiments were purified through a MBraun-SPS-7 system or dried over activated molecular sieves (3 or 4Å) and degassed according to standard laboratory procedure. Starting materials were purchased from Sigma Aldrich, Abcr, Alfa Aesar, or TCI Europe and used as received. [$MnBr(CO)_5$] has been used as received from Sigma Aldrich or Alfa Aesar. NMR spectra were recorded on a Bruker AVANCE NEO 400 MHz, a Bruker AVANCE III HD 500 MHz NMR spectrometer with a Bruker Prodigy probe, or Bruker AVANCE NEO 600 MHz NMR spectrometer with a BBO cryoprobe. The reaction kinetics were studied on a Bruker AVANCE III HD 400 MHz NMR spectrometer equipped with a BBO probe. The chemical shifts (δ) are given in ppm (parts per million) relative to TMS and were assigned taking as a reference the residual solvent signals ($CDCl_3$: δ_H = 7.26 ppm, δ_C = 77.16 ppm; DMSO-d_6: δ_H = 2.50 ppm, δ_C = 39.52 ppm; THF-d_8: δ_H = 3.58 ppm, δ_C = 25.31 ppm). ^{13}C spectra were generally acquired with broadband proton decoupling. ^{29}Si-NMR spectra were recorded using a DEPT pulse sequence, and the chemical shifts are reported in ppm with respect to TMS (δ_{Si} = 0 ppm). The peak patterns are indicated as follows: s = singlet; d = doublet; t = triplet; q = quartet; h = sextet: m = multiplet; br = broad. Gas-phase analyses have been performed on a Shimadzu GC Nexis 2030 via manual injection (100 µL) equipped with a Restek Q-Bond column (Length: 30m; inner Diameter: 0.32 mm; film thickness: 10 µm) and a TCD detector using $He_{(g)}$ as a carrier gas. HR-MS have been recorded via an “LTQ-FT-Ultra” provided by Thermo Scientific. The flow FT-IR was a Bruker Vertex 70v with a Harrick Scientific high-pressure demountable liquid cell equipped with diamond windows with a path length of 100 µm. A 1/16’’ tubing connected the IR device to a Fischer-Porter tube. The sample was continuously pumped through the loop to the FT-IR spectrometer during sample measurement with a flow rate of 3 mL/min by a WADose Lite HP HPLC pump. The blue light “Evoluchem^{TM}” (450 nm) was purchased from Hepatochem and used in combination with the PhotoRedOx Box TC.

2. Reaction Optimization

2.1. General Method and screening of catalyst loading

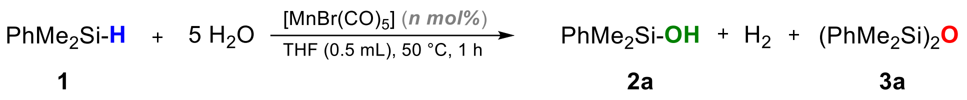

[Mn(CO)$_5$Br] (see Table S1), mesitylene (20 µL, 0.14 mmol), and dimethylphenylsilane (68.1 mg, 0.5 mmol) were weighed in a 4 mL vial equipped with a screw cap and a magnetic stirrer inside the glovebox. The reaction vessel was taken outside the glovebox, placed in a Schlenk tube previously evacuated, and filled with argon three times. H_2O (45 µL, 2.5 mmol) and THF (0.5 mL) were added to the vial under argon flush. The reaction mixture was heated at 50 °C for 1 hour. The reaction vessel was allowed to cool down to RT. An NMR tube was filled with the crude mixture (ca 0.05 mL), DMSO-d_6 (0.5 mL), and ^{1}H NMR spectroscopy was conducted to determine the conversion and yield of the reaction. The Si-OH integral (5.88 ppm, 1H) was set at 1.00 and compared with the characteristic peak value of mesitylene (6.76 ppm, 3H).

Table S1 – Screening of different [MnBr(CO)$_5$] loadings.

**[MnBr(CO)$_5$]**	**Yield**	**Conversion**
5 mol%	95	100
2 mol%	99	100
**1 mol%**	**>99**	**100**

2.2. Solvent screening

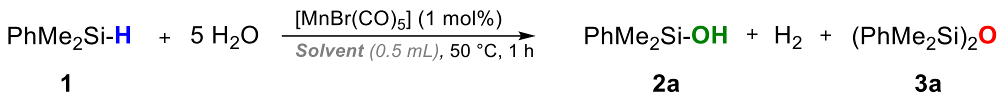

[Mn(CO)$_5$Br] (1.3 mg, 0.005 mmol), mesitylene (20 µL, 0.14 mmol), and dimethylphenylsilane (68.1 mg, 0.5 mmol) were weighed in a 4 mL vial equipped with a screw cap and a magnetic stirrer inside the glovebox.. The reaction vessel was taken outside the glovebox, placed in a Schlenk tube previously evacuated, and filled with argon three times. H_2O (45 µL, 2.5 mmol) and the selected solvent (0.5 mL, see Table S2) were added to the vial under argon flush. The reaction mixture was heated at 50 °C for 1 hour. The reaction vessel was allowed to cool down to RT. An NMR tube was filled with the crude mixture (ca 0.05 mL), DMSO-d_6 (0.5 mL), and ^{1}H NMR spectroscopy was conducted to determine the conversion and yield of the reaction. The Si-OH proton signal integral (5.88 ppm, 1H) was set to 1.00 and compared to the characteristic peak value in mesitylene (6.76 ppm, 3H).

Table S2 – Screening of different solvents.

Solvent	Yield	Conversion
EtOAc	96	100
Acetone	89	100
**THF**	**>99**	**100**
Heptane	40	100
Toluene	55	100
Chloroform	74	100
Acetonitrile	27	40
**2-MTHF**	**99**	**100**
DMSO	62	100

2.3. H_2O Stoichiometry Screening

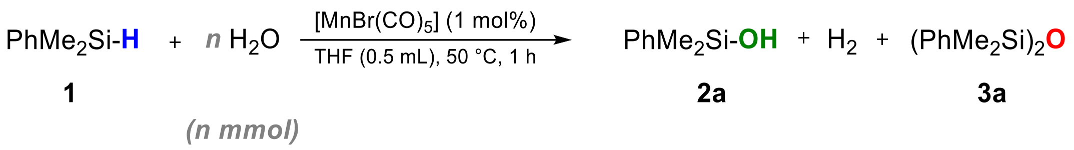

[Mn(CO)$_5$Br] (1.3 mg, 0.005 mmol), mesitylene (20 µL, 0.14 mmol), and dimethylphenylsilane (68.1 mg, 0.5 mmol) were weighed in a 4 mL vial equipped with a screw cap and a magnetic stirrer inside the glovebox. The reaction vessel was taken outside the glovebox, placed in a Schlenk tube previously evacuated, and filled with argon three times. The selected amount of H_2O (see Table S3) and THF (0.5 mL) were added to the vial under argon flush. The reaction mixture was heated at 50 °C for 1 hour. The reaction vessel was allowed to cool down to RT. An NMR tube was filled with the crude mixture (ca 0.05 mL), DMSO-d_6 (0.5 mL), and ^{1}H NMR spectroscopy was conducted to determine the conversion and yield of the reaction. The Si-OH proton signal integral (5.88 ppm, 1H) was set to 1.00 and compared to the characteristic peak value in mesitylene (6.76 ppm, 3H).

Table S3 – H_2O stoichiometry screening.

H_2O (µL)	H_2O equiv.	Time	Yield	Conversion
45	5	1h	>99	100
36	4	1h	>99	100
**27**	**3**	1h	**>99**	**100**
18	2	1h	90	100
9	1	1h	78	100

2.4. Screening of other Manganese carbonyl complexes

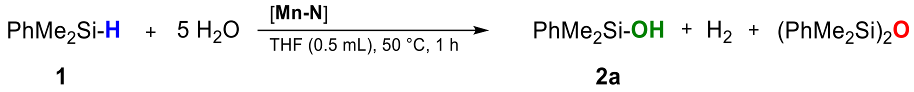

Selected manganese complex (see Table S4), mesitylene (20 µL, 0.14 mmol), and dimethylphenylsilane (68.1 mg, 0.5 mmol) were weighed in a 4 mL vial equipped with a screw cap and a magnetic stirrer inside the glovebox. The reaction vessel was taken outside the glovebox, placed in a Schlenk tube previously evacuated, and filled with argon three times. H_2O (45 µL, 2.5 mmol) and THF (0.5 mL) were added to the vial under argon. The reaction mixture was heated at 50 °C for 1 hour. The reaction vessel was allowed to cool down to RT. An NMR tube was filled with the crude mixture (ca 0.05 mL), DMSO-*d*$_6$ (0.5 mL), and ^{1}H NMR spectroscopy was conducted to determine the conversion and yield of the reaction. The Si-OH proton signal integral (5.88 ppm, 1H) was set to 1.00 and compared to the characteristic peak value in mesitylene (6.76 ppm, 3H). Manganese complexes **Mn-2**,[1] **Mn-3**,**[2]** and **Mn-4[3]** were prepared by following reported literature procedures. In contrast, **Mn-1** was purchased from Sigma Aldrich and was used as received.

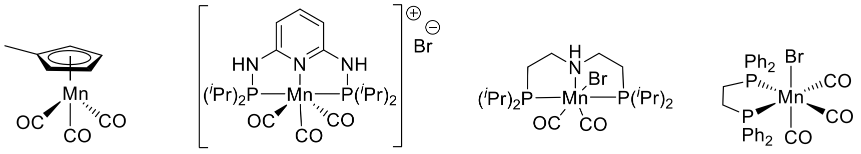

Mn-1 **Mn-2** **Mn-3** **Mn-4**

Table S4: Screening of other Mn-carbonyl complexes in the reaction.

Complex	Yield	Conversion
**Mn-1** (2 mol%)	0	0
**Mn-2** (1 mol%)	0	0
**Mn-2*** (1 mol%)	0	0
**Mn-3** (1 mol%)	0	0
**Mn-4** (1 mol%)	0	0
**Mn-4**** (1 mol%)	0	0
$[Mn_2(CO)_{10}]$	0	0
**$[MnBr(CO)_5]$**	**>99**	**>99**

*Repeated in DMSO at 50 °C
**Repeated in 2-MTHF at 80 °C

2.5. Screening of other Mn(X) complexes

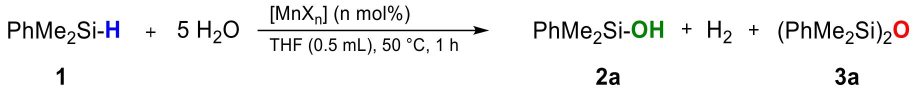

Selected manganese complex and associated additives (see Table S4), mesitylene (20 µL, 0.14 mmol), and dimethylphenylsilane (68.1 mg, 0.5 mmol) were weighed in a 4 mL vial equipped with a screw cap and a magnetic stirrer inside the glovebox. The reaction vessel was taken outside the glovebox, placed in a Schlenk tube previously evacuated, and filled with argon three times. H_2O (45 µL, 2.5 mmol) and THF (0.5 mL) were added to the vial under argon. The reaction mixture was heated at 50 °C for 1 hour. The reaction vessel was allowed to cool down to RT. An NMR tube was filled with the crude mixture (ca 0.05 mL), DMSO-d_6 (0.5 mL), and ^{1}H NMR spectroscopy was conducted to determine the conversion and yield of the reaction. The Si-OH proton signal integral (5.88 ppm, 1H) was set to 1.00 and compared to the characteristic peak value in mesitylene (6.76 ppm, 3H).

Table S5: Screening of Mn(X) complexes in the reaction.

Entry	Additive (mol %)	Yield	Conversion
20	[$MnCl_2(THF)_2$] (1 mol%)	0	0
21*	MnF_3 (1 mol%)*	0	0
22	$MnBr_2$ (1 mol%)	0	0
23	[$Mn(OAc)_3(H_2O)_2$] (1 mol%)	0	0
24*	$MnCl_2$ (1 mol%)	0	0
25	$MnBr_2$ (1 mol%) + 3 LiCl	0	0
26	$MnCl_2$ (2 mol%) + 3 LiCl	0	0

*Double scale

3. Reaction Scope

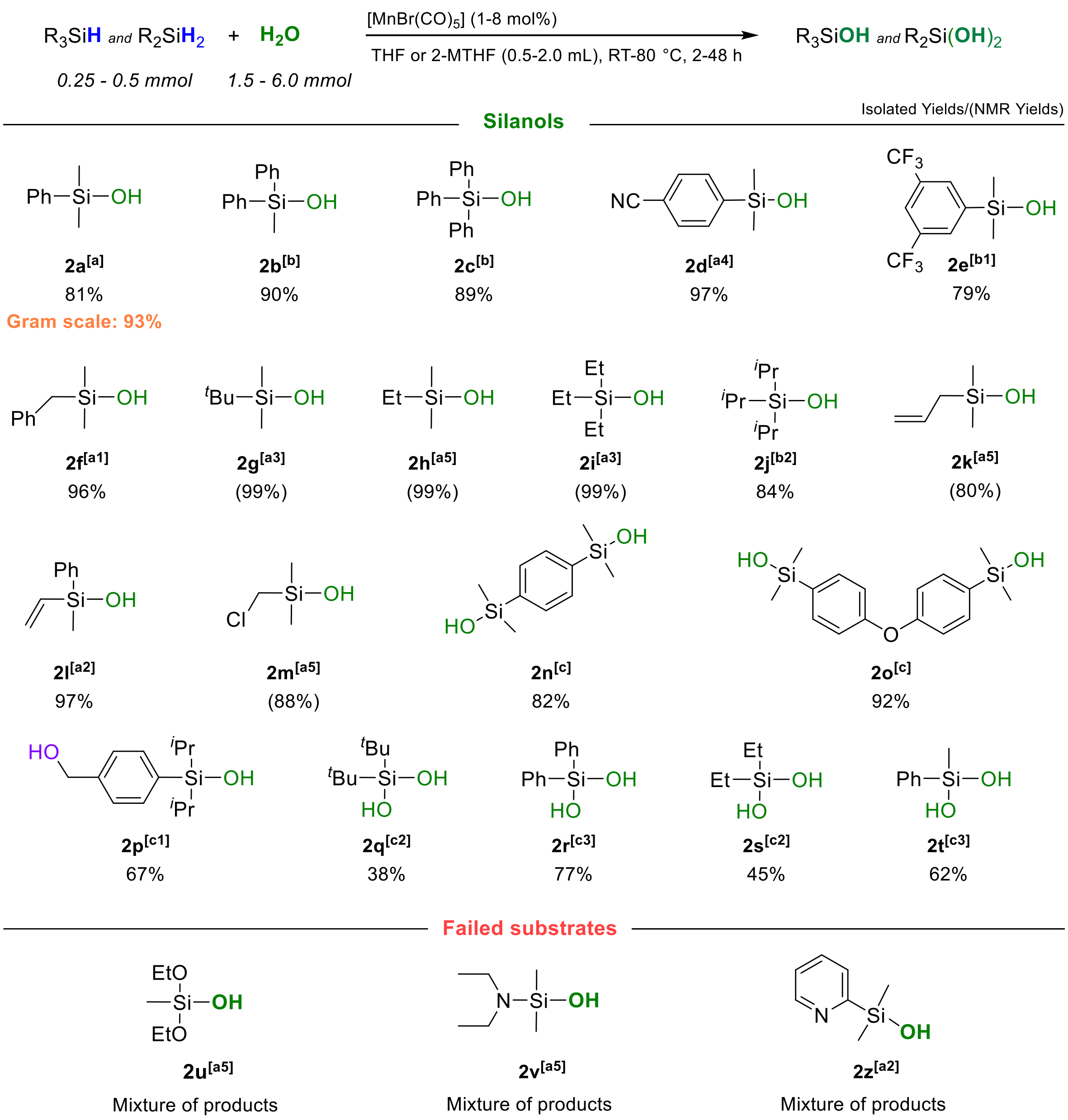

Scheme S1: Scope of the reaction: isolated yields are given below each respective silanol **2a-t**; NMR yields (in parentheses) were determined relative to mesitylene as an internal standard; The isolated Gram-scale synthesis yield is indicated in orange. **[a]:** $[MnBr(CO)_5]$ = 1 mol%, H_2O (1.5 mmol), T = 50 °C, silane (0.5 mmol), Solvent = THF (0.5 mL), time = 1 h; **[a1]:** time = 2 h; **[a2]:** time = 4 h; **[a3]:** time = 24 h; **[a4]:** time = 4 h , T = 60 °C; **[a5]:** time = 24 h, T = RT; **[b]:** see [a] with deviations: solvent = 2-MTHF and T = 80 °C; **[b1]:** see [b] with deviations: $[MnBr(CO)_5]$ = 2 mol %; **[b2]:** see [b] with deviations: $[MnBr(CO)_5]$ = 2 mol %, time = 5 h; **[c]:** $[MnBr(CO)_5]$ = 2 mol%, H_2O (6 mmol), T = 50 °C, silane (0.25 mmol), Solvent = THF (2.0 mL), time = 4 h; **[c1]:** see [c] with deviations: time = 48 h; **[c2]:** see [c1] with deviations: solvent = THF (1.0 mL), $[MnBr(CO)_5]$ = 4 mol%; **[c3]:** see [c2] with deviations: T = RT and $[MnBr(CO)_5]$ = 8 mol%.

3.1. Procedure (a)

[Mn(CO)$_5$Br] (1.3 mg, 0.005 mmol) and the indicated silane (0.5 mmol) were weighed in a 4 mL vial equipped with a screw cap and a magnetic stirrer inside the glovebox. The reaction vessel was taken outside the glovebox, placed in a Schlenk tube previously evacuated, and filled with argon three times. H_2O (27 μL, 1.5 mmol) and the indicated solvent (0.5 mL) were added to the vial under argon flush. The reaction mixture was heated at the indicated temperature and time. The reaction vessel was allowed to cool down to RT. The vial was carefully opened to release the H_2 overpressure and stirred open to the air for 10-15 min. The vial was closed and irradiated with blue light (450 nm) until the color of the solution changed from yellow to colorless with brown particles of $MnO_{2(s)}$. Activated 3Å molecular sieves were added to the reaction mixture to remove the excess water. The reaction mixture was filtered through a syringe filter, and the vial was washed with DCM (5 mL). The solvent was removed *in vacuo* to provide the target compound.

Table S6: table of conditions employed for the respective substrates using procedure (a).

Substrate	Solvent (volume)	T (°C)	Time (h)
**2c**	2-MTHF (0.5 mL)	80	1
**2e**	2-MTHF (0.25 mL)	80	1
**2f**	THF (0.5 mL)	50	2
**2j***	2-MTHF(0.5 mL)	80	5

* = 2 mol% of [MnBr(CO)$_5$]

3.2. Procedure (b)

The indicated silane (0.5 mmol) was weighed in a 4 mL vial equipped with a screw cap and a magnetic stirrer inside the glovebox. The reaction vessel was taken outside the glovebox, placed in a Schlenk tube previously evacuated, and filled with argon three times. H_2O (27 μL, 1.5 mmol) and THF (0.25 mL) were added to the vial under argon flush. A freshly prepared solution of [MnBr(CO)$_5$] (0.25 mL, 0.02 M in THF) was added. The reaction mixture was heated at the indicated temperature and time (see Table S7). The reaction vessel was allowed to cool down to RT. The vial was carefully opened to release the H_2 overpressure and stirred open to the air for 10-15 min. The vial was closed and irradiated with blue light (450 nm) until the color of the solution changed from yellow to colorless with brown particles of $MnO_{2(s)}$. Activated 3Å molecular sieves were added to the reaction mixture to remove the excess water. The reaction mixture was filtered through a syringe filter, and the vial was washed with DCM (5 mL). The solvent was removed *in vacuo* to provide the target compound.

Table S7 table of conditions employed for the respective substrates using procedure (b).

Substrate	Solvent (volume)	T (°C)	Time (h)
**2a**	THF (0.5 mL)	50	1
**2b**	THF (0.5 mL)	50	1
**2d**	THF (0.5 mL)	60	4
**2l**	THF (0.5 mL)	50	4

3.3. Procedure (c)

MnBr(CO)$_5$] (1.3 mg, 0.005 mmol) and the indicated silane (0.5 mmol) were weighed in two separate 4 mL vials inside the glovebox and equipped with a screw cap. The reaction vials were taken outside the glovebox, placed in a Schlenk tube previously evacuated, and filled with argon three times. H_2O (108 μL, 6 mmol) and THF (0.25 mL) were added, under argon flush, to the vial containing [MnBr(CO)$_5$]. The silane was dissolved in THF (0.5 mL), and the solution was added to the vial containing [MnBr(CO)$_5$] and H_2O together with additional THF (1 mL). The reaction mixture was heated at 50 °C for 4 hours (substrates **2n** and **2o**) or 48 hours (substrate **2p**). The reaction vessel was allowed to cool down to RT. The vial was carefully opened to release the H_2 overpressure and stirred open to the air for 10-15 min. The vial was closed and irradiated with blue light (450 nm) until the color of the solution changed from yellow to colorless with brown particles of $MnO_{2(s)}$. Activated 3Å molecular sieves were added to the reaction mixture to remove the excess water. The reaction mixture was filtered through a syringe filter, and the vial was washed with DCM (5 mL). The solvent was removed *in vacuo* to provide the target compound. Compound **2p** was further recrystallized in cyclohexane after filtration.

3.4. Procedure (d)

The indicated silane (0.5 mmol, substrate **2s**; 0.25 mmol substrates **2q**, **2r**, and **2t**) was weighed in a 4 mL vial equipped with a screw cap and a magnetic stirrer inside the glovebox. The reaction vessel was taken outside the glovebox, placed in a Schlenk tube previously evacuated, and filled with argon three times. H_2O (108 μL, 6 mmol) and THF (0.5 mL) were added to the vial under argon flush. A freshly prepared solution of [MnBr(CO)$_5$] (0.5 mL, 0.02 M in THF) was added. The reaction mixture was heated at 50 °C for 48 hours (substrates **2q** and **2s**) or RT for 48 hours (substrates **2r** or **2t**). The reaction vessel was allowed to cool down to RT (if heated). The vial was carefully opened to release the H_2 overpressure and stirred open to the air for 10-15 min. The vial was closed and irradiated with blue light (450 nm) until the color of the solution changed from yellow to colorless with brown particles of $MnO_{2(s)}$. Activated 3Å molecular sieves were added to the reaction mixture to remove the excess water. The reaction mixture was filtered through a syringe filter, and the vial was washed with THF (5 mL). The solvent was concentrated *in vacuo,* and products **2r**, **2s**, and **2t** were precipitated with pentane and washed with cold pentane (5 mL, 3 times).

Product **2q** was purified via silica gel column chromatography using a Pentane:EtOAc = 3:1 mixture of solvents.

3.5. NMR yields

Mesitylene (20 µL, 0.14 mmol) and the indicated silane (0.5 mmol) were weighed in a 4 mL vial equipped with a screw cap and a magnetic stirrer inside the glovebox. The reaction vessel was taken outside the glovebox, placed in a Schlenk tube previously evacuated, and filled with argon three times. H_2O (27 µL, 1.5 mmol) and THF (0.25 mL) were added to the vial under argon flush. A freshly prepared solution of [MnBr(CO)$_5$] (0.25 mL, 0.02 M in THF) was added. The reaction mixture was heated at 50 °C for 4 hours (**2z**) or 24 hours (substrates **2g** and **2i**) or RT for 24 hours (substrates **2h, 2k, 2m, 2u**, and **2v**). The reaction vessel was allowed to cool down to RT (if heated). An NMR tube was filled with the crude mixture (ca 0.05 mL), DMSO-d_6 (0.5 mL), and ^{1}H NMR spectroscopy was conducted to determine the conversion and yield of the reaction. The Si-OH proton signal integral (5.88 ppm, 1H) was set to 1.00 and compared to the characteristic peak value in mesitylene (6.76 ppm, 3H).

4. Synthesis of non-commercial substrates

4.1. Synthesis of $PhMe_2SiD$

The synthesis was carried out following slightly changed literature procedures.[4]*

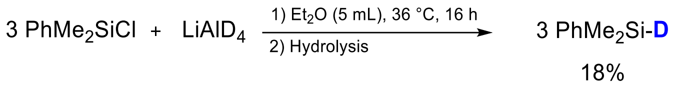

$LiAlD_4$ (44.9 mg, 1.0 mmol) was placed in a Schlenk tube equipped with a magnetic stirrer and a screw cap. $PhMe_2SiCl$ (494.6 mg, 2.9 mmol) was weighed in a vial inside the glovebox. Et_2O (3 mL) was added to $LiAlD_4$ to form a suspension. $PhMe_2SiCl$ was added dropwise to the suspension. The vial was washed with Et_2O (4*0.5 mL) and added to the Schlenk tube. The resulting mixture was heated at 36 °C for 16 hours. A $NaOH_{(aq)}$ solution (3 mL, 10% w/w) was added dropwise to the crude reaction mixture and stirred for 15 minutes. The aqueous phase turned into an emulsion and was extracted with Et_2O (4*5 mL). The solvent was evaporated, and $PhMe_2SiD$ was isolated via vacuum transfer as a colorless oil (69.6 mg, 0.51 mmol, 18%).

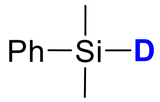

Dimethylphenylsilane-*d*[4]

$C_8H_{11}SiD$, colorless oil.

^{1}H NMR (400 MHz, Chloroform-*d*) δ 7.60 – 7.50 (m, 2H), 7.43 – 7.31 (m, 3H), 0.34 (s, 6H). ^{13}C NMR (101 MHz, Chloroform-*d*) δ 137.57 (t, *J* = 1.0 Hz), 134.15, 129.33, 128.00, -3.70 – -3.75 (t, J = 1.1 Hz). ^{29}Si NMR (79 MHz, Chloroform-*d*) δ -17.47 (t, J = 28.7 Hz).

4.2. Synthesis of ^{13}C-enriched [MnBr(CO)$_5$]

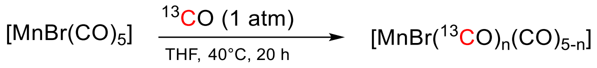

[MnBr(CO)$_5$] (56.4 mg, 0.2 mmol) was dissolved in the minimum amount of THF (ca. 1-1.5 mL). The solution was frozen with $N_{2(l)}$, evacuated, and refilled with an atmospheric pressure of $^{13}CO_{(g)}$. The solution was stirred overnight at 40 °C, cooled down at room temperature, and the solvent was removed *in vacuo* until a dry solid was obtained.

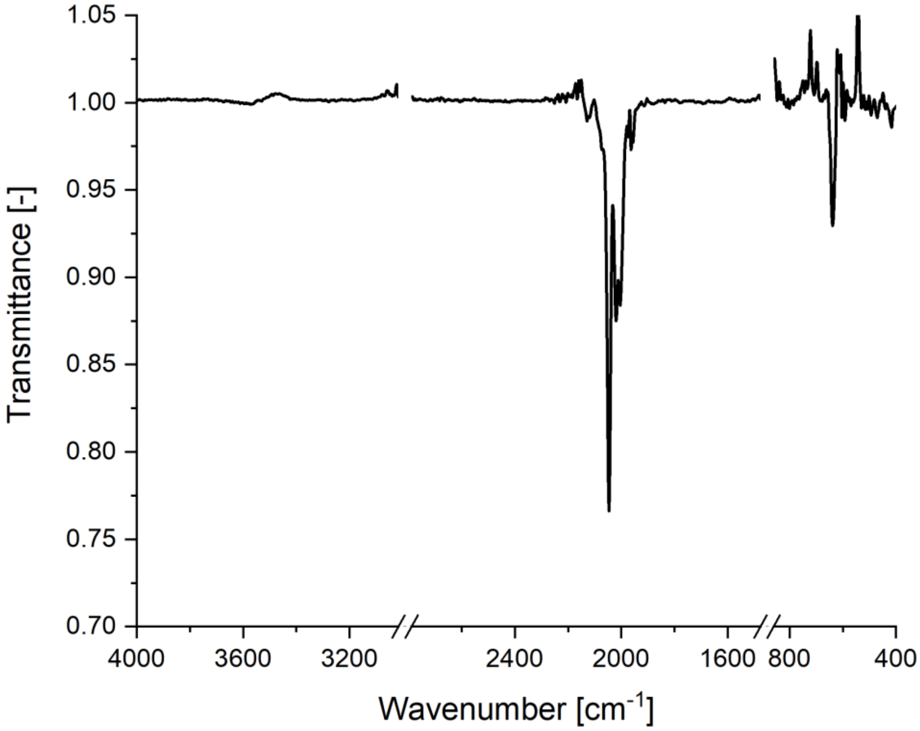

Figure S1: FT-IR spectrum of [MnBr(^{13}CO)$_n$(CO)$_{5-n}$] 2 mM in THF.

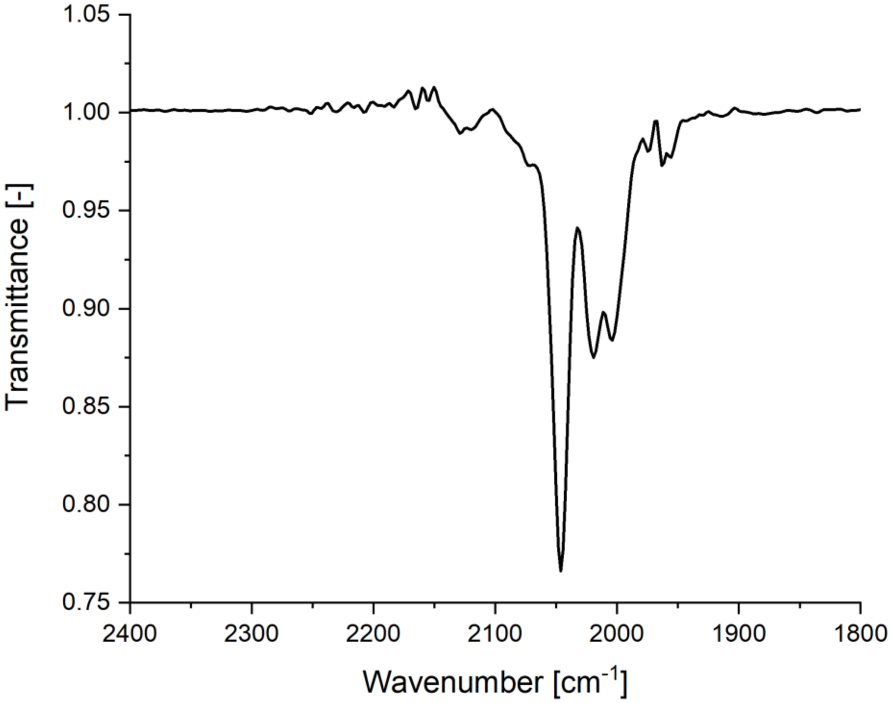

Figure S2: Detail of the carbonyl region of the FT-IR spectrum of [MnBr(^{13}CO)$_n$(CO)$_{5-n}$] 2 mM in THF.

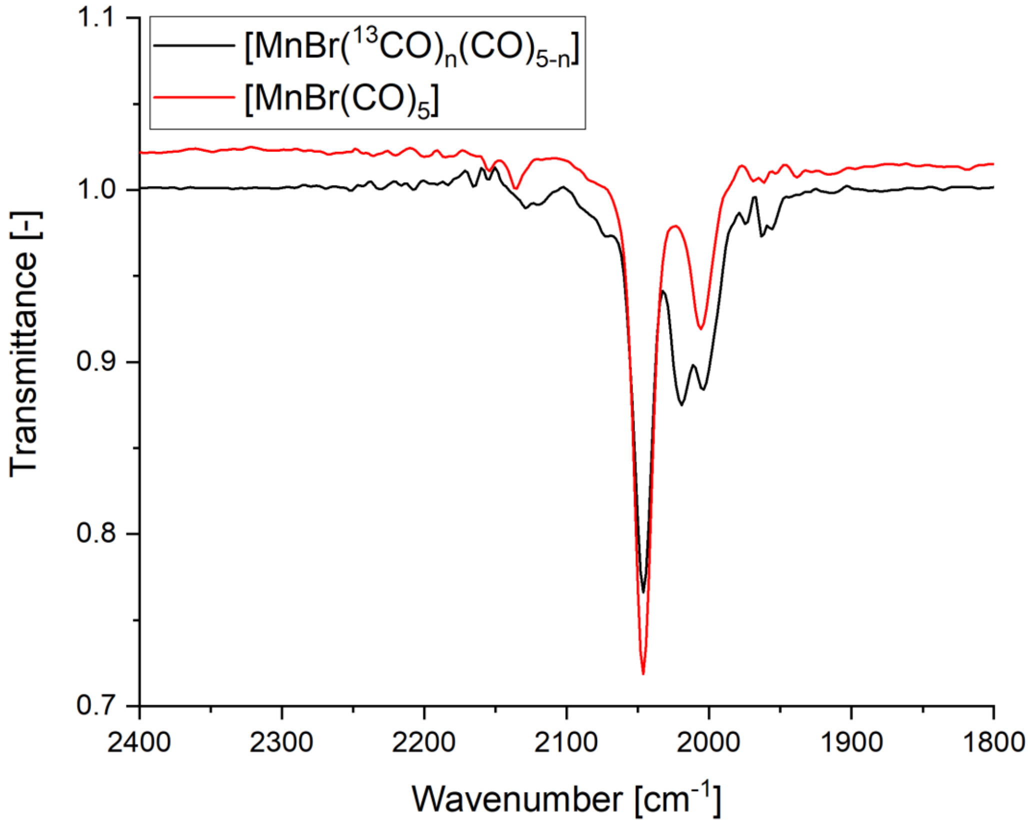

Figure S3: Detail of the carbonyl region of the FT-IR spectrum of $[MnBr(^{13}CO)_n(CO)_{5-n}]$ 2 mM in THF (in black) compared with $[MnBr(CO)_5]$ 2 mM in THF (in red).

5. Characterization Data

The different NMR spectra are consistent with those previously reported in the literature.

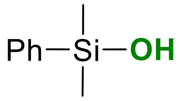

Dimethylphenylsilanol **(2a)**[5]

$C_8H_{12}OSi$, colorless oil.

^{1}H NMR (400 MHz, Chloroform-*d*, 296 K) δ 7.69 – 7.50 (m, 2H), 7.39 (m, 3H), 1.78 (s, 1H), 0.42 (s, 6H). ^{13}C{^{1}H} NMR (101 MHz, Chloroform-*d*, 296 K) δ 139.23, 133.18, 129.81, 128.06, 0.14. ^{29}Si NMR (79 MHz, Chloroform-*d*) δ 7.95.

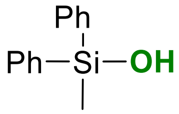

Methyldiphenylsilanol **(2b)**[5]

$C_{13}H_{14}OSi$, colorless oil.

^{1}H NMR (400 MHz, Chloroform-*d*, 296 K) δ 7.70 – 7.56 (m, 4H), 7.52 – 7.30 (m, 6H), 2.24 – 2.05 (s, br, 1H), 0.68 (s, 3H). ^{13}C NMR (101 MHz, Chloroform-*d*, 296 K) δ 137.19, 134.11, 130.06, 128.08, -1.09.^{29}Si NMR (79 MHz, Chloroform-*d*) δ -2.42.

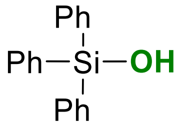

Triphenylsilanol **(2c)**[5]

$C_{18}H_{16}OSi$, white solid.

^{1}H NMR (400 MHz, Chloroform-*d*, 296 K) δ 7.93 – 7.55 (m, 6H), 7.53 – 7.28 (m, 9H), 2.51 (s, br, 1H). ^{13}C NMR (101 MHz, Chloroform-*d*, 296 K) δ 135.25, 135.13, 130.29, 128.08. ^{29}Si NMR (79 MHz, Chloroform-*d*) δ -12.53.

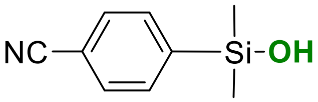

4-(hydroxydimethylsilyl)benzonitrile **(2d)**[6]

$C_9H_{11}NOSi$, colorless oil.

^{1}H NMR (400 MHz, Chloroform-*d*) δ 7.86 – 7.49 (m, 4H), 1.98 (s, br, 1H), 0.43 (s, 6H). ^{13}C NMR (101 MHz, Chloroform-*d*) δ 145.73, 133.68, 131.31, 119.03, 113.25, 0.08. ^{29}Si NMR (79 MHz, Chloroform-*d*) δ 7.45.

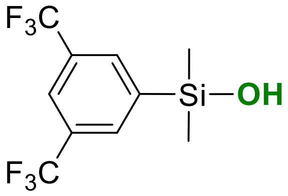

(3,5-bis(trifluoromethyl)phenyl)dimethylsilanol **(2e)**

$C_{10}H_{10}F_6OSi$, white solid.

HR-MS: m/z calculated for $[C_{10}H_{10}F_6OSi]^+$ = 288.03996, found = 288.03957.

^{1}H NMR (400 MHz, Chloroform-*d*) δ 8.01 (dd, *J* = 1.9, 1.0 Hz, 2H), 7.89 (dq, *J* = 1.8, 0.9 Hz, 1H), 2.01 (s, 1H), 0.47 (s, 6H).^{29}Si NMR (79 MHz, Chloroform-*d*) δ 7.40. ^{13}C NMR (101 MHz, Chloroform-*d*) δ 142.63, 133.06, 131.03 (q, *J* = 32.9 Hz), 124.18-122.77 (m), 123.70 (q, J = 272.9 Hz), 0.20. ^{19}F NMR (376 MHz, Chloroform-*d*) δ -62.83.

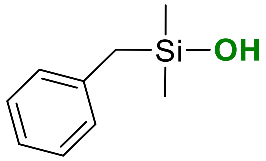

Benzyldimethylsilanol **(2f)**[5]

$C_9H_{14}OSi$, colorless oil.

^{1}H NMR (400 MHz, Chloroform-*d*, 296 K) δ 7.26 – 7.19 (m, 2H), 7.15 – 6.99 (m, 3H), 2.18 (s, 2H), 1.58 (s, 1H), 0.14 (s, 6H).^{13}C NMR (101 MHz, Chloroform-*d*, 296 K) δ 139.11, 128.59, 128.29, 124.46, 28.17, -0.55.^{29}Si NMR (79 MHz, Chloroform-*d*) δ 14.42.

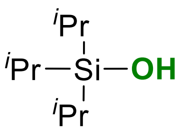

Triisopropylsilanol **(2j)**[7]

$C_9H_{22}OSi$, colorless oil.

^{1}H NMR (400 MHz, Chloroform-*d*, 296 K) δ 1.30 (s, 1H), 1.05 (m, 21H). ^{13}C NMR (101 MHz, Chloroform-*d*, 296 K) ^{13}C NMR (101 MHz, Chloroform-*d*) δ 17.85, 12.42. ^{29}Si NMR (79 MHz, Chloroform-*d*) δ 15.11.

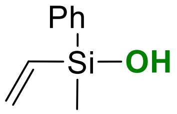

Methyl(phenyl)(vinyl)silanol **(2l)**[8]

$C_9H_{12}OSi$, colorless oil.

^{1}H NMR (400 MHz, Chloroform-*d*, 296 K) δ 7.65 – 7.55 (m, 2H), 7.45 – 7.34 (m, 3H), 6.31 (dd, *J* = 20.2, 14.8 Hz, 1H), 6.14 (dd, *J* = 14.9, 3.8 Hz, 1H), 5.89 (dd, *J* = 20.2, 3.8 Hz, 1H), 1.94 (s, 1H), 0.49 (s, 3H).^{13}C{^{1}H} NMR (101 MHz, Chloroform-*d*, 296 K) 137.36, 136.65, 134.75, 133.75, 130.00, 128.07, -1.55. ^{29}Si NMR (79 MHz, Chloroform-*d*) δ -4.12.

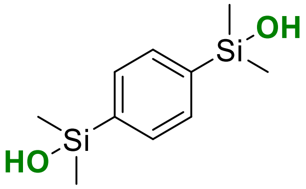

1,4-phenylenebis(dimethylsilanol) **(2n)**

$C_{10}H_{18}O_2Si_2$, white solid.

^{1}H NMR (400 MHz, Chloroform-*d*, 296 K) δ 7.62 (s, 4H), 1.80 (s, 2H), 0.41 (s, 12H).^{13}C NMR (101 MHz, Chloroform-*d*, 296 K) δ 140.80, 132.59, 0.11. ^{29}Si NMR (79 MHz, Chloroform-*d*) δ 7.85.

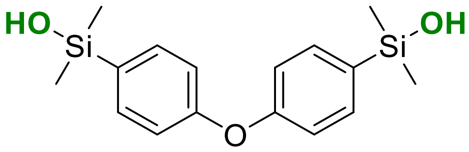

(oxybis(4,1-phenylene))bis(dimethylsilanol) **(2o)**

$C_{16}H_{22}O_3Si_2$, white solid.

^{1}H NMR (400 MHz, Chloroform-*d*, 296 K) δ 7.70 – 7.43 (m, 4H), 7.12 – 6.93 (m, 4H), 1.78 (s, 2H), 0.41 (s, 12H). ^{13}C{^{1}H} NMR (101 MHz, Chloroform-*d*, 296 K) δ 158.43, 134.98, 133.71, 118.62, 0.26. ^{29}Si NMR (79 MHz, Chloroform-*d*) δ 7.86.

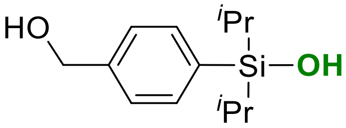

(4-(hydroxymethyl)phenyl)diisopropylsilanol **(2p)**

$C_{13}H_{22}O_2Si$, white solid.

HR-MS: m/z calculated for $[C_{13}H_{21}O_2Si]^-$ = 237.131634, found = 237.131660.

^{1}H NMR (400 MHz, Chloroform-*d*, 296 K) δ 7.56 (d, *J* = 7.9 Hz, 2H), 7.38 (d, *J* = 7.6 Hz, 2H), 4.72 (d, *J* = 5.9 Hz, 2H), 1.70 (s, 1H), 1.65 (t, *J* = 6.0 Hz, 1H), 1.22 (hept, *J* = 7.3 Hz, 2H), 1.05 (d, *J* = 7.3 Hz, 6H), 0.97 (d, *J* = 7.4 Hz, 6H).^{13}C NMR (101 MHz, Chloroform-*d*, 296 K) 142.05, 134.86, 134.54, 126.37, 65.52, 17.27, 17.02, 12.53. ^{29}Si NMR (79 MHz, Chloroform-*d*) δ 7.52.

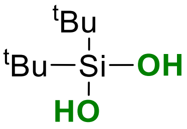

Di-*tert*-butylsilanediol **(2q)**[9]

$C_8H_{20}O_2Si$, white solid.

^{1}H NMR (400 MHz, DMSO-d_6) δ 5.50 (s, 2H), 0.93 (s, 18H).^{13}C NMR (101 MHz, DMSO-d_6) δ 27.66, 19.74.^{29}Si NMR (79 MHz, DMSO-d_6) δ -14.07.

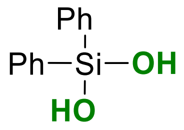

Diphenylsilanediol **(2r)**[5]

$C_{12}H_{12}O_2Si$, white solid.

^{1}H NMR (400 MHz, DMSO-*d*$_6$) δ 7.66 – 7.54 (m, 4H), 7.40 – 7.28 (m, 6H), 6.94 (s, 2H). ^{13}C NMR (101 MHz, DMSO-*d*$_6$) δ 137.80, 134.07, 129.35, 127.48. ^{29}Si NMR (79 MHz, DMSO-*d*$_6$) δ -34.90.

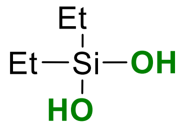

Diethylsilanediol **(2s)**[5]

$C_4H_{12}O_2Si$, white solid.

^{1}H NMR (400 MHz, DMSO-*d*$_6$) δ 5.66 (s, 2H), 0.88 (t, *J* = 8.0 Hz, 6H), 0.37 (q, *J* = 8.0 Hz, 4H). ^{13}C NMR (101 MHz, DMSO-*d*$_6$) δ 6.84, 6.59. ^{29}Si NMR (79 MHz, DMSO-*d*$_6$) δ -8.61.

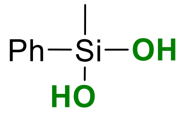

Methylphenylsilanediol **(2t)**[10]

$C_7H_{10}O_2Si$

^{1}H NMR (400 MHz, DMSO-*d*$_6$) δ 7.61 – 7.53 (m, 2H), 7.39 – 7.29 (m, 3H), 6.33 (s, 2H), 0.15 (s, 3H). ^{13}C NMR (101 MHz, DMSO-*d*$_6$) δ 139.41, 133.38, 129.10, 127.42, -0.53. ^{29}Si NMR (79 MHz, DMSO-*d*$_6$) δ -21.60.

6. Preparative Scale Reaction

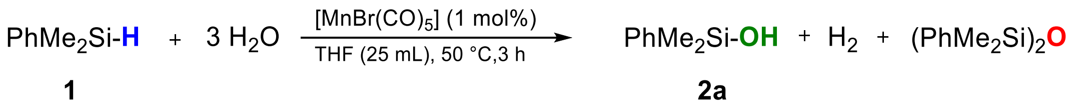

$[MnBr(CO)_5]$ (27.5 mg, 0.1 mmol) was dissolved in THF (13 mL) in a Schlenk tube equipped with a screw cap and a stirring bar inside the glovebox. $PhMe_2SiH$ (1.36 g, 10.0 mmol) was dissolved in THF (13 mL) in a different Schlenk tube inside the glovebox. Both Schlenk tubes were taken outside the glovebox and connected to the Schlenk line. H_2O (540 µL, 30 mmol) was added to the $[MnBr(CO)_5]$ solution in THF under argon flow using an Eppendorf pipette, followed by dropwise addition of the $PhMe_2SiH$ solution in THF (over 15 min) to the vigorously stirred solution of $[MnBr(CO)_5]$ and H_2O. The Schlenk tube containing $PhMe_2SiH$ was rinsed with 5 mL more of THF and added to the reaction vessel, which was placed in a pre-heated oil bath at 50 °C for 3 hours and was connected to the Schlenk line to prevent the development of overpressure. At the end of the reaction, the Schlenk tube was cooled down to room temperature, and the solvent was evaporated *in vacuo*. The resulting mixture was purified by silica gel column chromatography with Pentane:EtOAc = 9:1 as eluent to afford $PhMe_2SiOH$ as a colorless oil (1.41 g, 9.26 mmol, 93%).

7. Stoichiometric NMR experiments

7.1. Stoichiometric reaction between $PhMe_2SiH$ and $[MnBr(CO)_5]$

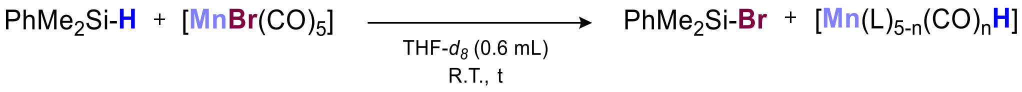

Scheme S2 Stoichiometric reaction between $PhMe_2SiH$ and $[MnBr(CO)_5]$.

$[MnBr(CO)_5]$ (10.9 mg, 0.04 mmol) and $PhMe_2SiH$ (5.5 mg, 0.04 mmol) were dissolved in THF-d_8 (0.6 mL) and placed in an oven-dried (120 °C) J-Young tube. ^{1}H- and ^{29}Si- NMR were recorded over time. First, the *"hydride-bromide exchange"* can be observed. Indeed, after 15 minutes, it is possible to observe the formation of $PhMe_2SiBr$ and the corresponding manganese hydride complex (Figure S4). Over time (Figure S5). It is possible to observe that after only 2 hours, $PhSiMe_2Br$ is completely consumed to afford different silicon-based species. Details of the NMR spectra are reported in the following figures.

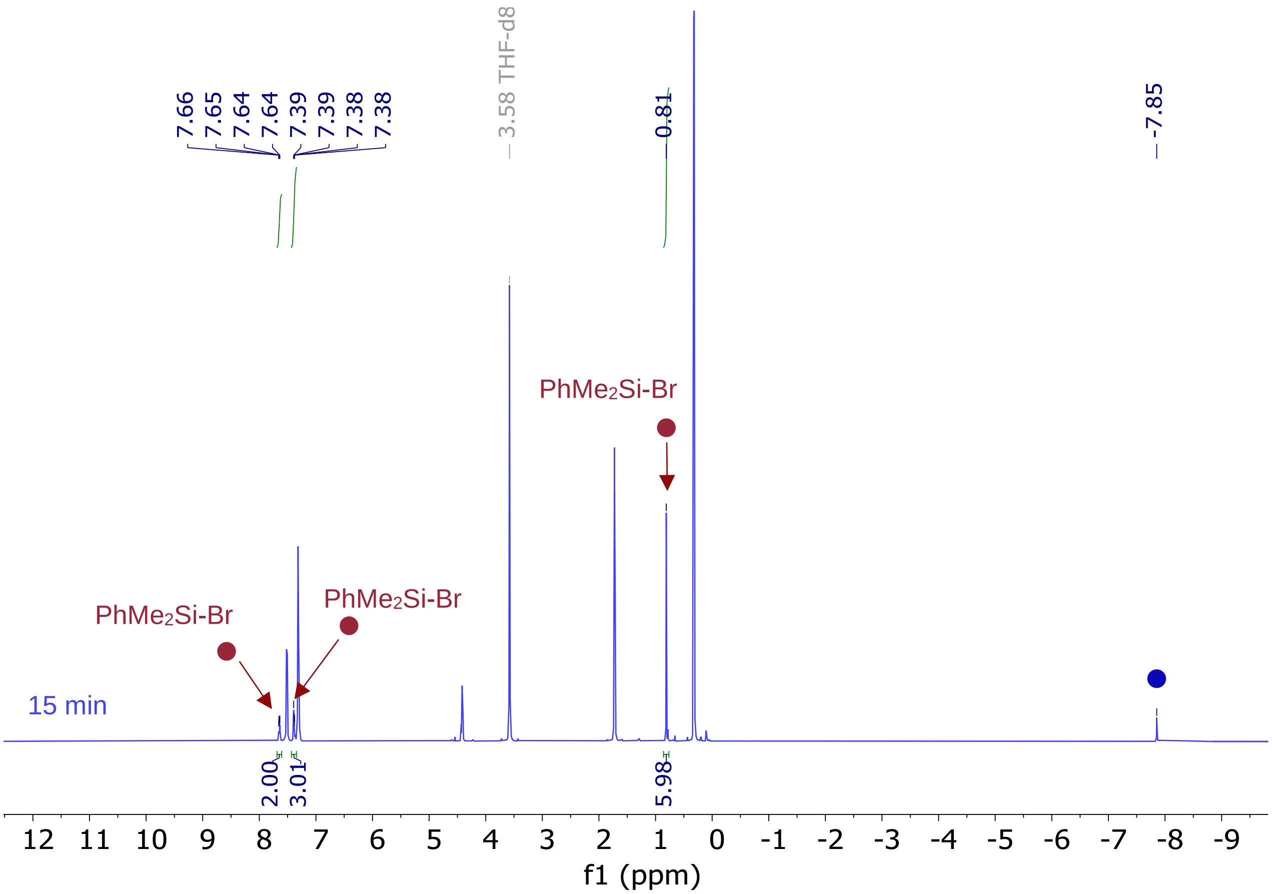

Figure S4: ^{1}H NMR (500 MHz, THF-d_8, 296 K) spectrum. Comparison with the reaction reported in Scheme S2 after 15 minutes. The signals highlighted with a red dot correspond to $PhMe_2SiBr$, and the one highlighted with a blue dot corresponds to the manganese hydride.

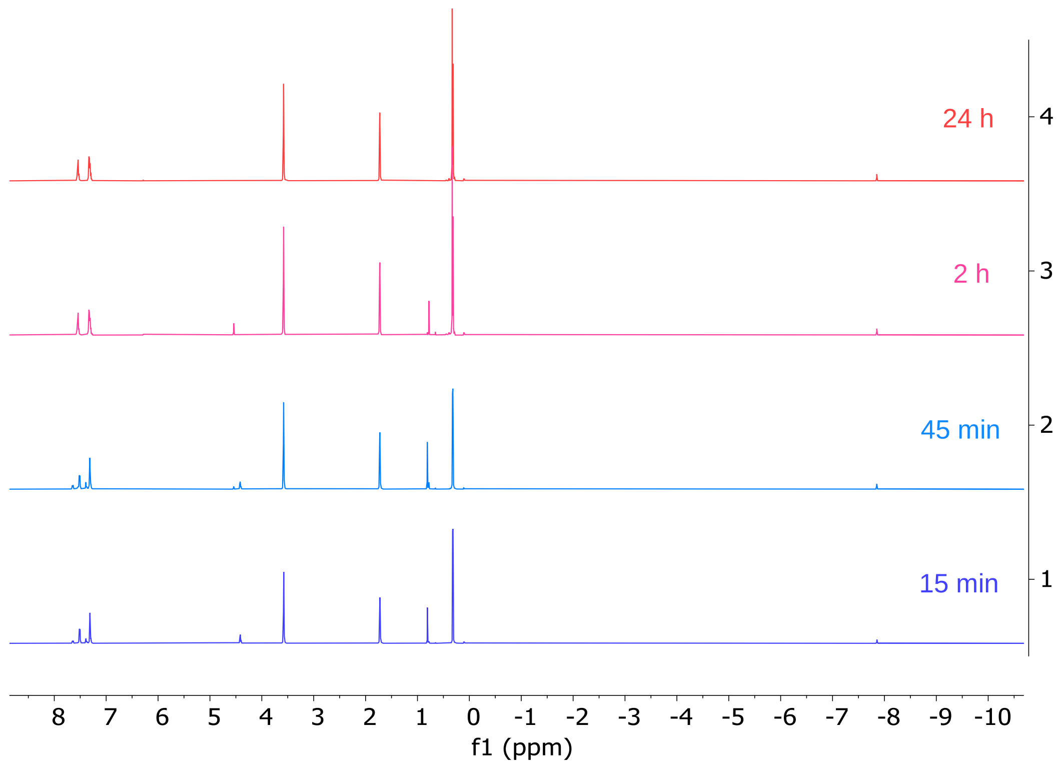

Figure S5: ^{1}H NMR (500 MHz, THF-d_8, 296 K) spectra. Comparison of the reaction reported in Scheme S2 at different times.

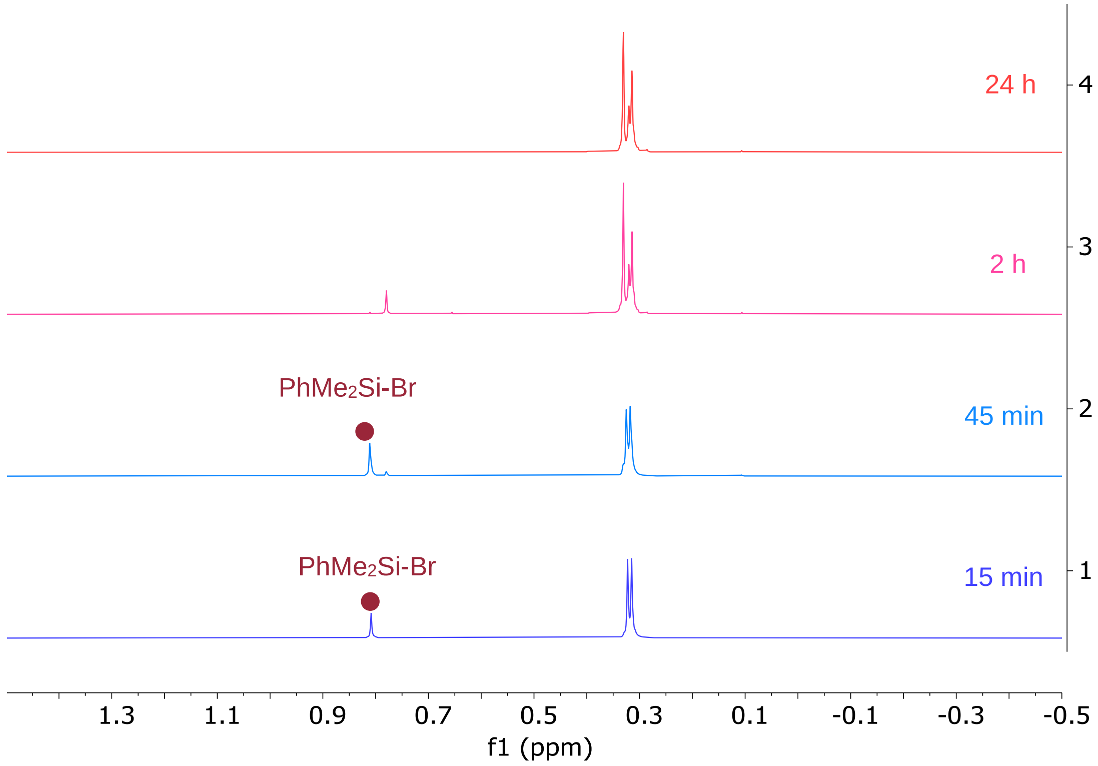

Figure S6: ^{1}H NMR (500 MHz, THF-d_8, 296 K) spectra of the region 1.5 to -0.5 ppm. Comparison of the reaction reported in Scheme S2 at different times. It is possible to observe the almost complete decomposition of $PhMe_2SiBr$ after only 2 hours. The signals highlighted with a red dot correspond to $PhMe_2SiBr$.

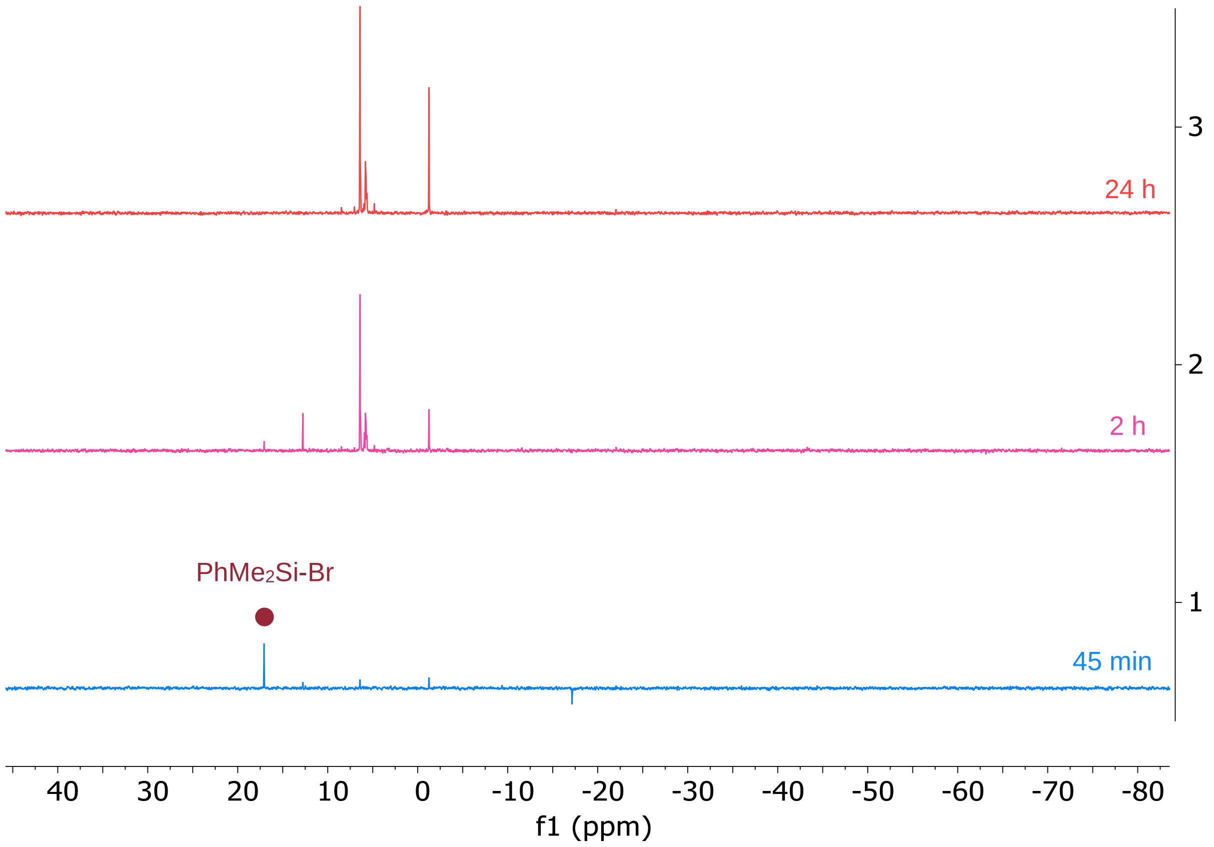

Figure S7: ^{29}Si NMR (79 MHz, THF-d_8, 296 K) spectra. Comparison of the reaction reported in Scheme S2 at different times. It is possible to observe the almost complete decomposition of $PhMe_2SiBr$ after only 2 hours. The signals highlighted with a red dot correspond to $PhMe_2SiBr$.

7.2. Comparison with the reaction with bromine

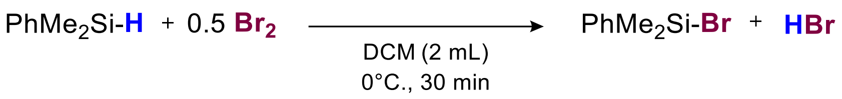

Scheme S3: Stoichiometric reaction between $PhMe_2SiH$ and Br_2.

Br_2 (74.9 mg, 0.5 mmol) was added to a Schlenk tube under an argon atmosphere. 2 mL of DCM were added to Br_2 in Ar flush, and the Schlenk tube was cooled down to 0 °C in an ice bath. $PhMe_2SiH$ (157.2 µL, 1.0 mmol) was added dropwise to the dark red solution and vigorously stirred under argon flow. The solution turns colorless and is stirred at 0 °C for 30 min. The Schlenk tube is allowed to warm up to RT over 15 min, and 0.25 mL of the reaction mixture is transferred to a J-Young tube evacuated and refilled with Ar 3 times. The solvent is evaporated *in vacuo*, and 0.5 mL of THF-d_8 is added to the Young tube. The crude reaction mixture (^{1}H- and ^{29}Si-NMR) is shown in black for comparison with the reaction between $[MnBr(CO)_5]$ and $PhMe_2SiH$.

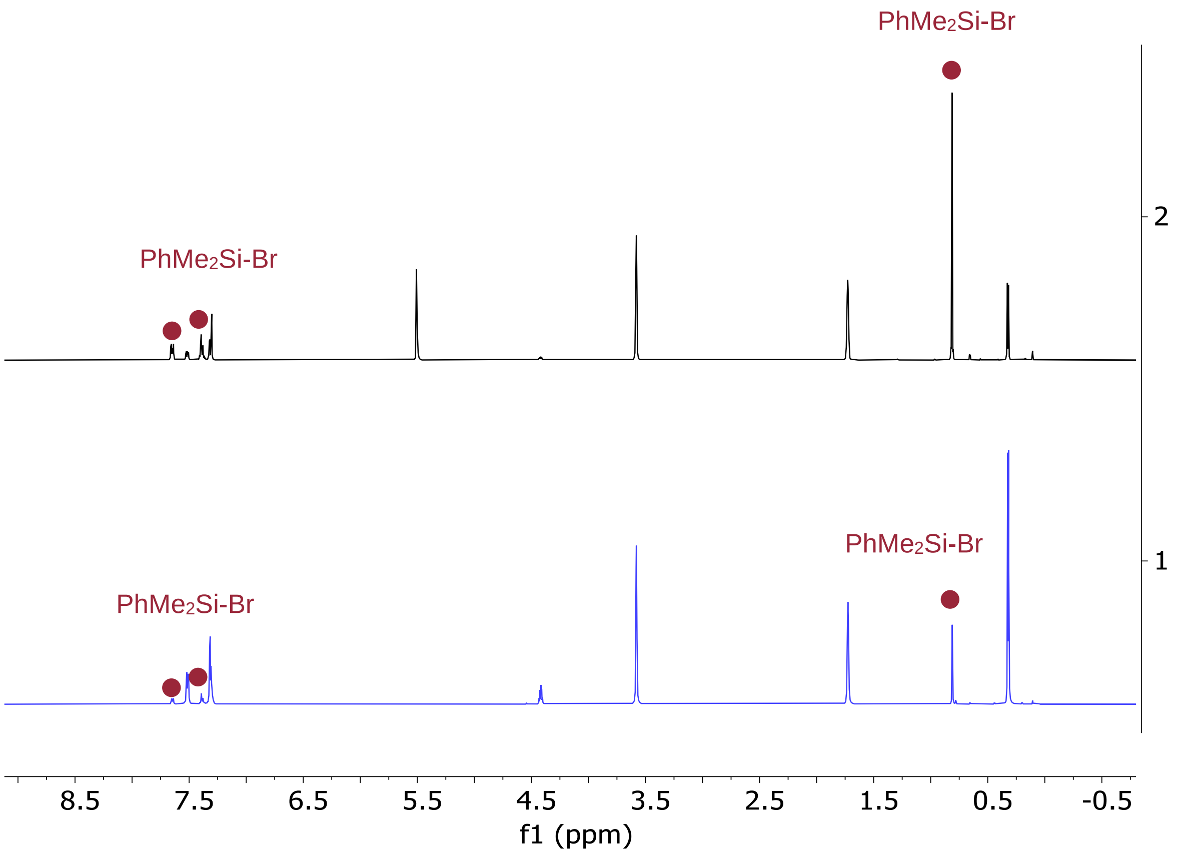

Figure S8: ^{1}H NMR (400 MHz, THF-d_8, 296 K) spectra. Comparison of the crude reaction mixture reported in **Scheme S3** (black, on top) with the reaction reported in Scheme S2 after 15 minutes (blue, below).

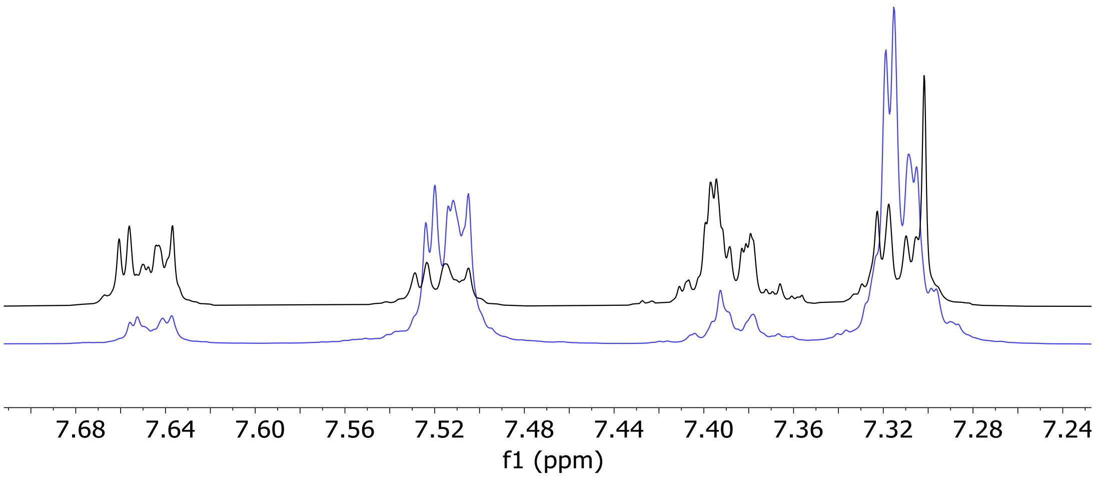

Figure S9: ^{1}H NMR (400 MHz, THF-d_8, 296 K) spectra of the region 7.70 – 7.23 ppm. Comparison of the crude reaction mixture reported in **Scheme S3** (black) with the reaction reported in Scheme S2 after 15 minutes (blue).

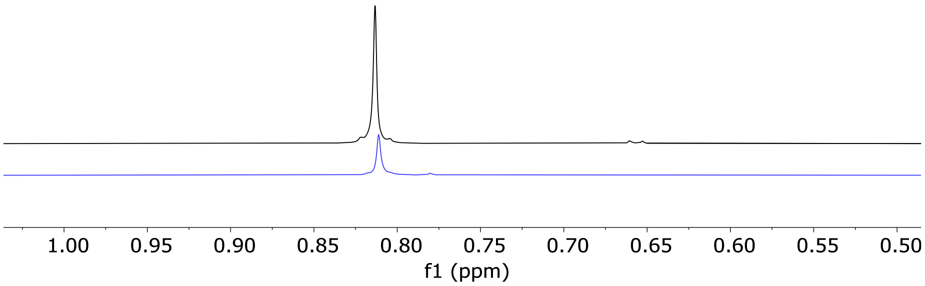

Figure S10: ^{1}H NMR (400 MHz, THF-d_8, 296 K) spectra of the region 1.05 – 0.50 ppm. Comparison of the crude reaction mixture reported in **Scheme S3** (black) with the reaction reported in Scheme S2 after 15 minutes (blue).

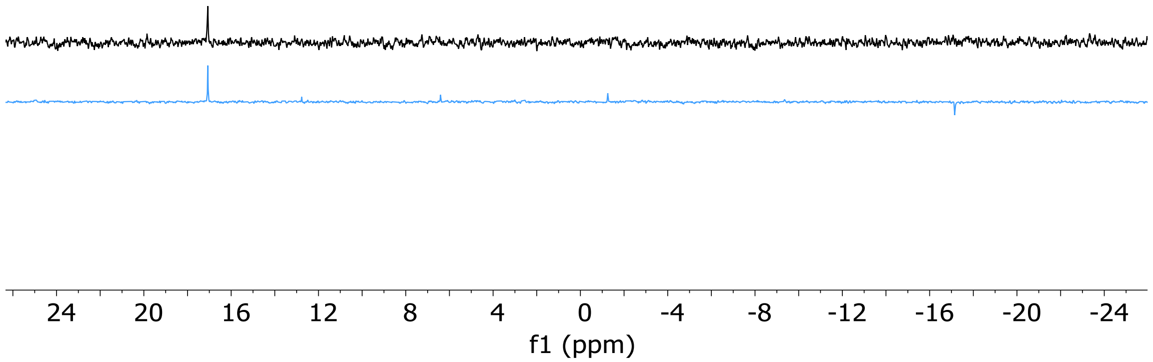

Figure S11: ^{29}Si NMR (79 MHz, THF-d_8, 296 K) spectra. Comparison of the crude reaction mixture reported in **Scheme S3** (black) with the reaction reported in Scheme S2 after 15 minutes (blue).

8. Kinetic studies

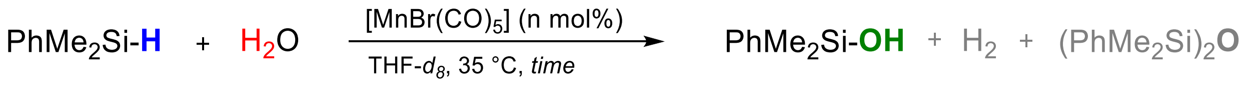

8.1. General procedure

Stock solutions of H_2O, $PhMe_2SiH$, and $[MnBr(CO)_5]$ in THF-d_8 were prepared in oven-dried (120 °C) volumetric flasks under an inert atmosphere (either by the use of Schlenk techniques or inside the glovebox). An oven-dried (120 °C) J-Young tube placed in a Schlenk tube was evacuated and refilled with argon 3 times. To reach the desired concentration in all the 3 the components (reported in Table S8), the chosen volume of stock solution for the H_2O solution in dry and degassed THF-d_8 was added with a Hamilton syringe to the J-Young tube under argon flow. The J-Young tube was closed, brought to the glovebox, and the chosen volume of stock solutions of $PhMe_2SiH$ and $[MnBr(CO)_5]$ were added using Hamilton syringes. The volume of the solution has been brought to 0.5 mL by further addition of dry and degassed THF-d_8. The J-Young tube was closed, and the sample was taken outside the glovebox and immediately placed in a Dewar filled with dry ice. The tube was placed in the probe-head of an NMR spectrometer pre-heated at 35 °C, and ^{1}H-NMR spectra were recorded until full conversion of $PhMe_2SiH$ was achieved. The order with respect to each component was determined by an initial rate method and confirmed visually via VTNA.[11] The kinetic

isotope effect (KIE) was determined by preparing the samples according to the same procedure but using stock solutions of D_2O and $PhMe_2SiD$ in dry and degassed THF-d_8.

Table S8: List of initial concentrations of each component for each run of the kinetic analysis.

**Run**	**[$PhMe_2SiH$]**	**[H_2O]**	**[$MnBr(CO)_5$]**	**[$PhMe_2SiOH$]**
1	100 mM	300 mM	1 mM	0 mM
2*	100 mM	300 mM	2 mM	0 mM
3	100 mM	300 mM	4 mM	0 mM
4	25 mM	300 mM	2 mM	0 mM
5	50 mM	300 mM	2 mM	0 mM
6*	100 mM	150 mM	2 mM	0 mM
7	100 mM	600 mM	2 mM	0 mM
8	25 mM	150 mM	2 mM	0 mM
9	50 mM	150 mM	2 mM	0 mM
10**	100 mM	300 mM	2 mM	0 mM
11***	100 mM	300 mM	2 mM	0 mM
12	100 mM	300 mM	2 mM	25 mM
13	100 mM	300 mM	2 mM	50 mM

*Repeated two times
** $PhMe_2SiD$ used instead of $PhMe_2SiH$
*** D_2O used instead of H_2O

8.2. Order in [$MnBr(CO)_5$]

Table S9: Concentration values for each component used to determine the order of [$MnBr(CO)_5$] in the reaction rate.

Run	[$PhMe_2SiH$]	[H_2O]	[$MnBr(CO)_5$]
1	100 mM	300 mM	1 mM
2*	100 mM	300 mM	2 mM
3	100 mM	300 mM	4 mM

*Repeated two times

The concentration plots obtained by NMR (Figure S12) show the concentration of $PhMe_2SiOH$ with time scales normalized to a zeroth- (top left), first- (top right), second- (bottom left) and 0.9th- (bottom right) order dependence on the initial catalyst concentration, indicated as [$MnBr(CO)_5$]$_0$. According to the variable time normalization analysis, the best fit of the time course for the formation of dimethylphenylsilanol was obtained for an order of 0.9 (bottom right graph, Figure S12). The data were also analyzed with the initial rates method (Figure S13). The initial reaction rate (r_0) for each run was determined as the slope of the concentration of silanol over time. An initial reaction order of 0.84 with respect to the [$MnBr(CO)_5$]$_0$ was obtained as the slope of the natural logarithm of the initial reaction rate of each run over the natural logarithm of the concentration of [$MnBr(CO)_5$] of the corresponding run.

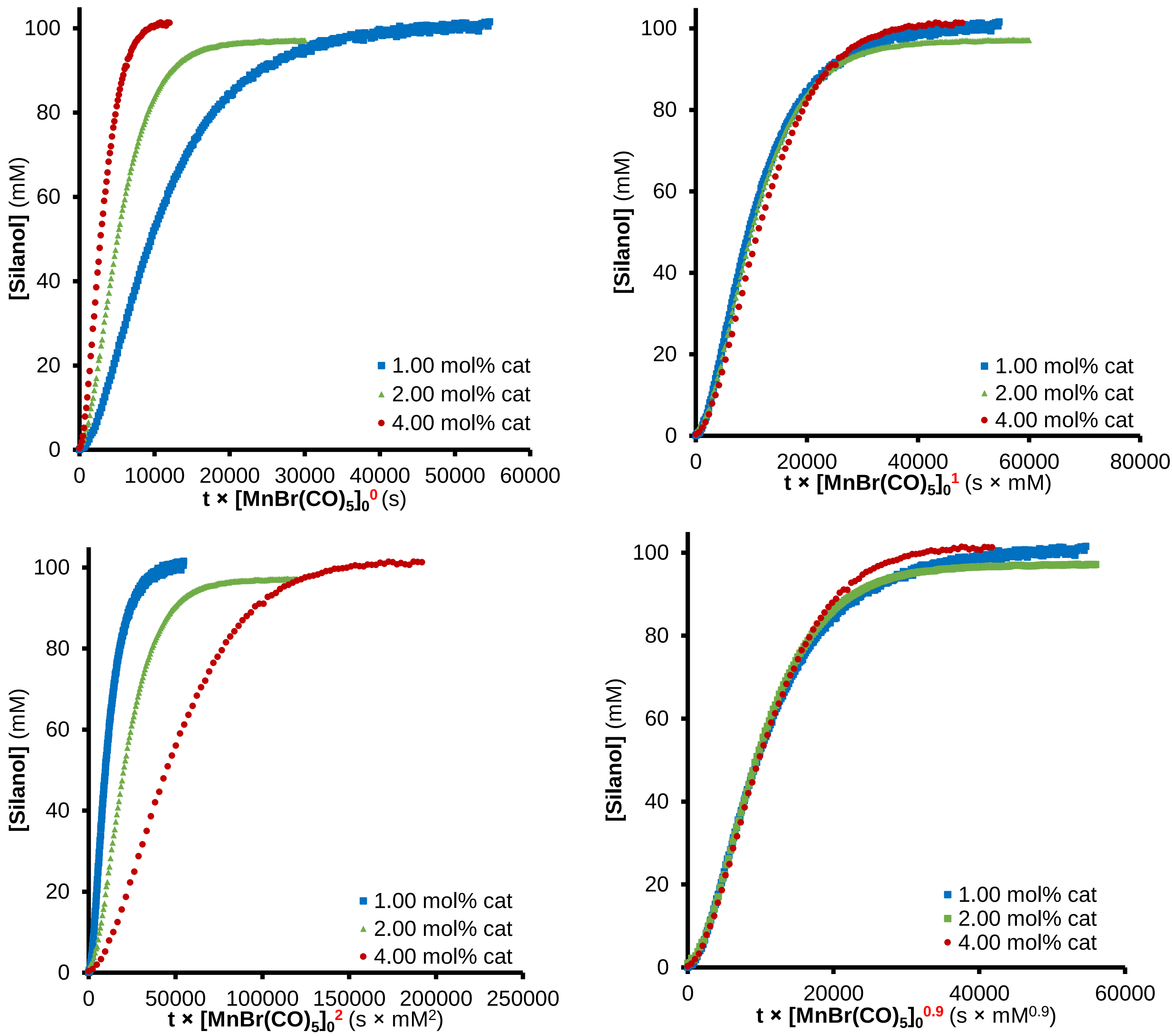

Figure S12: Plots obtained via VTNA of the NMR data where the initial concentration of [$MnBr(CO)_5$] is elevated to different exponents, as described in the above paragraph.

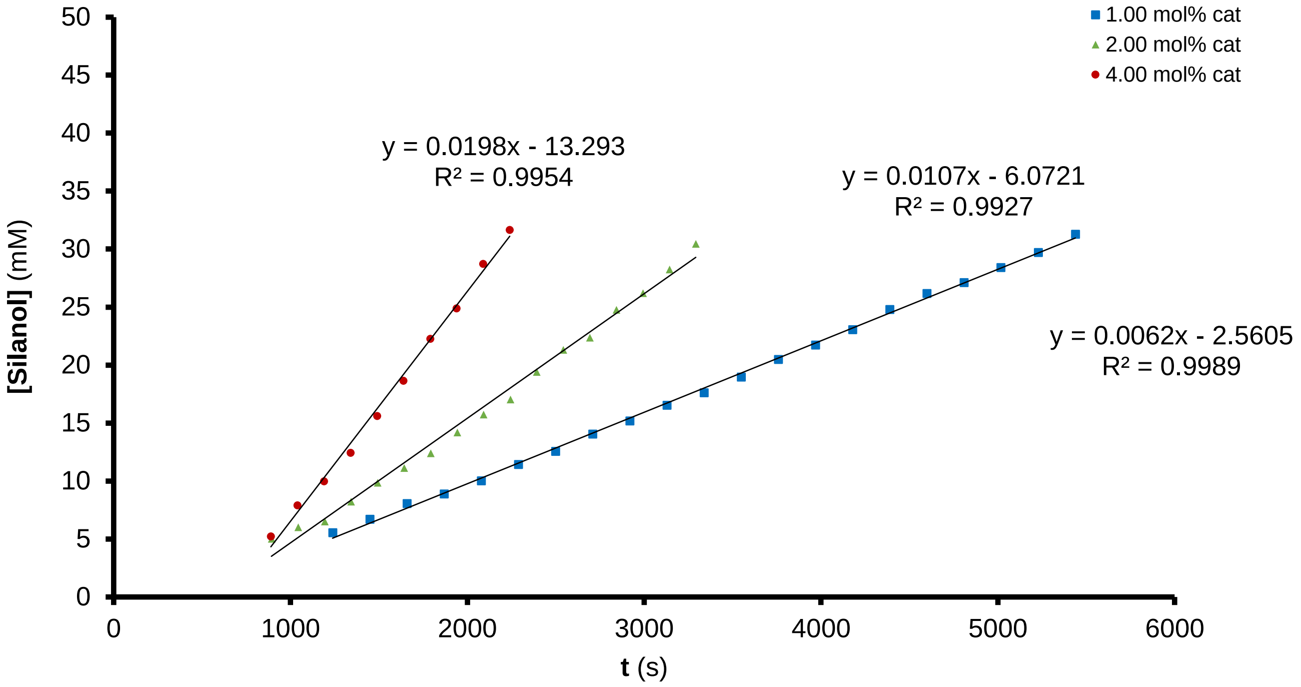

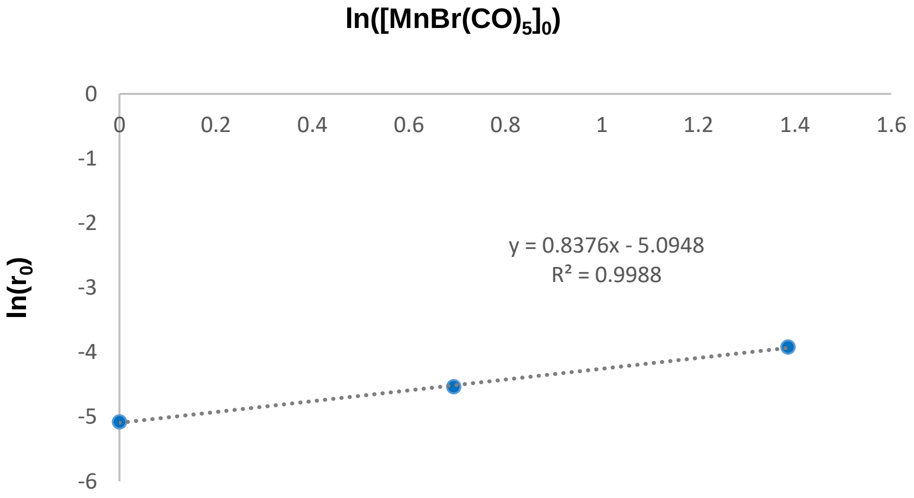

$[MnBr(CO)_5]$ (mM)	r_0 (mM/s)	$\ln([MnBr(CO)_5]_0)$	$\ln(r_0)$
1	0.0062	0	-5.1499
2	0.0107	0.693147181	-4.48295
4	0.0198	1.386294361	-3.88733

Figure S13: Method used to determine the order of $[MnBr(CO)_5]$ in the initial reaction rate using the initial rates method.

8.3. Order in $PhMe_2SiH$

Table S10: Concentration values for each component used to determine the order of $PhMe_2SiH$ in the reaction rate.

Run	$[PhMe_2SiH]$	$[H_2O]$	$[MnBr(CO)_5]$
2*	100 mM	300 mM	2 mM
4	25 mM	300 mM	2 mM
5	50 mM	300 mM	2 mM
6*	100 mM	150 mM	2 mM
8	25 mM	150 mM	2 mM
9	50 mM	150 mM	2 mM

*Repeated two times

The concentration plots obtained by NMR (Figure S14) show the concentration of $PhMe_2SiOH$ with time scales normalized to a zeroth- (top left), first- (top right), second- (bottom left) and 0.85^{th}- (bottom right) order dependence on the concentration of $PhMe_2SiH$. According to the variable time normalization analysis, the best fit of the time course for the formation of dimethylphenylsilanol was obtained for an order of 0.85 (bottom right graph, Figure S14). The data were also analyzed with the initial rates method (Figure S15). The initial reaction rate (r_0) was determined as the average of two sets of experiments using concentrations of H_2O of 300 mM (r_{01}) and 150 mM (r_{02}), as it is observed in the table of Figure S15. An initial reaction order of 0.79 with respect to the $PhMe_2SiH$ was obtained as the slope of the natural logarithm of the average of the initial reaction rate of each run over the natural logarithm of the concentration of $PhMe_2SiH$ of the corresponding run.

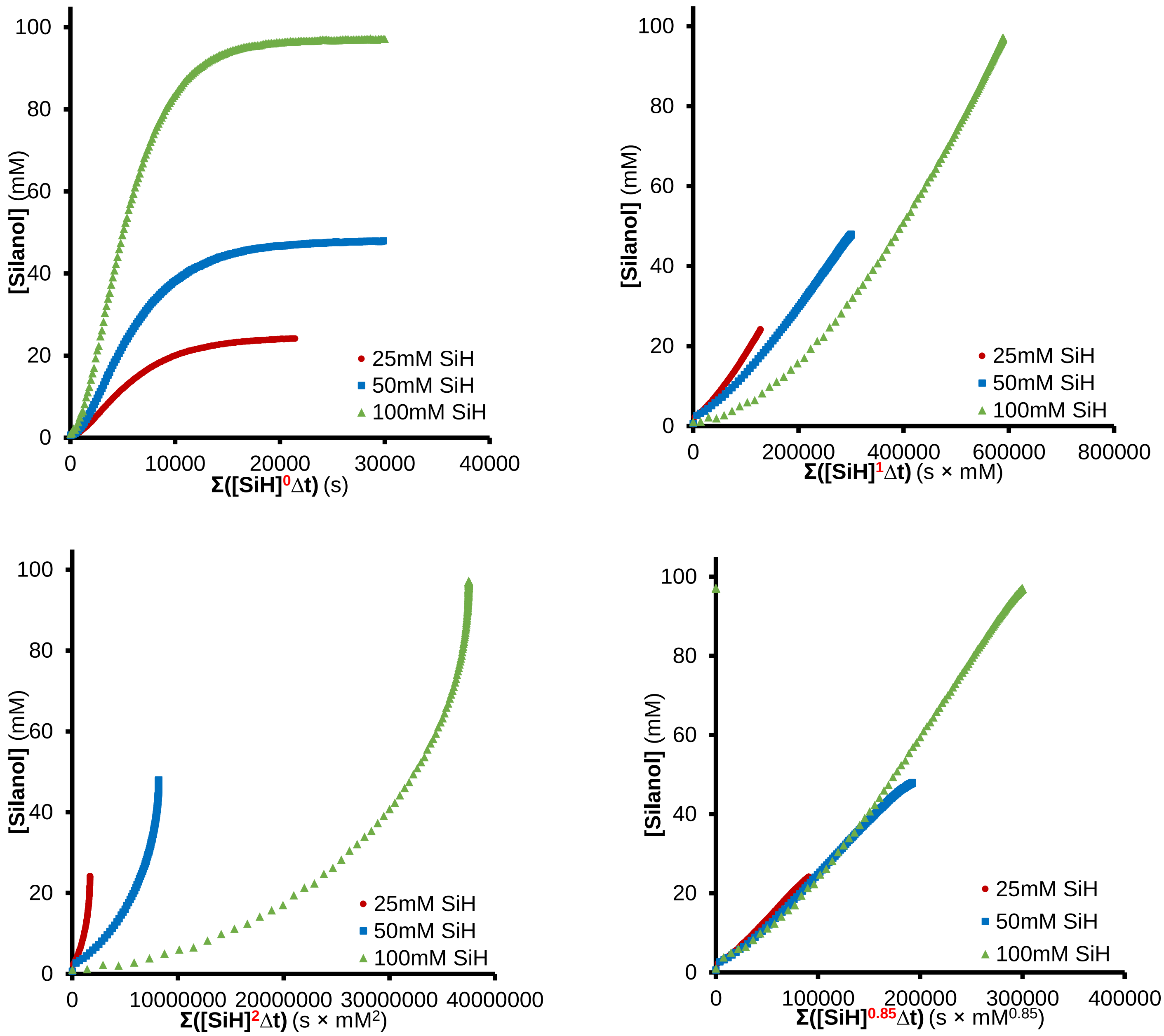

Figure S14: Plots obtained via VTNA of the NMR data where the concentration of $PhMe_2SiH$ is elevated to different exponents as described in the above paragraph.

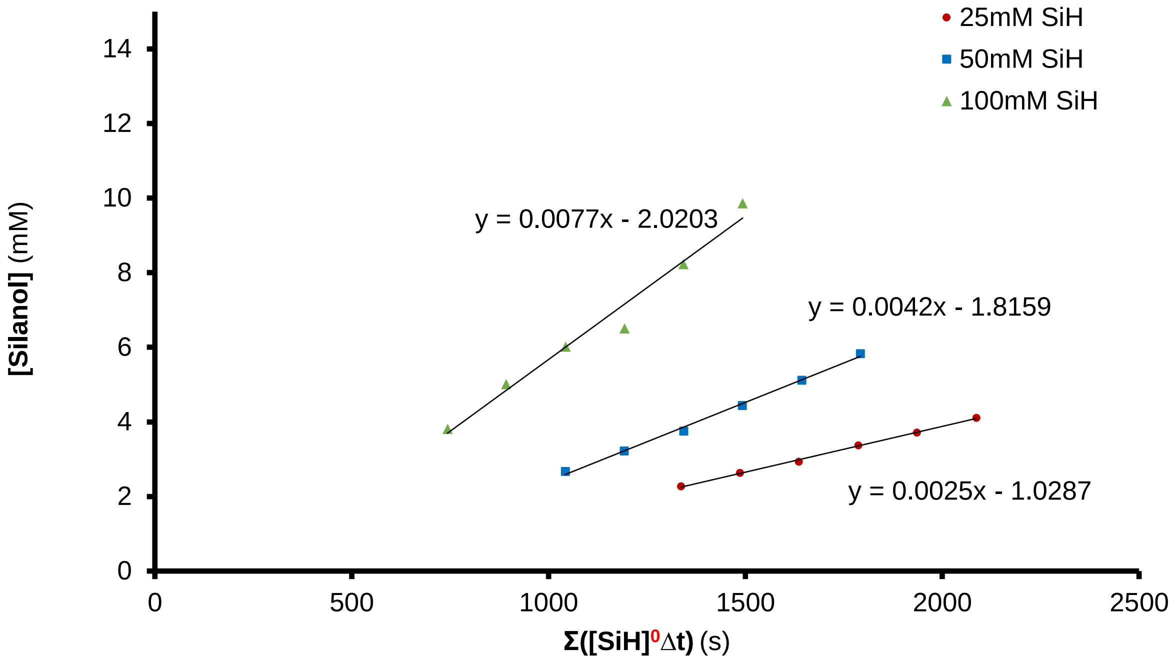

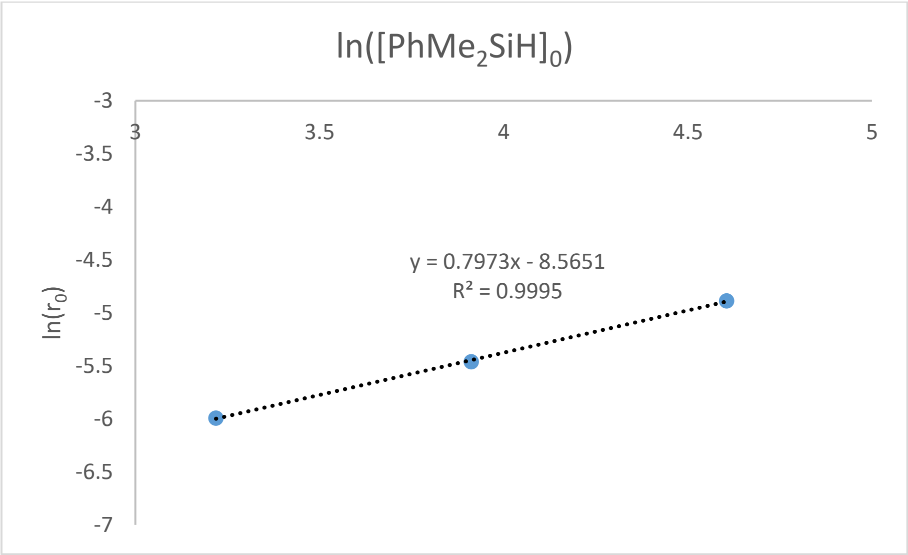

$[PhMe_2SiH]_0$	r_{01} (mM/s)	r_{02} (mM/s)	r_0 (mM/s)	$\ln([PhMe_2SiH]_0)$	$\ln(r_0)$
100	0.0077	0.0074	0.00755	4.60517	-4.88621
50	0.0042	0.0043	0.00425	3.912023	-5.46084
25	0.0025	0.0025	0.0025	3.218876	-5.99146

Figure S15: Method used to determine the order of $PhMe_2SiH$ in the initial reaction rate using the initial rate**s** method.

8.4. Order in H_2O

Table S11: Concentration values for each component used to determine the order of H_2O in the reaction rate.

Run	[$PhMe_2SiH$]	[H_2O]	[$MnBr(CO)_5$]
2*	100 mM	300 mM	2 mM
6*	100 mM	150 mM	2 mM
7	100 mM	600 mM	2 mM

*Repeated two times

The concentration plots obtained by NMR (Figure S16) show the concentration of $PhMe_2SiOH$ with time scales normalized to a zeroth- (top left), first- (top right), second- (bottom left) and -0.6th- (bottom right) order dependence on the concentration of H_2O. According to the variable time normalization analysis, the best fit of the time course for the formation of dimethylphenylsilanol was obtained for an order of -0.6 (bottom right graph, Figure S16). The data were also analyzed with the initial rates method (Figure S17). The initial reaction rate (r_0) for each run was determined as the slope of the concentration of silanol over time. An initial reaction order of -0.71 with respect to H_2O was obtained as the slope of the natural logarithm of the initial reaction rate of each run over the natural logarithm of the concentration of H_2O of the corresponding run.

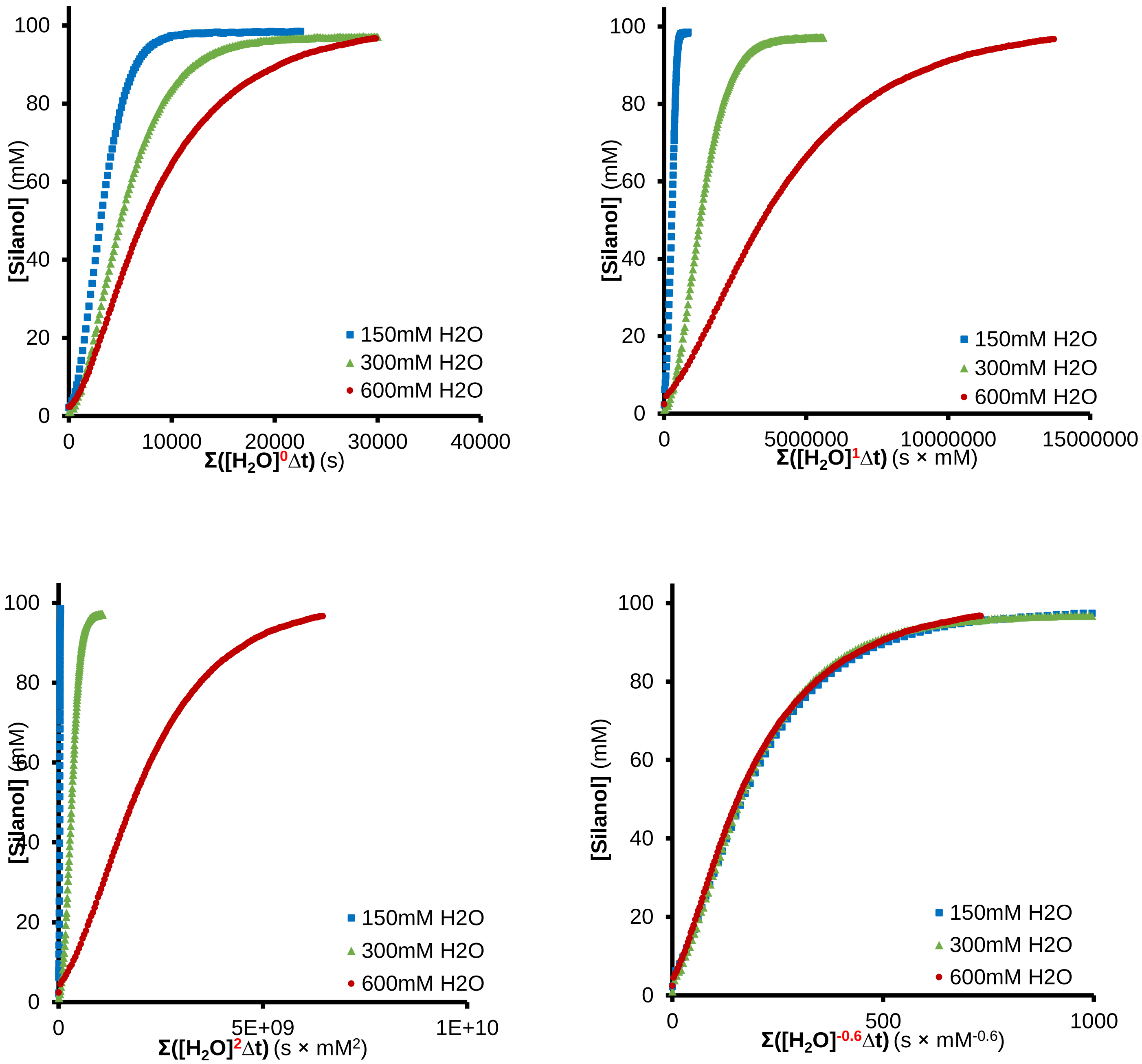

Figure S16: Plots obtained via VTNA of the NMR data where the concentration of H_2O is elevated to different exponents, as described in the above paragraph.

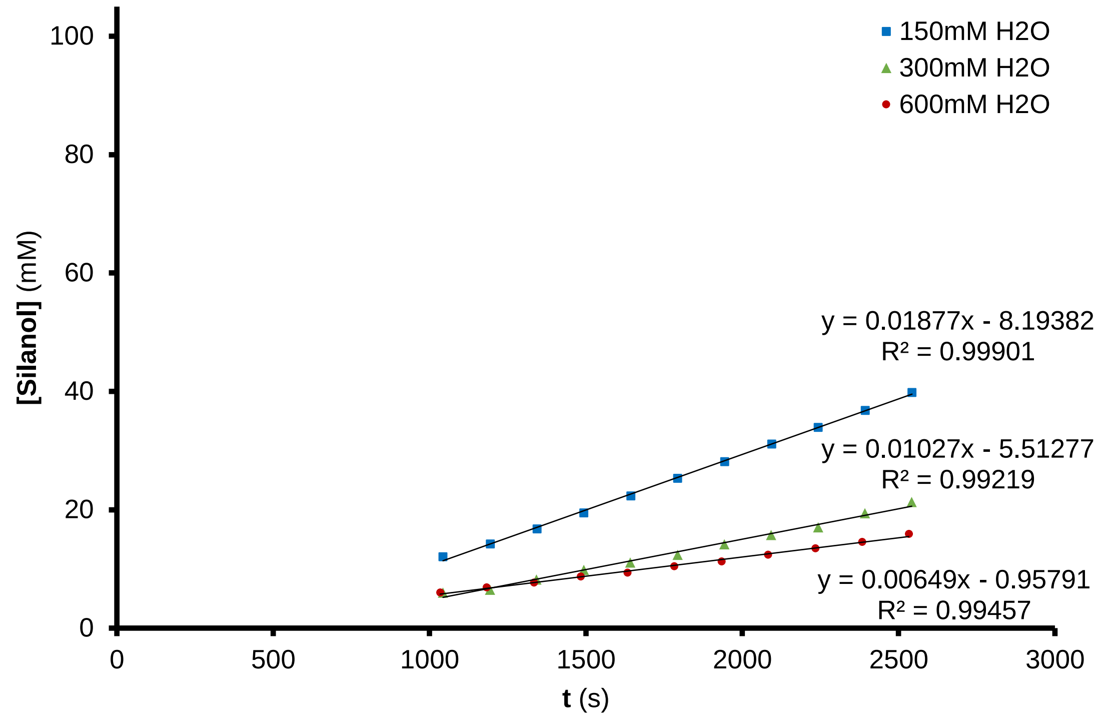

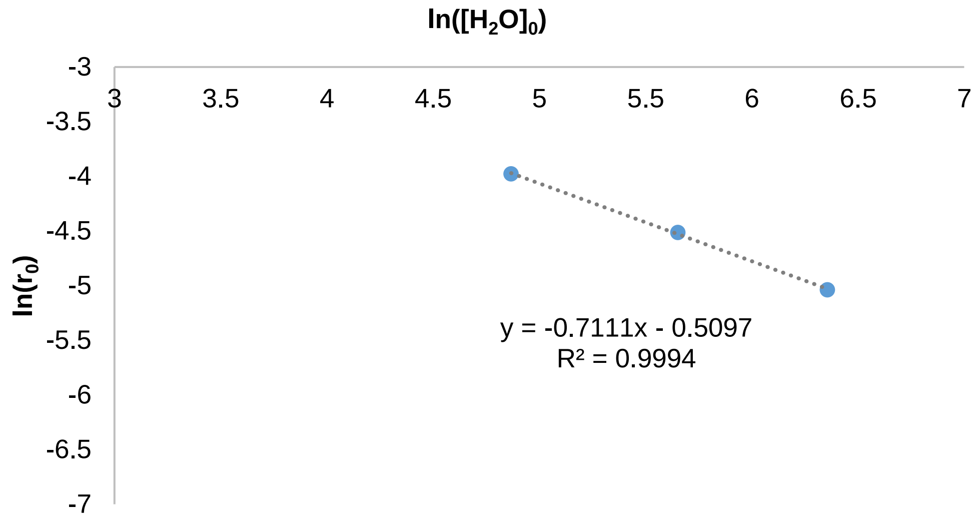

**$[H_2O]_0$** (mM)	**r_0** (mM/s)	**$\ln([H_2O]_0)$**	**$\ln(r_0)$**
130	0.01872	4.867534	-3.97816
285	0.01096	5.652489	-4.5135
576	0.00649	6.356108	-5.03749

Figure S17: Method used to determine the order of H_2O in the initial reaction rate using the initial rates method.

8.5. Kinetic Isotope Effect of $PhMe_2SiD$

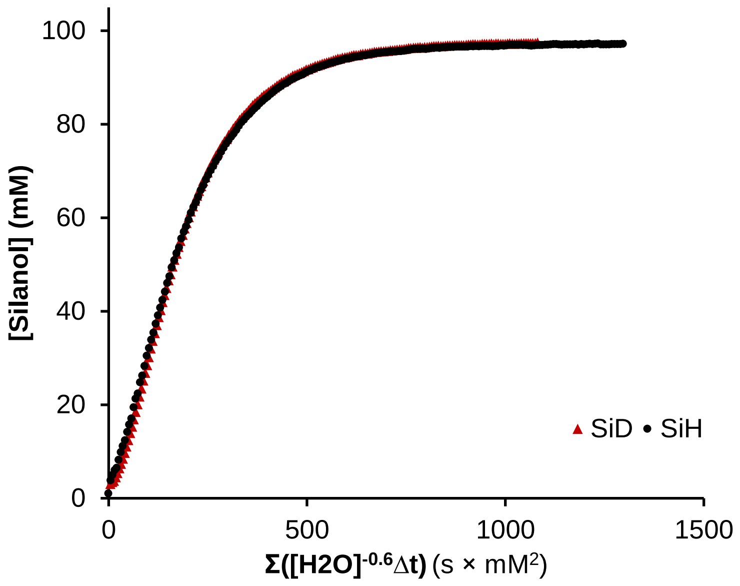

Graph S1: Concentration profiles of $PhMe_2SiOH$ with time scale normalized with respect to the water content. The starting concentration for each component is given in Table S8: Run 2 (in black) and Run 10 (in red).

8.6. Kinetic Isotope Effect of D_2O

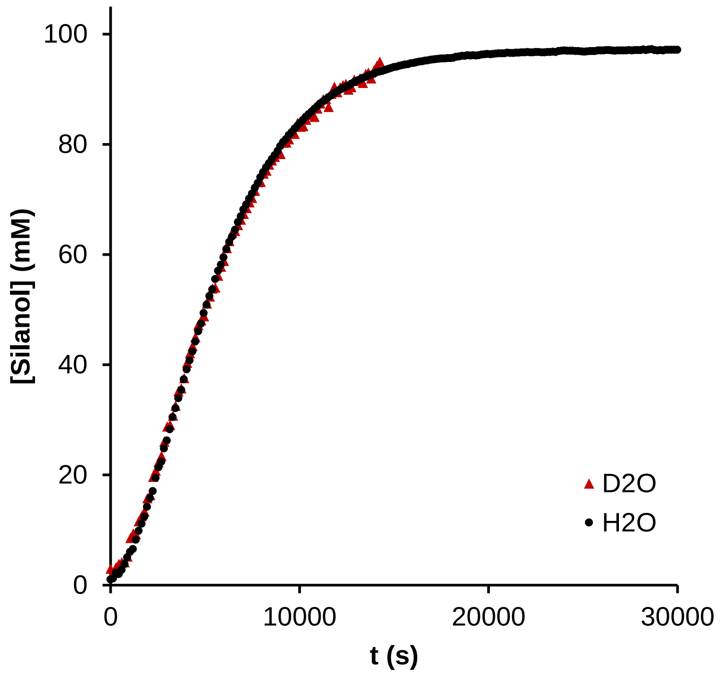

Graph S2: Concentration profiles of $PhMe_2SiOH$ over time. The starting concentration for each component are given in Table S8: Run 2 (in black) and Run 11 (in red).

8.7. Product inhibition

Following the general procedure reported in Section 8.1, the kinetic run number 2 (Table S8) was repeated and followed after adding 25 mol% and 50 mol% of $PhMe_2SiOH$ with respect to the initial concentration of $PhMe_2SiH$. All the concentration values are reported in Table S12. The results of the experiment are reported in Graph S3

Table S12: Concentration values for each component used to determine the presence of product inhibition in the reaction.

Run	[$PhMe_2SiH$]	[H_2O]	[$MnBr(CO)_5$]	[$PhMe_2SiOH$]
2*	100 mM	300 mM	2 mM	0 mM
12	100 mM	300 mM	2 mM	25 mM
13	100 mM	300 mM	2 mM	50 mM

*Repeated two times

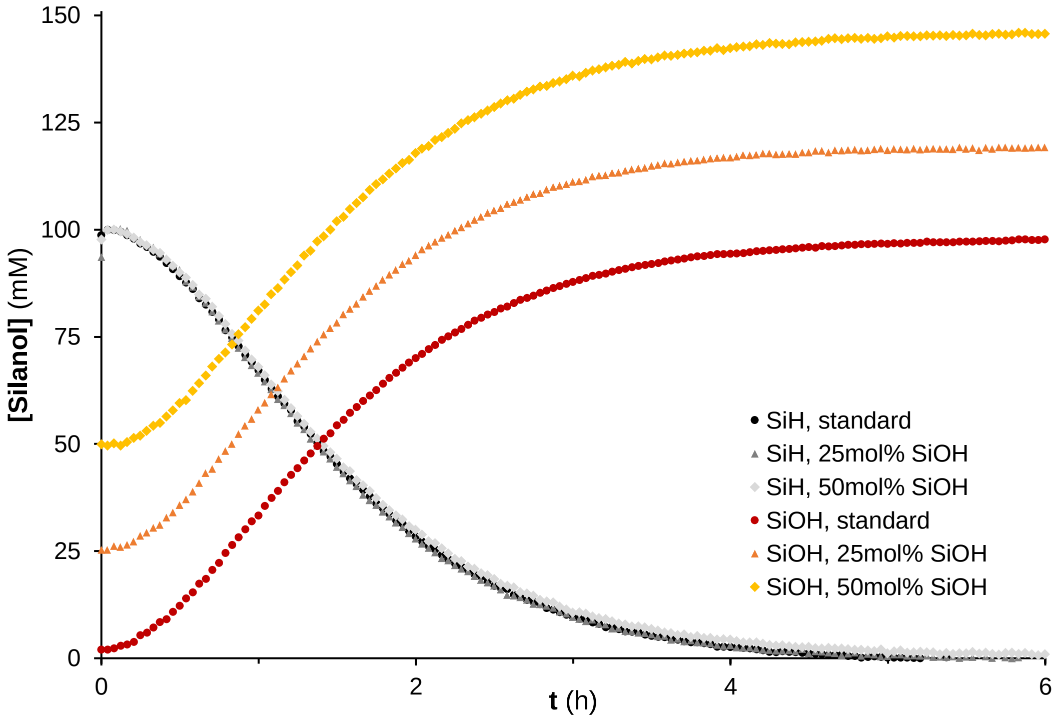

Graph S3: Concentration of $PhMe_2SiOH$ and $PhMe_2SiH$ over time for runs 2, 12, and 13.

8.8. Experiment with enriched [MnBr(^{13}CO)$_5$]

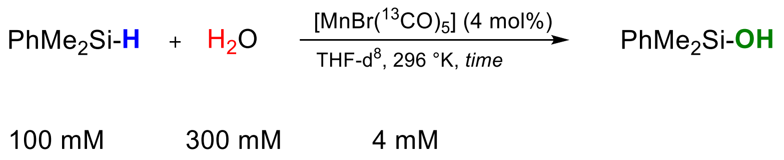

Scheme S4: Reaction performed according to the procedure reported in section 8.1 using ^{13}C-enriched [MnBr(CO)$_5$]. L = THF, PhMe$_2$SiOH, H_2O, CO, PhMe$_2$SiH.

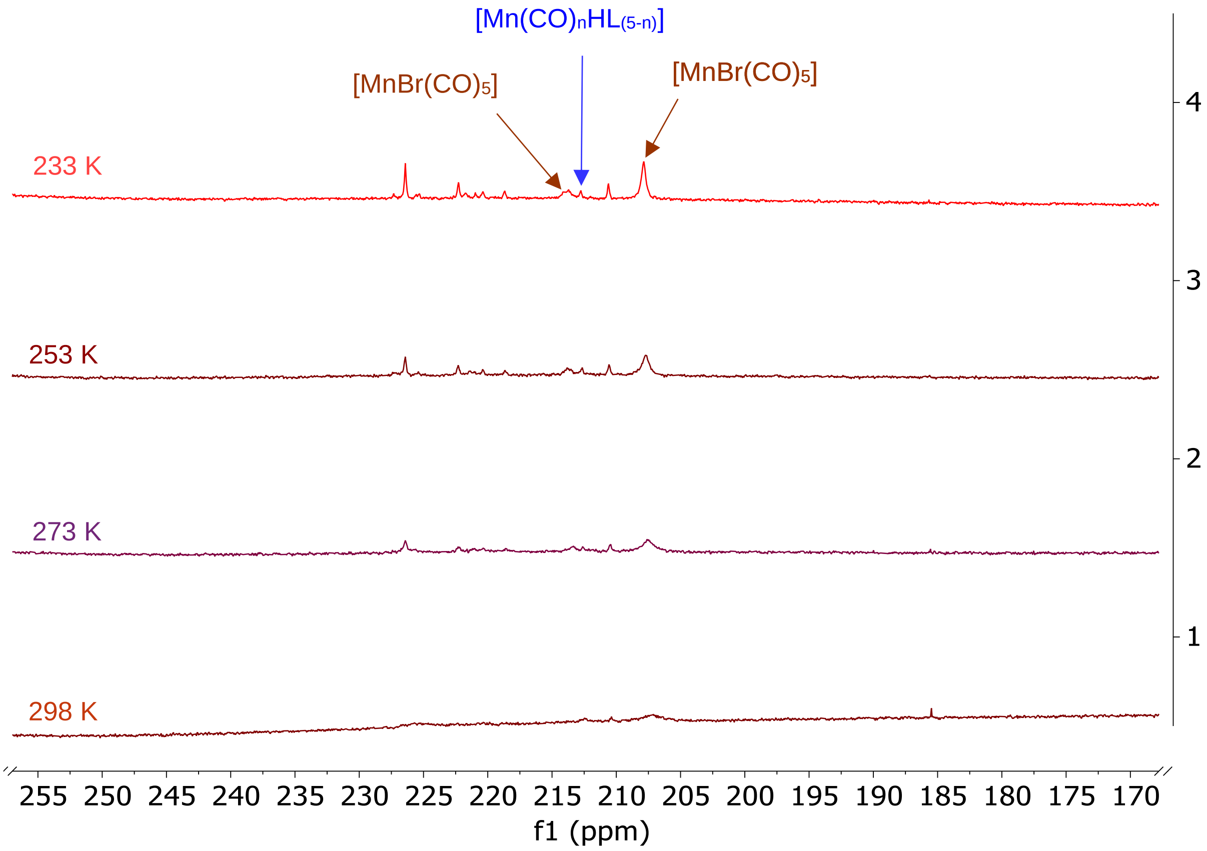

Figure S18: ^{13}C NMR (151 MHz, THF-d_8, various T) spectra of the reaction reported in Scheme S4 at different temperatures.

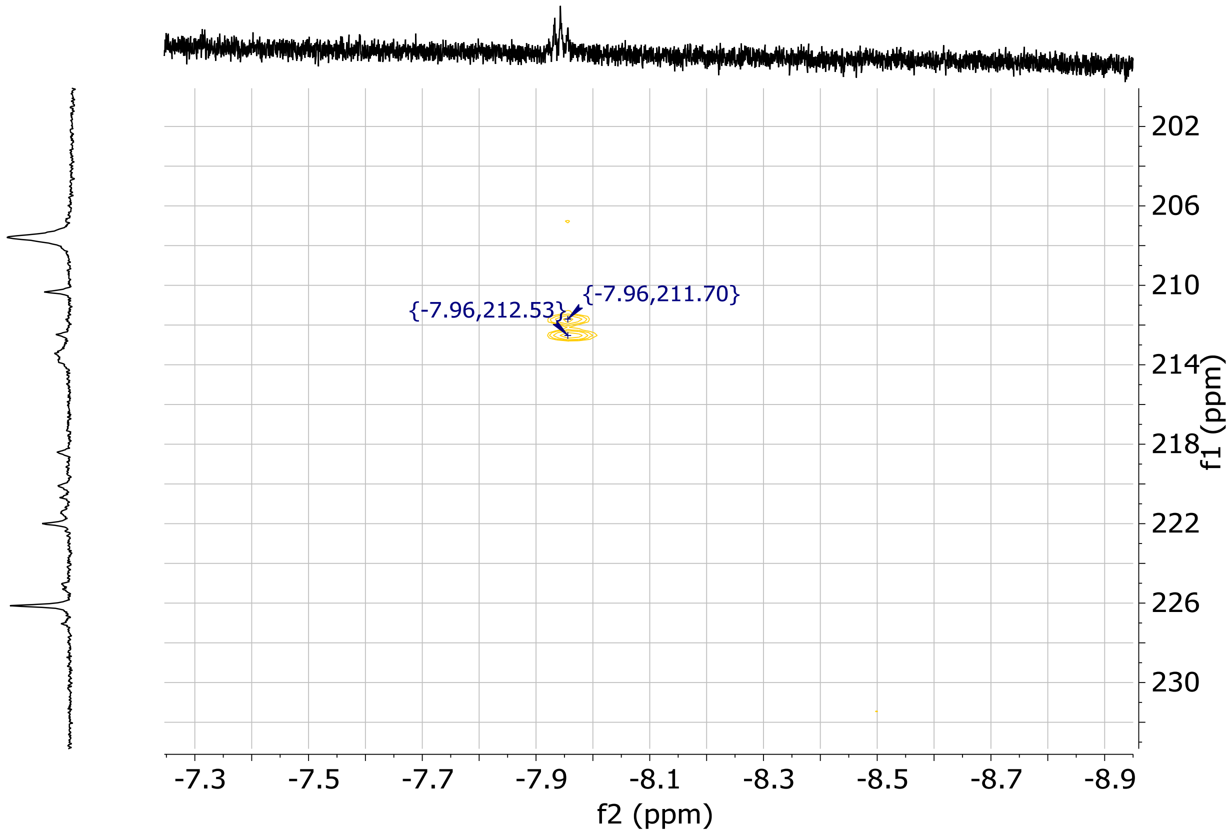

Figure S19: HMBC NMR (600 MHz, THF-d_8, 233 K) detail of the spectrum showing the presence of a [Mn-H] complex.

9. Gas-phase Analysis Studies

9.1. Study on [MnBr(CO)$_5$]

[Mn(CO)$_5$Br] (5.5 mg, 0.02 mmol) and dimethylphenylsilane (133.8 mg, 1.0 mmol) were weighed in screw cap Schlenk tube inside the glovebox. The reaction vessel was taken outside the glovebox and covered with aluminum foil. H_2O (36 µL, 2.0 mmol) and THF (1 mL) were added to the vial under argon flush. The reaction mixture was heated in an oil bath set to 50 °C for 1 hour. Subsequently, the reaction vessel was allowed to cool down to RT, and 100 µL of the gas phase was injected into the GC-TCD. The quantification of $H_{2(g)}$ and $CO_{(g)}$ has been performed using calibration curves corresponding to the respective integrated areas of the two gases.

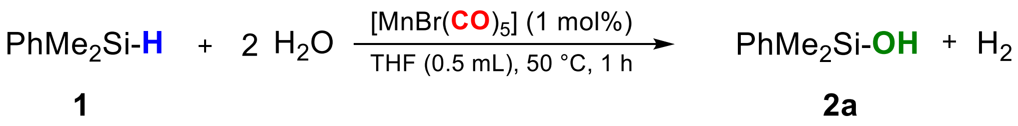

Scheme S5: Reaction between PhMe$_2$SiH and H_2O in the presence of 1 mol% [MnBr(CO)$_5$].

Gas identified	Retention time	Vol%
Hydrogen	2.217	18.014
Argon	3.270	9.816
Nitrogen	3.418	71.438
CO	4.173	0.725

Ratio H_2:CO = 25

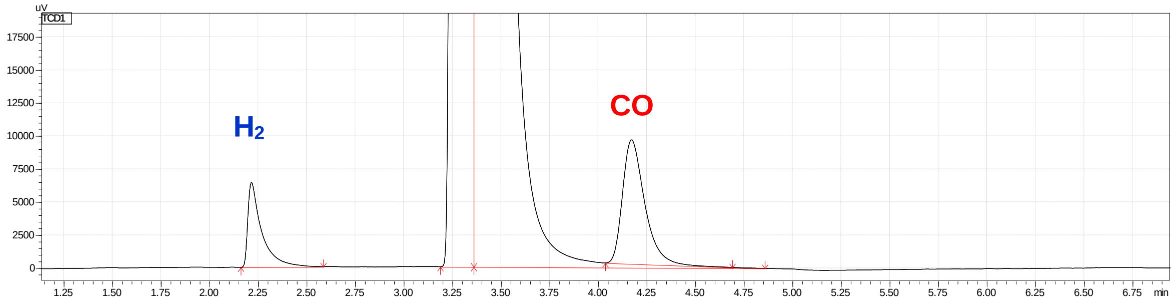

9.2. Study on Mn-4

Mn-4 (3.1 mg, 0.005 mmol) and dimethylphenylsilane (76.6 mg, 0.5 mmol) were weighed in a screw cap Schlenk tube inside the glovebox. The reaction vessel was taken outside the glovebox. H_2O (18 µL, 1.0 mmol) and 2-MTHF (0.5 mL) were added to the vial under argon flush. The reaction mixture was heated in an oil bath set to 80 °C for 1 hour. Subsequently, the reaction vessel was allowed to cool down to RT, and 100 µL of the gas phase was injected into the GC-TCD. No CO or H_2 was detected.

.

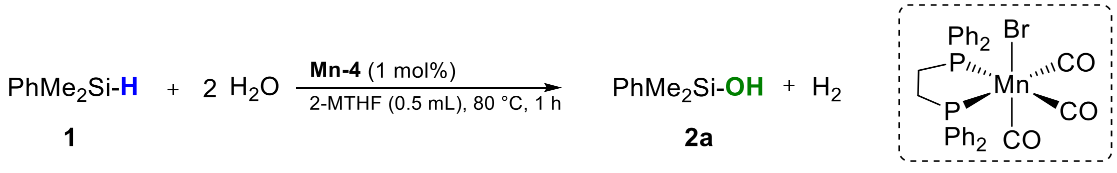

Scheme S6: Reaction between $PhMe_2SiH$ and Mn-4

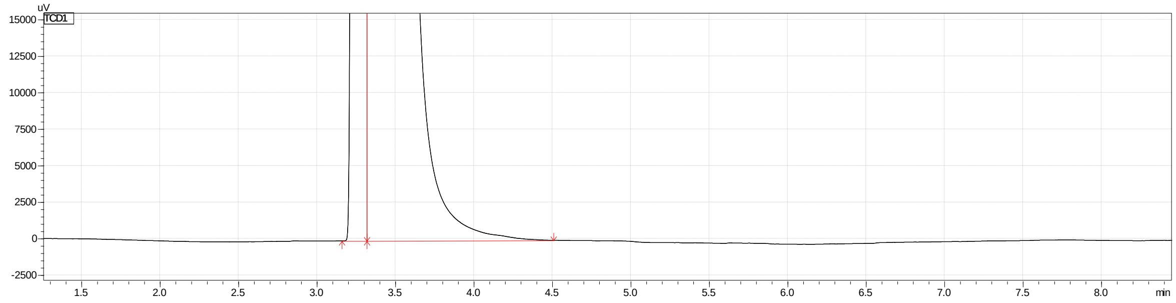

No H_2 or CO detected

9.3. Study on the activation of [MnBr(CO)5], the precatalyst

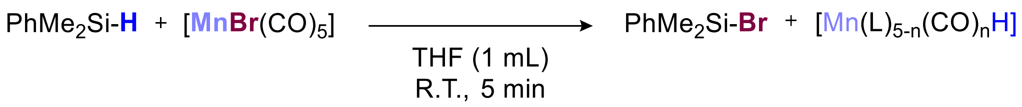

Scheme S7: Reaction between $PhMe_2SiH$ and $[MnBr(CO)_5]$ 1:1.

$[Mn(CO)_5Br]$ (28.5 mg, 0.1 mmol) and dimethylphenylsilane (15.7 mg, 0.1 mmol) were dissolved in THF (1 mL) in screw cap 4 mL vial inside the glovebox. The vial was closed with a septum screw cap, taken outside the glovebox, and stirred at RT for 5 minutes. Subsequently, 100 µL of the gas phase was injected into the GC-TCD. The quantification of H_2(g) and CO(g) has been performed using calibration curves corresponding to the respective integrated areas of the two gases.

Gas identified	Retention time	Vol%
Hydrogen	2.234	2.584
Argon	3.215	96.581
CO	4.185	0.834

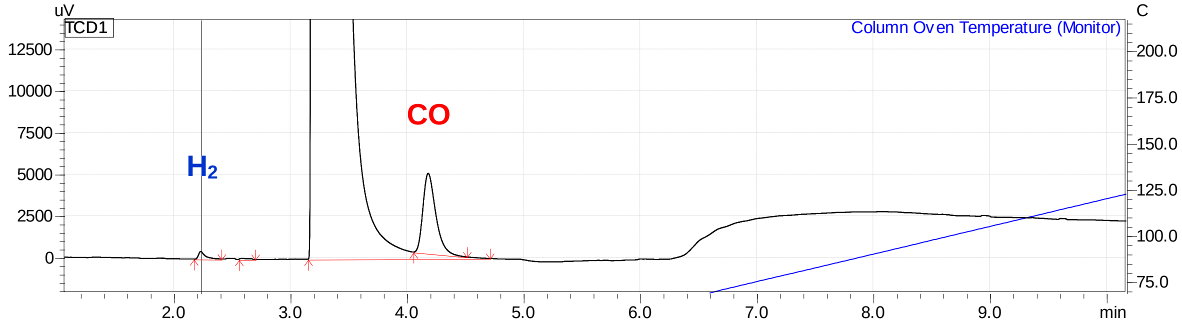

10. Mechanistic studies

10.1. General procedure for the IR experimentation

$$PhMe_2Si\text{-}H \;+\; H_2O \xrightarrow[\text{THF (10 mL), 35 °C, } time]{[MnBr(CO)_5]\ (2\ mol\%)} PhMe_2Si\text{-}OH \;+\; H_2 \;+\; (PhMe_2Si)_2O$$

$[Mn(CO)_5Br]$ (5.5 mg, 0.02 mmol) and Dimethylphenylsilane (136.3 mg, 1.0 mmol) were weighed in 2 separate 4 mL vials inside the glovebox, dissolved in THF and transferred to a volumetric flask of 10 mL that was brought to volume with THF. The solution was placed in a syringe that was brought outside the glovebox and added to a Schlenk tube previously evacuated and filled with argon 3 times containing 54 µL of H_2O (3.0 mmol). The reaction mixture was then transferred with a syringe to a Fischer-Porter bottle under Ar flow, pre-heated by an oil bath to 35 °C, and previously flushed with argon for 5 minutes. The Fischer-Porter bottle was equipped with a stirrer bar and a flow system as described in the General considerations (Section 1). Subsequently, the flow was started with a rate of 3 mL/min, and the FT-IR recorded 1 spectrum/minute.

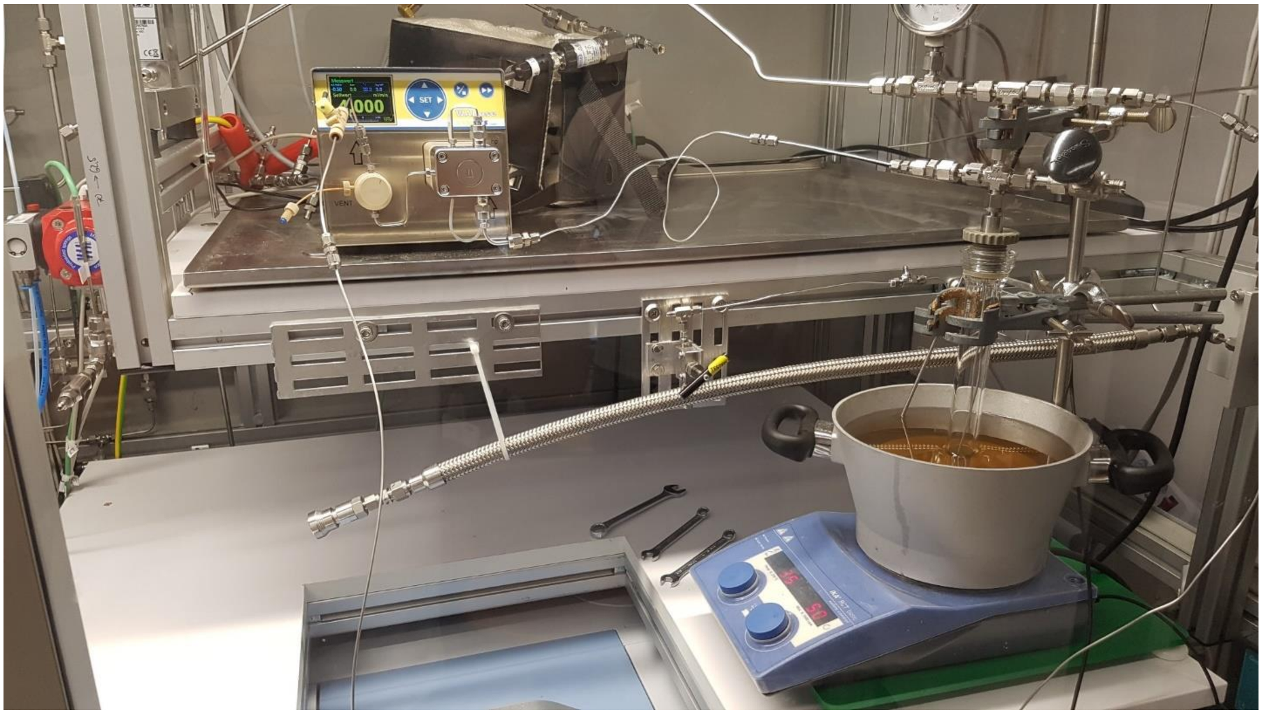

Figure S20: experimental setup used to perform the FT-IR flow analysis.

10.2. FT-IR Spectra of relevant substances in THF

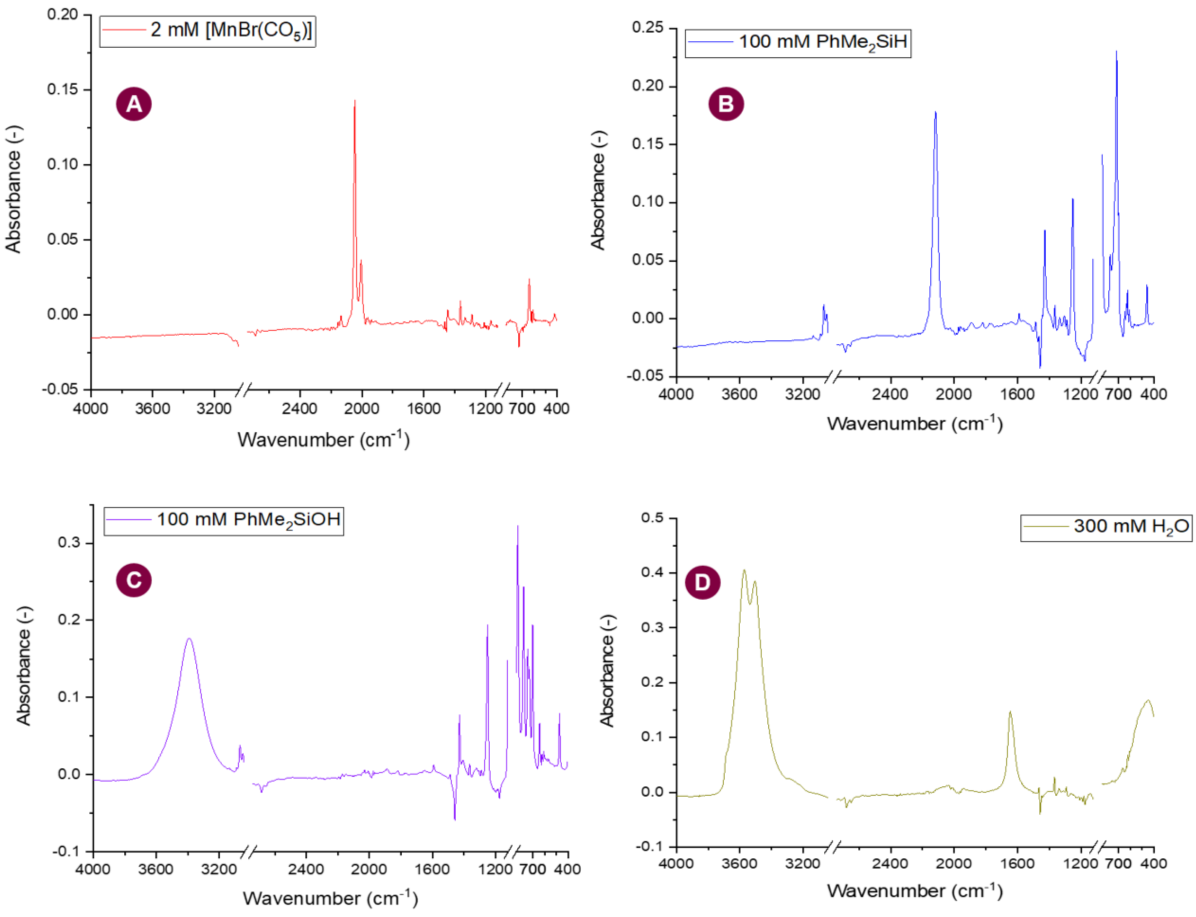

Figure S21: Selected spectra of the reaction components studied in the reaction at the respective concentrations used in the experiment. The areas where the THF-solvent cutoff is present have been removed.

10.3. Key spectra and significant absorbance-time plots

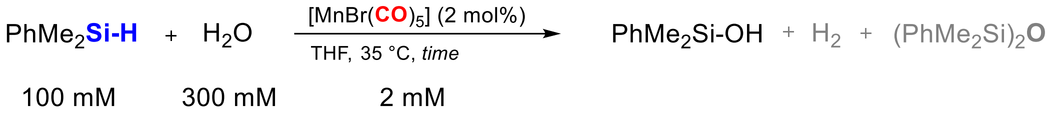

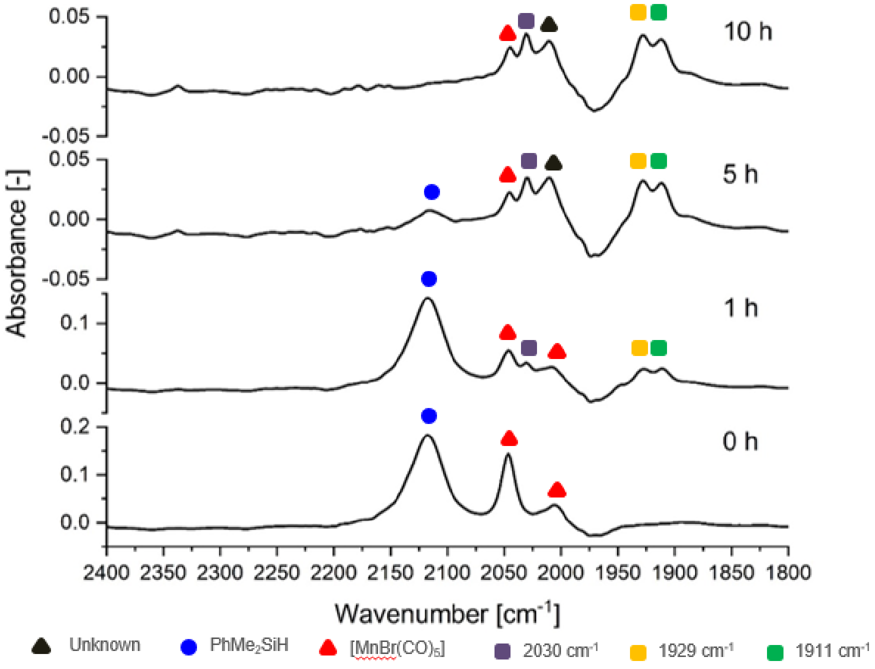

Scheme S8: Selected spectra since the beginning of the reaction above.

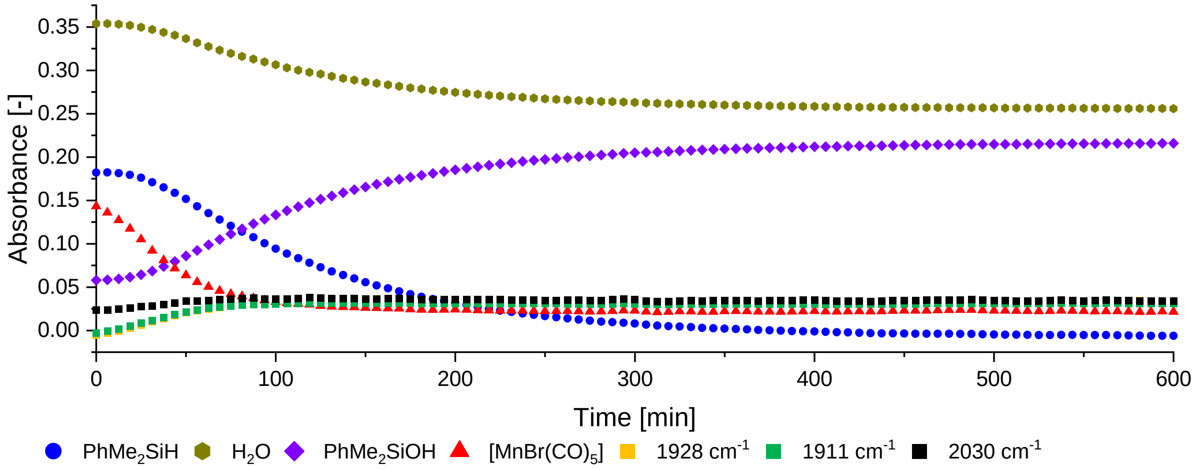

Graph S4: Plot reporting the absorbance of a specific wavelength over time for the reaction reported in Scheme S8.

10.4. Crystallization of Mn-5

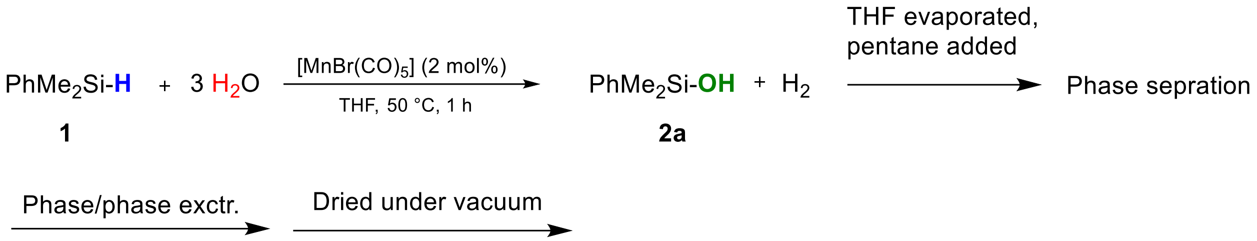

[MnBr(CO)$_5$] (27.0 mg, 0.1 mmol) and PhMe$_2$SiH (689.2 mg, 5.1 mmol) were weighed in a Schlenk tube inside the glovebox. The reaction vessel was taken outside the glovebox. H_2O (270 µL, 15.0 mmol) and THF (5 mL) were added. The reaction mixture was heated in an oil bath set to 50 °C for 1 hour. Subsequently, the reaction vessel was allowed to cool down to RT, and THF was removed *in vacuo*. Pentane (2 mL) was added to the crude reaction mixture leading to phase separation of the residual H_2O (Figure S22, panels A and B). H_2O (1 mL, previously degassed) was added to the Schlenk tube, and the yellow water phase was extracted using pentane (3*2 mL) and once with Et_2O (2 mL, Figure S22, panel C). The water was removed *in vacuo*, leading to a dark orange solid whose IR spectrum is reported in Graph S5. From this orange solid, it was possible to crystallize **Mn-5**.

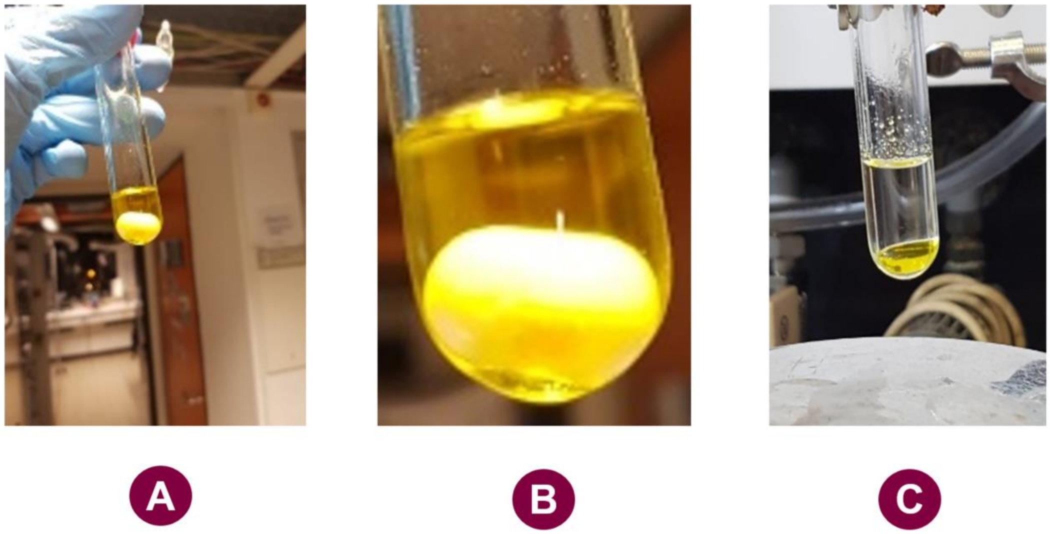

Figure S22: Pictures of the reaction mixture illustrating the separation process.

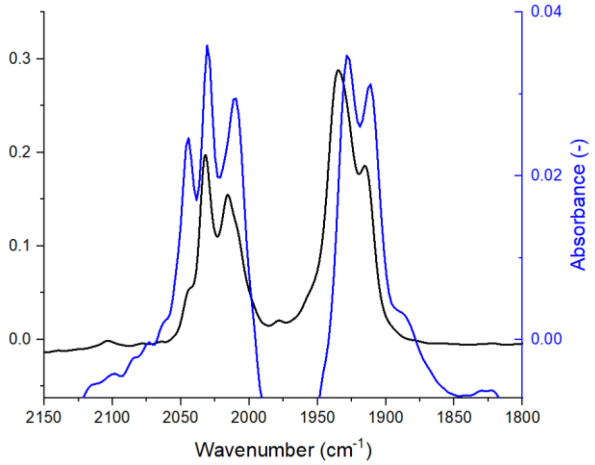

Graph S5: Black: extracted reaction mixture from which **Mn-5** was crystallized. Blue: spectrum after 10 hours from the experiment above.

10.5. Catalytic activity of the solid obtained in Section 10.4

3.8 mg of the solid isolated in section 11.1 (0.0027 mmol)*****, Mesitylene (44.7 mg, 0.37 mmol) and $PhMe_2SiH$ (141.3 mg, 1.0 mmol), were weighed in a 4 mL vial inside the glovebox. The reaction vessel was taken outside the glovebox, placed in a Schlenk tube previously evacuated, and filled with argon 3 times. H_2O (54 µL, 3.0 mmol) and THF (1.0 mL) were added to the vial under argon flush. The vial was placed in a pre-heated oil bath at 50 °C, and the reaction mixture was stirred for 1 h. Subsequently, the reaction vessel was allowed to cool down to RT. An NMR tube was filled with the crude mixture (ca 0.05 mL), DMSO-d_6 (0.5 mL), and ^{1}H NMR spectroscopy was conducted to determine the conversion and yield of the reaction. The yield of 61% was obtained by setting the integral value for the mesitylene singlet (6.76 ppm, 3H) as 1.00 and integrating the characteristic Si-OH signal of the product.

* Calculated assuming that the solid is entirely composed of **Mn-5** and dividing 3.8 mg by the F.W. of **Mn-5**.

11. TON and TOF studies

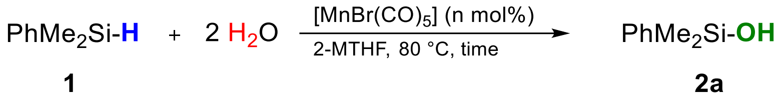

Dimethylphenylsilane (67.1 mg, 0.5 mmol) and mesitylene (20 µL, 0.14 mmol) were weighed in a 4 mL vial inside the glovebox equipped with a screw cap and a magnetic stirrer. [Mn(CO)$_5$Br] (2.8 mg, 0.01 mmol) was weighed in a separate vial, dissolved in 2 mL of 2-MTHF, and the solution was transferred to a Schlenk tube. The vial was taken outside the glovebox, placed in a Schlenk tube previously evacuated, and filled with argon 3 times. H_2O (18 µL, 2 mmol) was added to the vial containing PhMe$_2$SiH, and the chosen volume of stock solution of [Mn(CO)$_5$Br] was added to the vial under argon flush. 2-MTHF was added to reach the desired volume, and the reaction mixture was heated in an oil bath set to 80 °C for the chosen time. Subsequently, the reaction vessel was allowed to cool down to RT, roughly 0.05 mL of the crude mixture was placed in an NMR tube, and DMSO-d_6 (0.5 mL) was added. Yields were obtained by setting the integral value for the characteristic signal of the product (Si-OH, 5.88 ppm, 1H) as 1.00 and integrating the corresponding mesitylene singlet (6.76 ppm, 3H).

Volume	Stock Sol. Vol.	Cat. Load.	Yield, %	TON	Time (s)	Time (h)	TOF (h^{-1})
0.5 mL	500 µL	0.5 mol%	92	184	163	0.045	4088
0.25 mL	100 µL	0.1 mol%	12	120	604	0.17	706

12. NMR Spectra

12.1. NMR yields

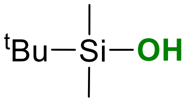

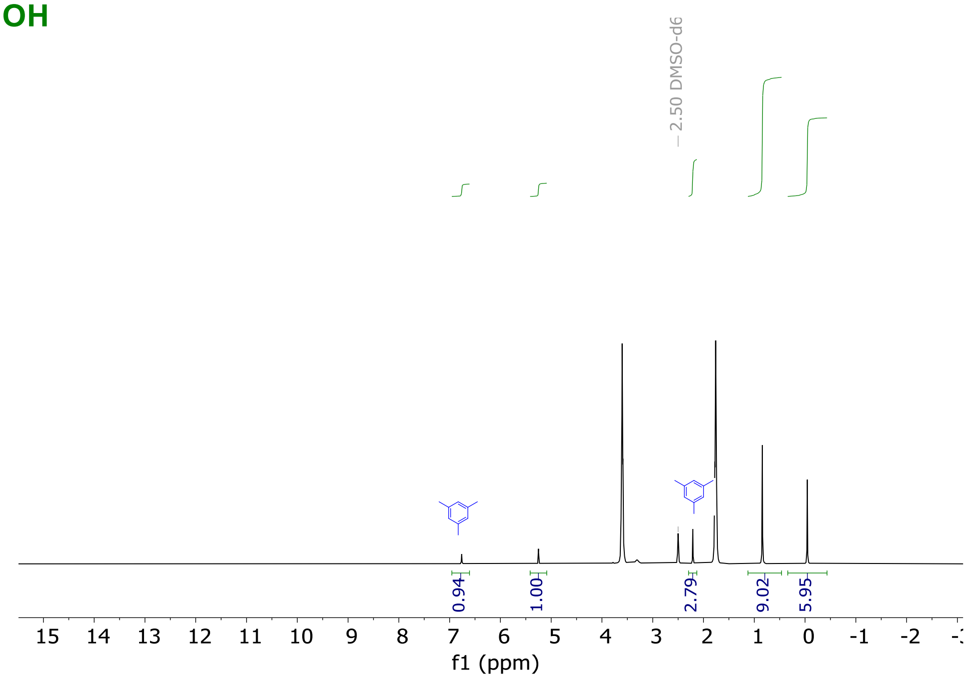

Figure S23: ^{1}H NMR (400 MHz, DMSO-*d*$_6$ 298.1 K) spectrum of the crude reaction mixture yielding compound **2g** according to the procedure in section 3.5.

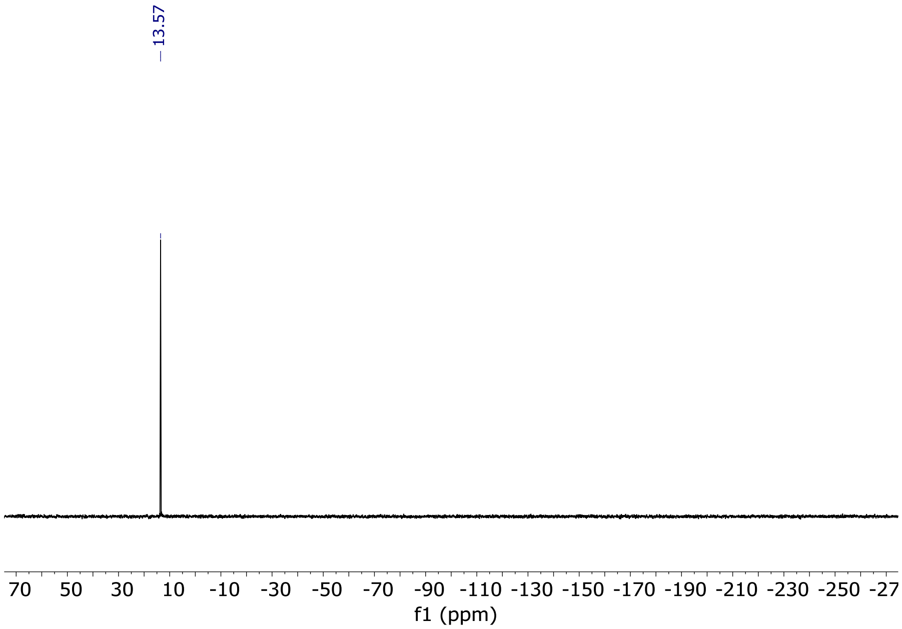

Figure S24: ^{29}Si NMR (79 MHz, DMSO-*d*$_6$, 298.1 K) spectrum of the crude reaction mixture yielding compound **2g** according to the procedure in section 3.5.

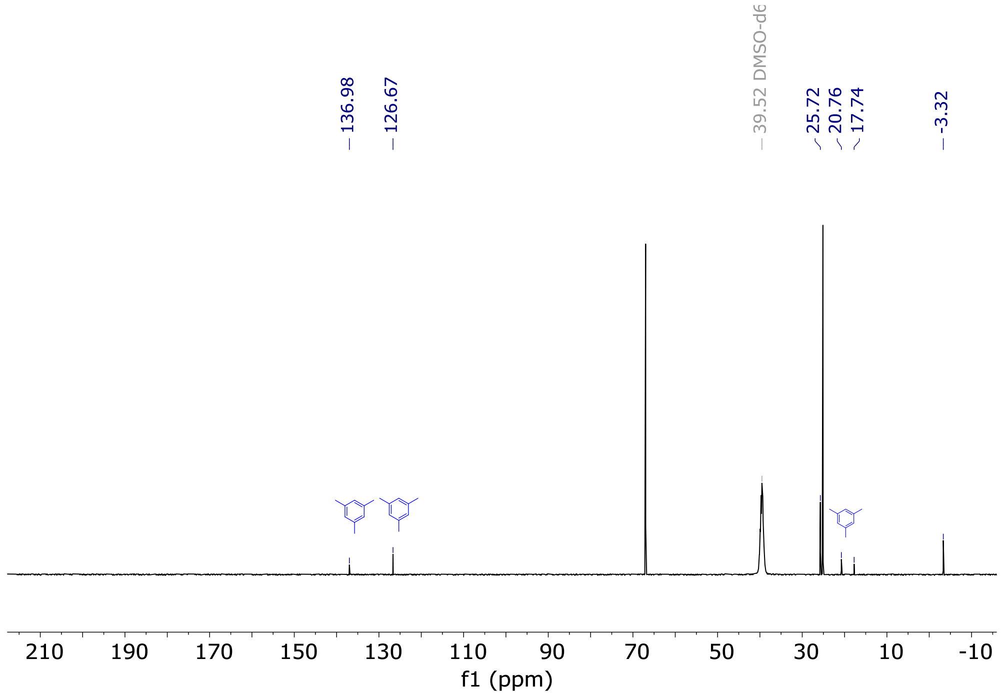

Figure S25: ^{13}C NMR (101 MHz, DMSO-*d*$_6$, 298.5 K) spectrum of the crude reaction mixture yielding compound **2g** according to the procedure in section 3.5.

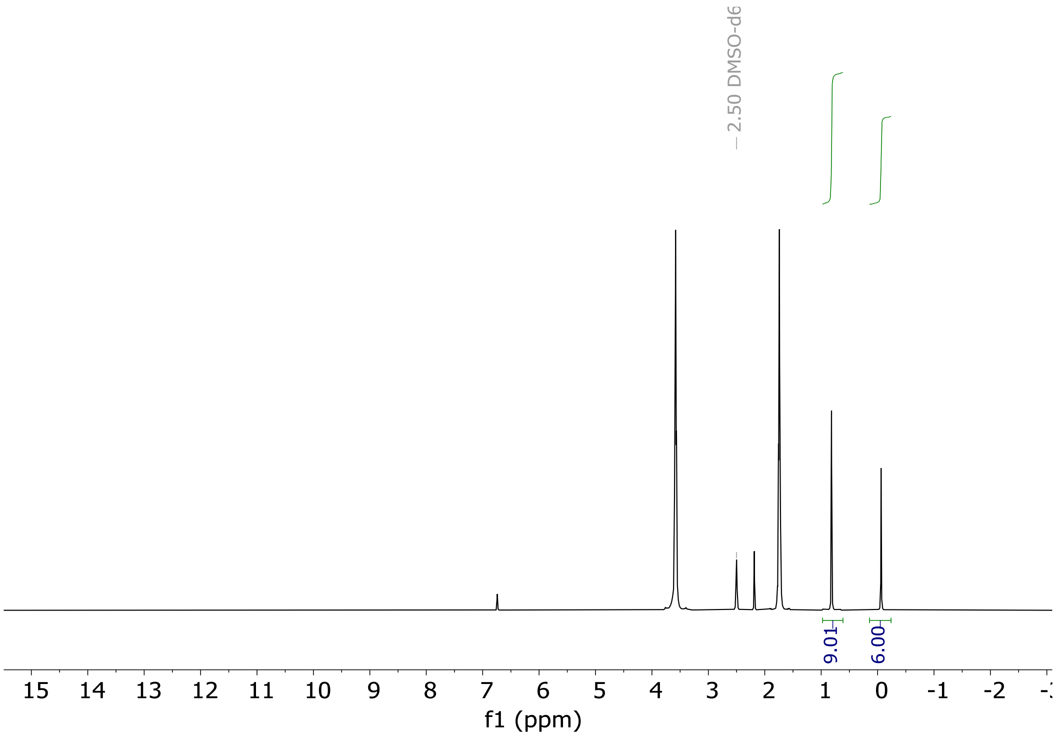

Figure S26: **^{1}H NMR (400 MHz, DMSO-*d*$_6$ 298.1 K)** spectrum of the crude reaction mixture yielding compound **2g** according to the procedure in section 3.5 after adding 0.05 mL of D_2O.

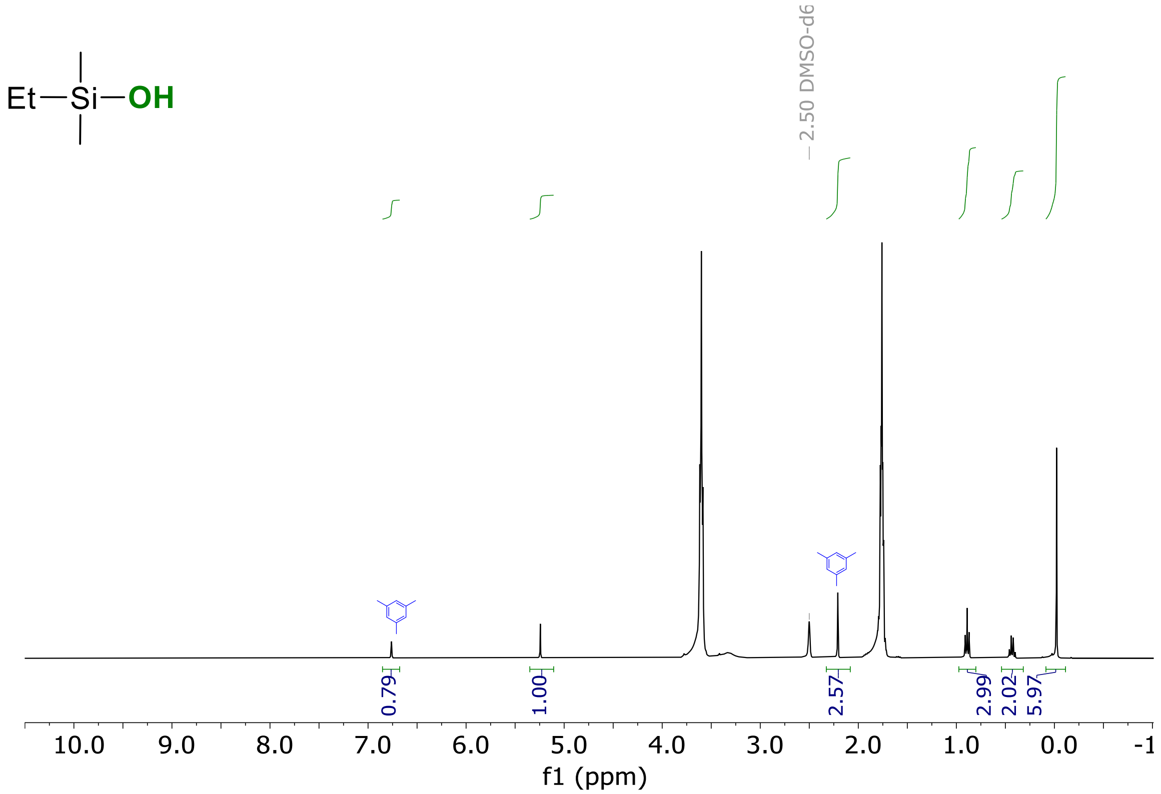

Figure S27: ^{1}H NMR (400 MHz, DMSO-d_6 298.1 K) spectrum of the crude reaction mixture yielding compound **2h** according to the procedure in section 3.5.

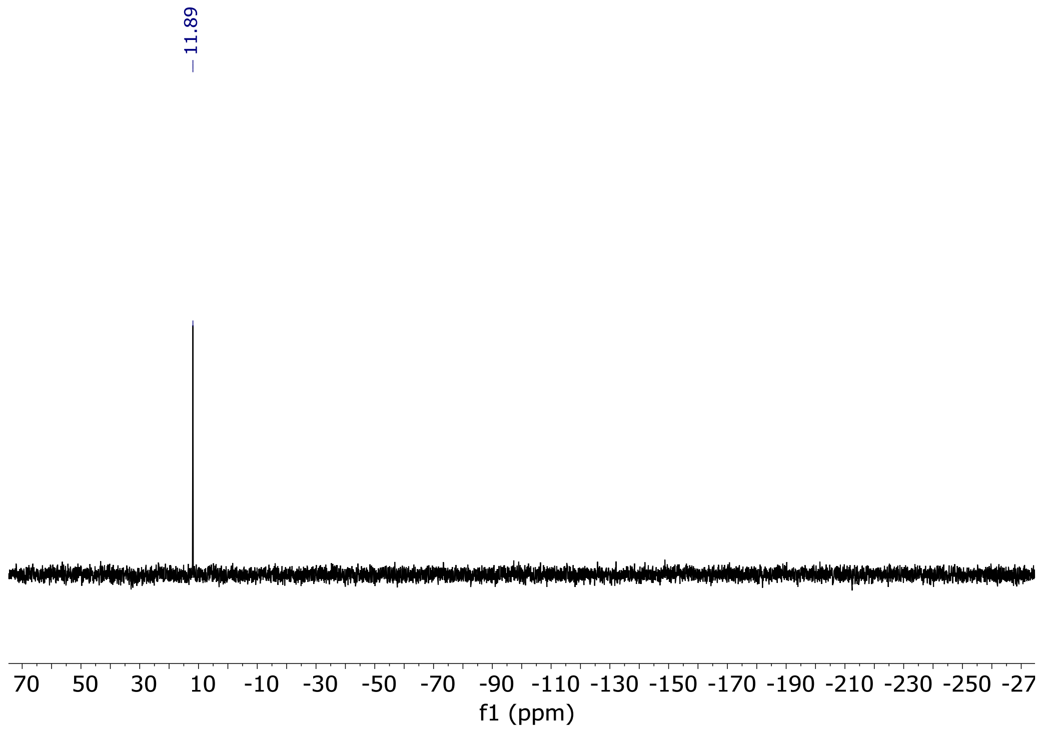

Figure S28: **^{29}Si NMR (79 MHz, DMSO-d_6, 298.1 K)** spectrum of the crude reaction mixture yielding compound **2h** according to the procedure in section 3.5.

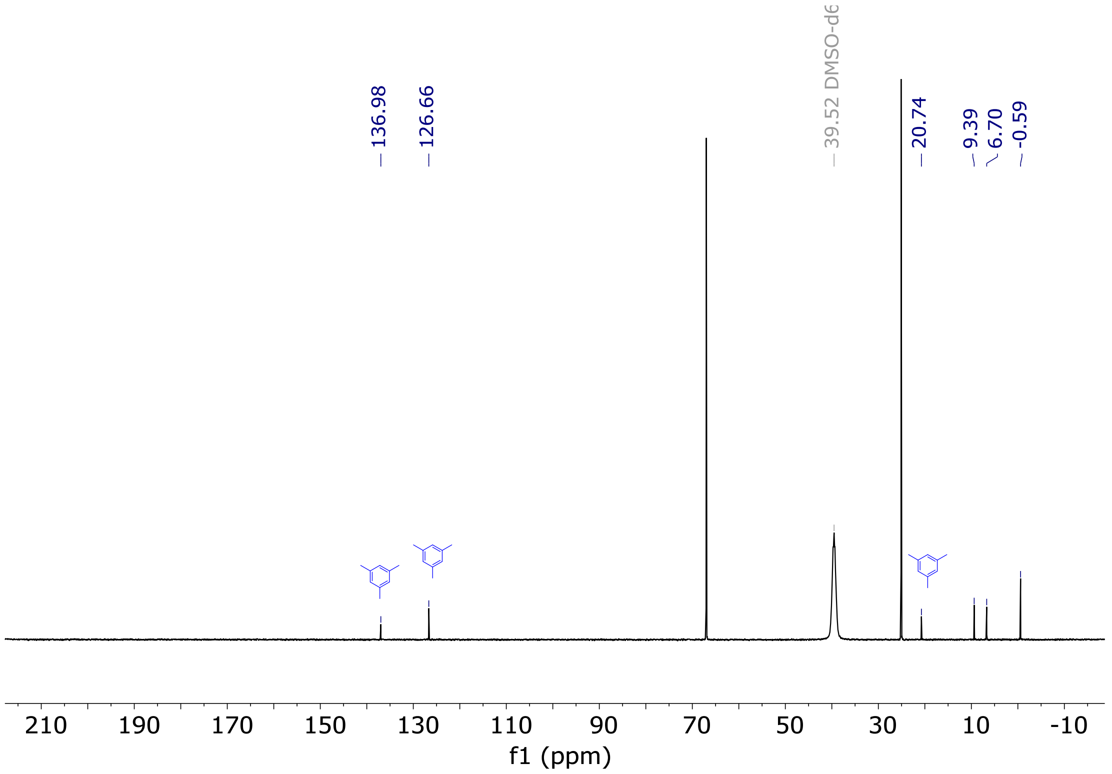

Figure S29: **^{13}C NMR (101 MHz, DMSO-d_6, 298.5 K)** spectrum of the crude reaction mixture yielding compound **2h** according to the procedure in section 3.5.

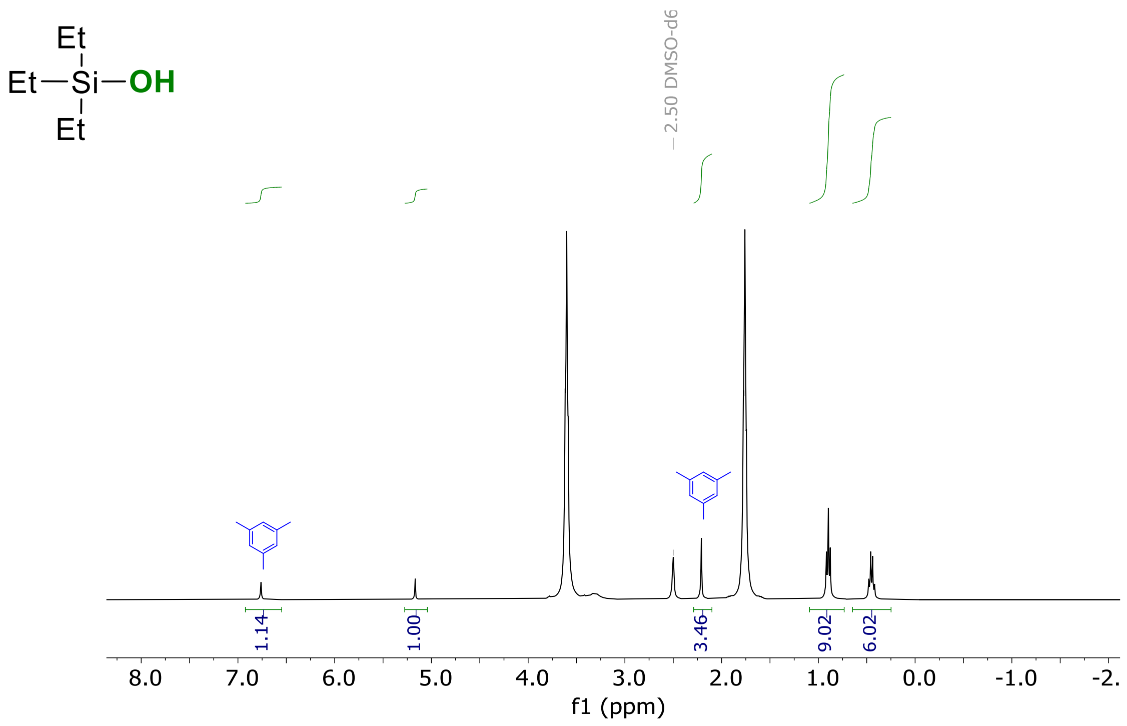

Figure S30: ^{1}H NMR (400 MHz, DMSO-d_6 298.1 K) spectrum of the crude reaction mixture yielding compound **2i** according to the procedure in section 3.5.

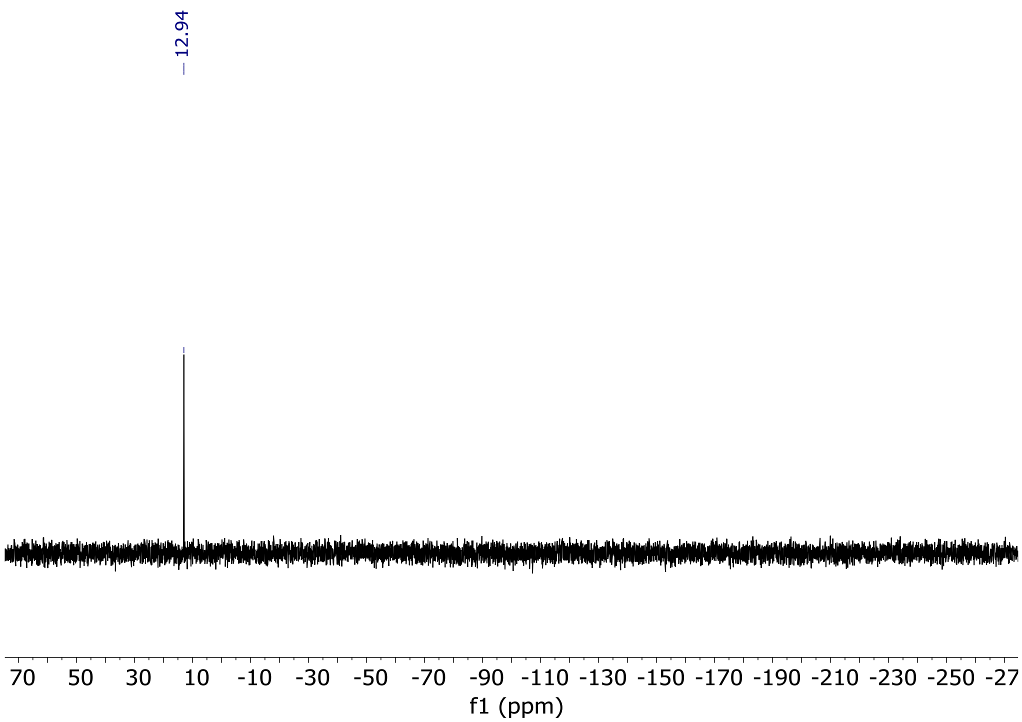

Figure S31: ^{29}Si NMR (79 MHz, DMSO-d_6, 298.2 K) spectrum of the crude reaction mixture yielding compound **2i** according to the procedure in section 3.5.

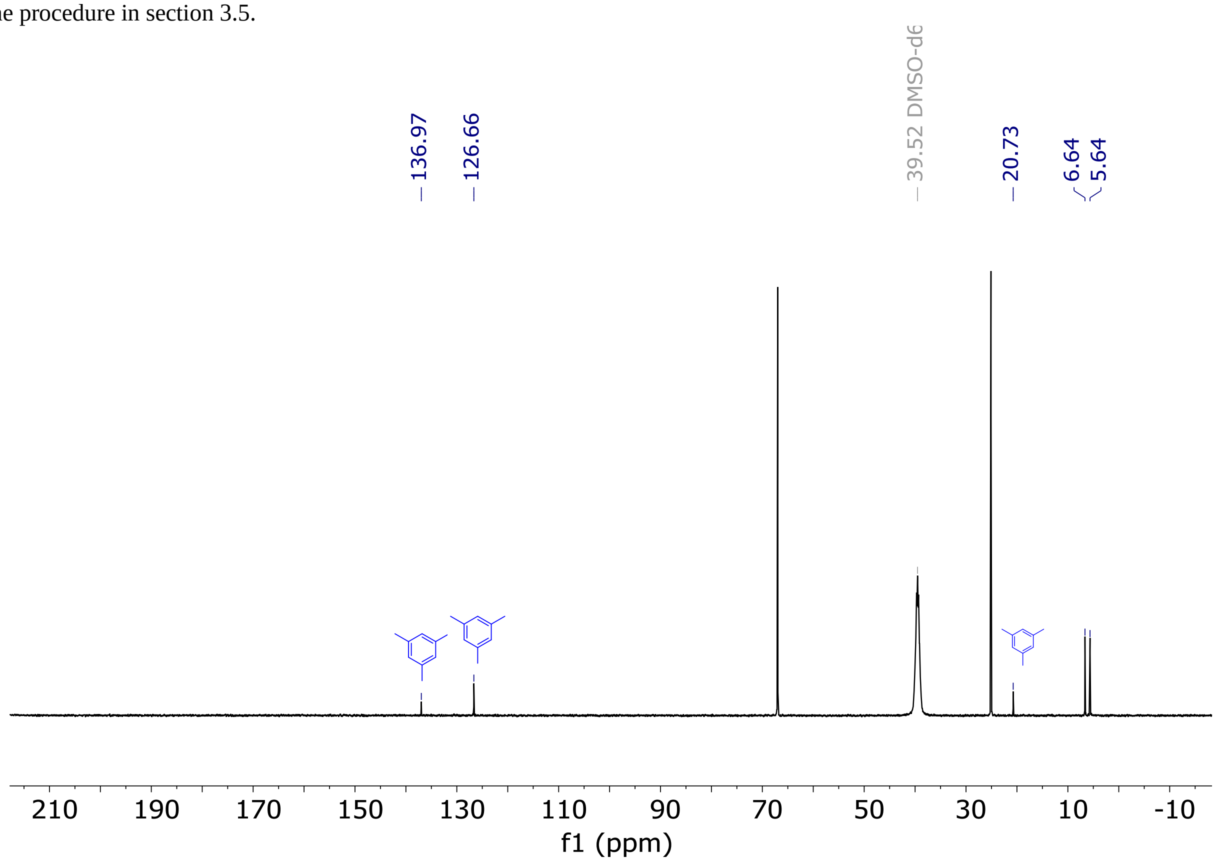

Figure S32: ^{13}C NMR (101 MHz, DMSO-d_6, 298.6 K) spectrum of the crude reaction mixture yielding compound **2i** according to the procedure in section 3.5.

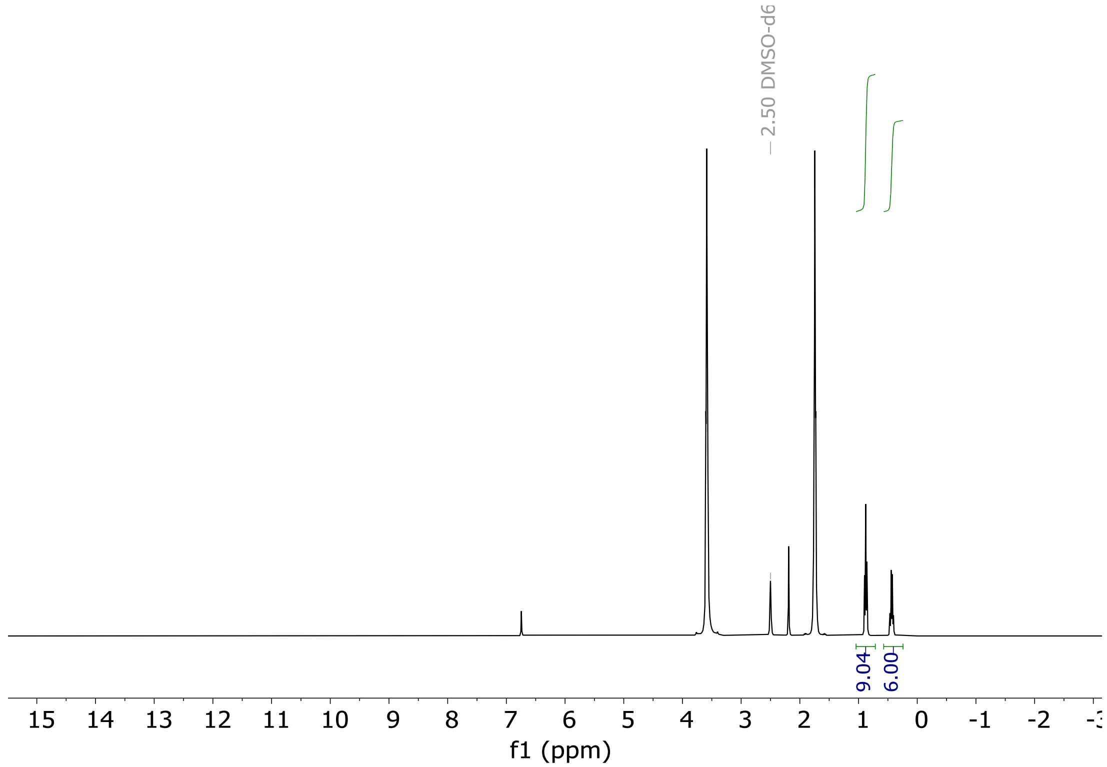

Figure S33: ^{1}H NMR (400 MHz, DMSO-d$_6$ 298.1 K) spectrum of the crude reaction mixture yielding compound **2i** according to the procedure in section 3.5 after adding 0.05 mL of D_2O.

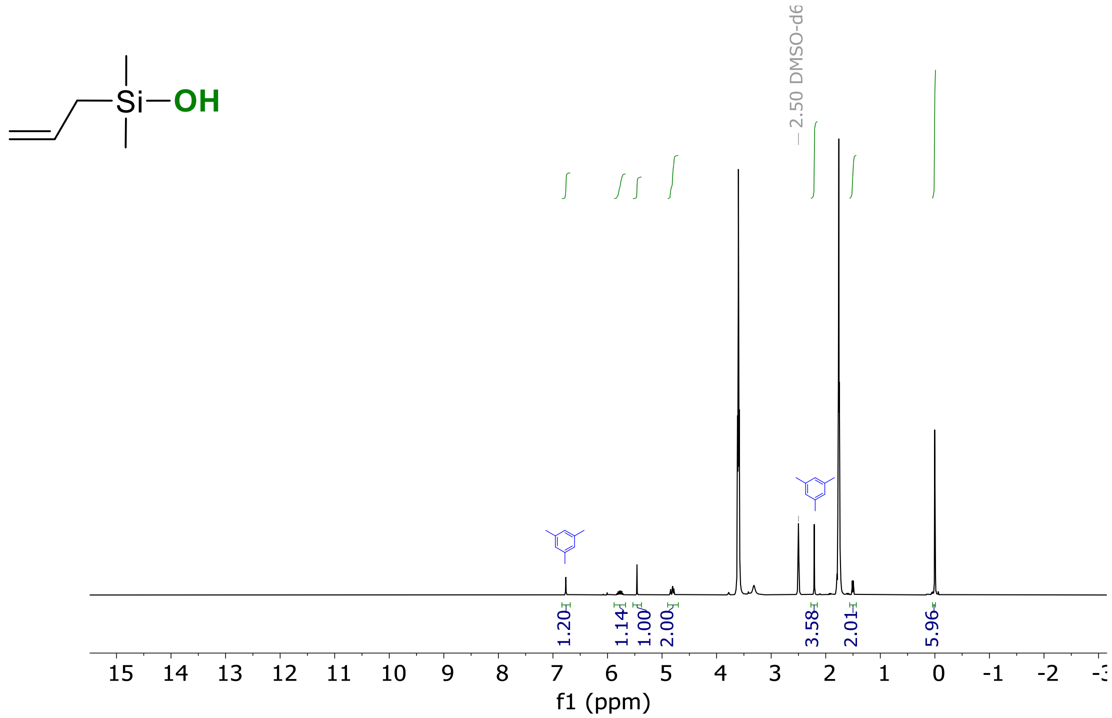

Figure S34: ^{1}H NMR (400 MHz, DMSO-*d$_6$* 298.1 K) spectrum of the crude reaction mixture yielding compound **2k** according to the procedure in section 3.5.

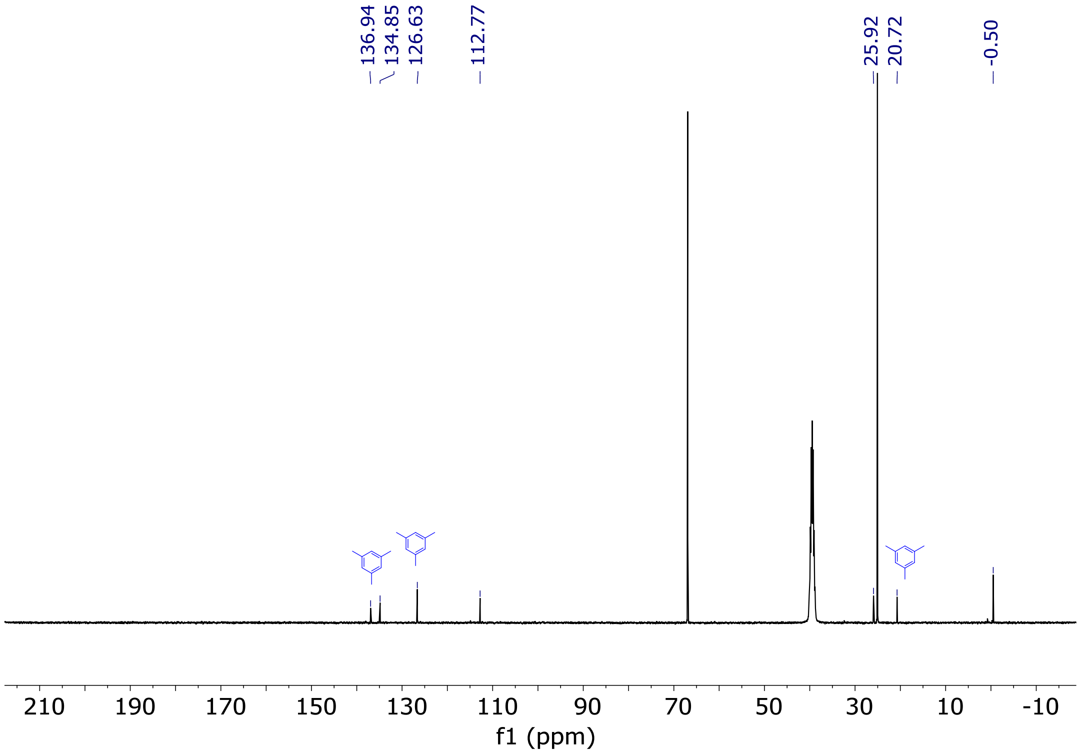

Figure S35: ^{13}C NMR (101 MHz, DMSO-*d*$_6$, 298.5 K) spectrum of the crude reaction mixture yielding compound **2k** according to the procedure in section 3.5.

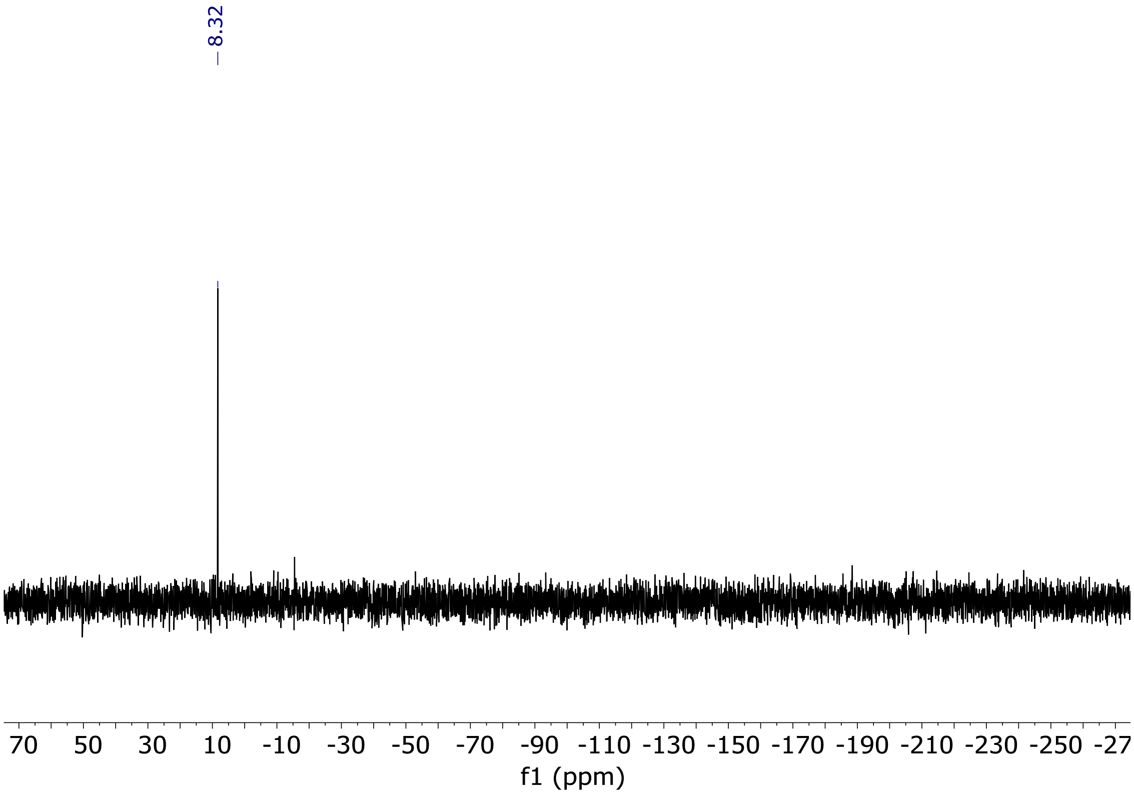

Figure S36: ^{29}Si NMR (79 MHz, DMSO-*d*$_6$, 298.1 K) spectrum of the crude reaction mixture yielding compound **2k** according to the procedure in section 3.5.

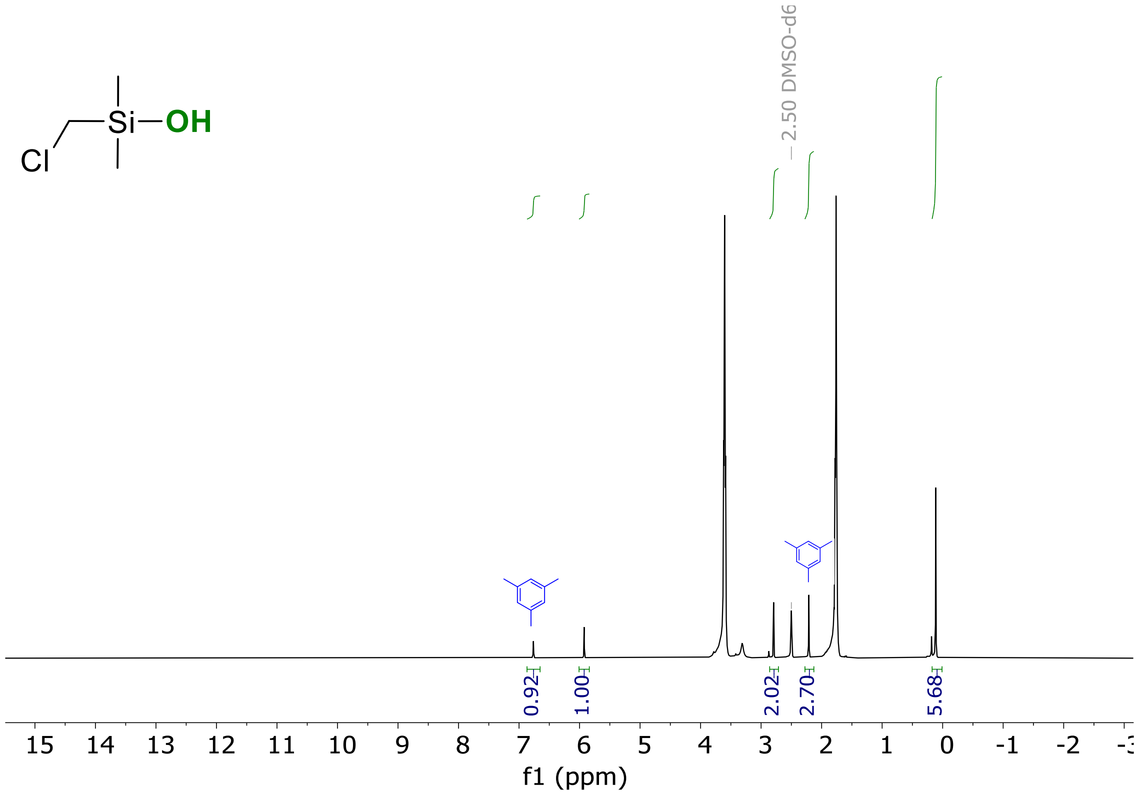

Figure S37: ¹H NMR (400 MHz, DMSO-d_6 298.1 K) spectrum of the crude reaction mixture yielding compound **2m** according to the procedure in section 3.5.

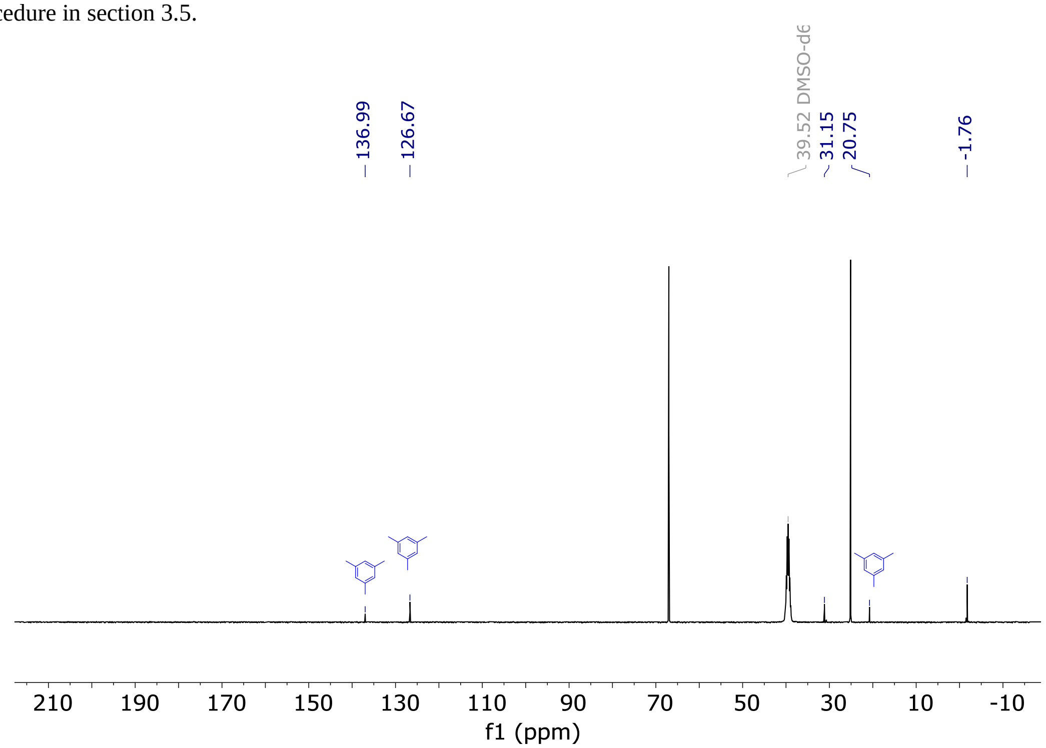

Figure S38: ¹³C NMR (101 MHz, DMSO-d_6, 298.5 K) spectrum of the crude reaction mixture yielding compound **2m** according to the procedure in section 3.5.

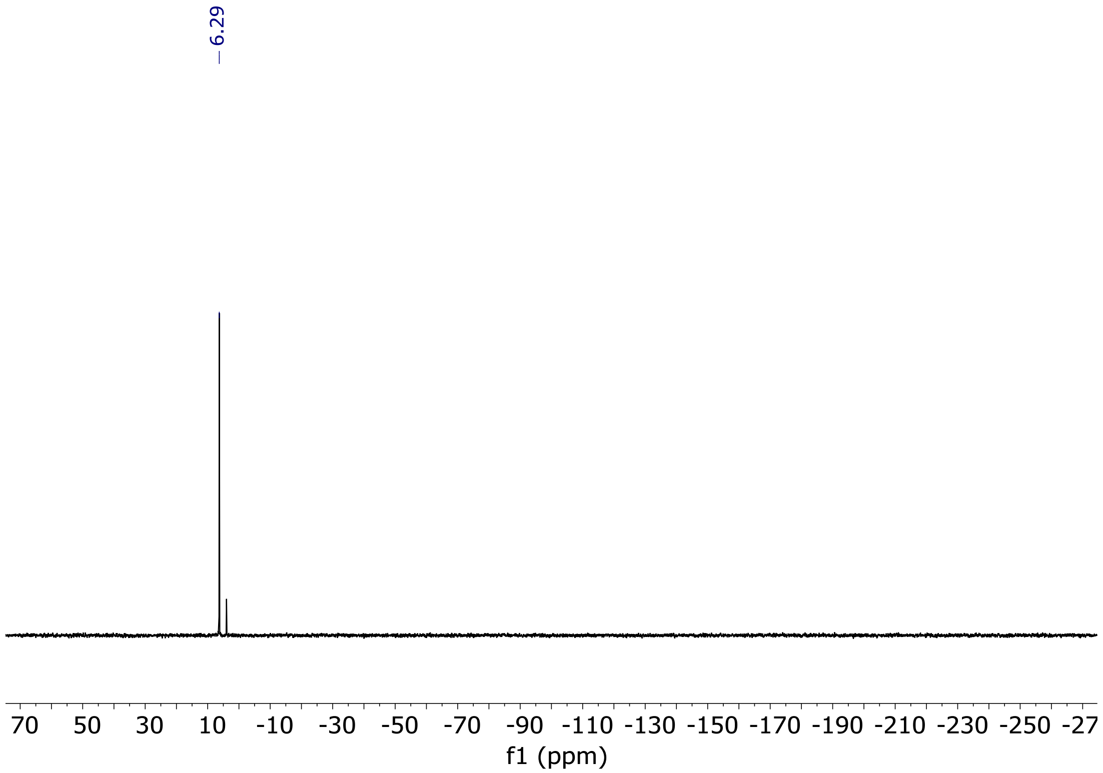

Figure S39: ^{29}Si NMR (79 MHz, DMSO-*d*$_6$, 298.1 K) spectrum of the crude reaction mixture yielding compound **2m** according to the procedure in section 3.5.

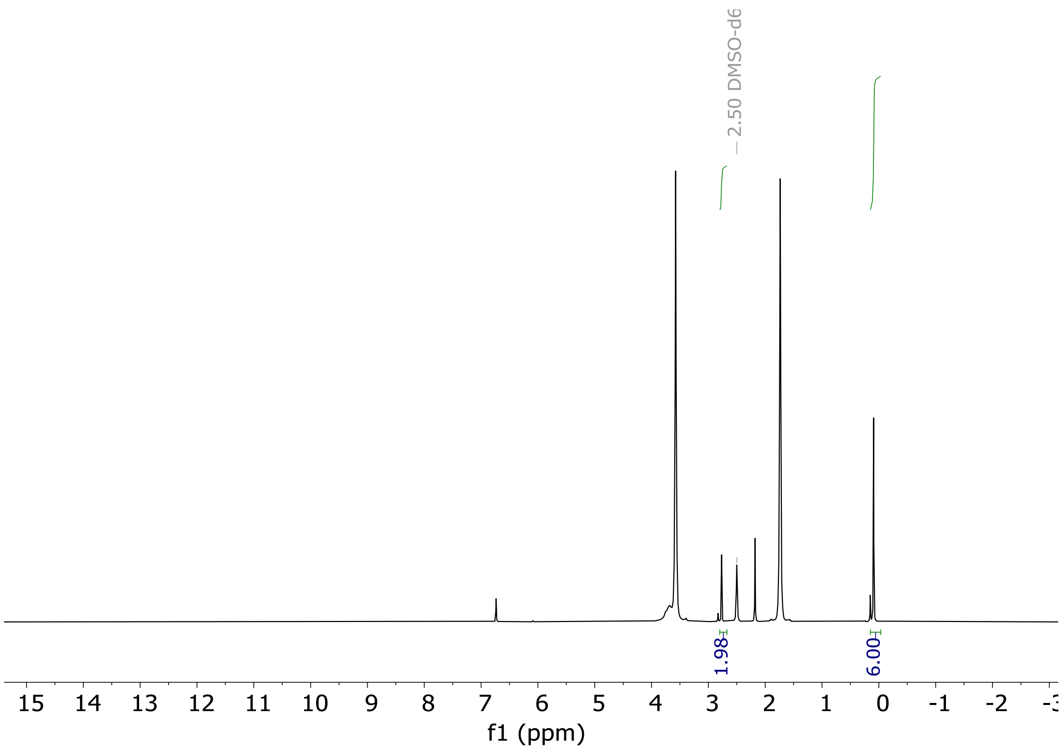

Figure S40: ^{1}H NMR (400 MHz, DMSO-*d*$_6$ 298.1 K) spectrum of the crude reaction mixture yielding compound **2m** according to the procedure in section 3.5 after adding 0.05 mL of D_2O.

12.2. NMR Spectra of Isolated Silanols

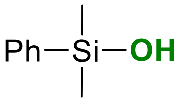

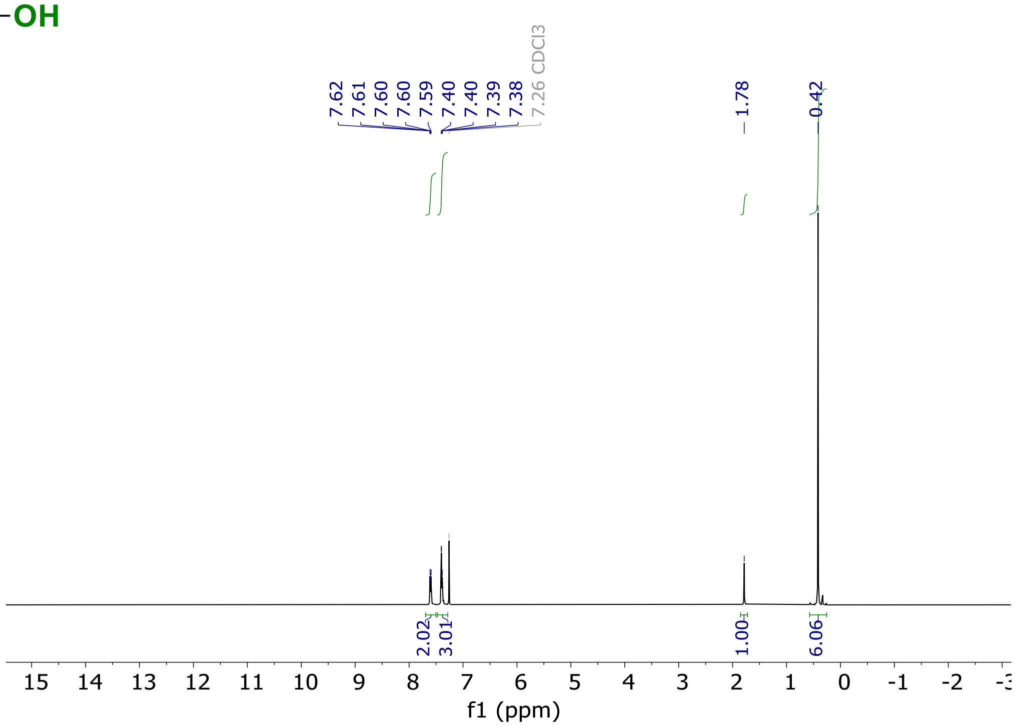

Figure S41: ^{1}H NMR (400 MHz, Chloroform-*d*, 296 K) spectrum of compound **2a**.

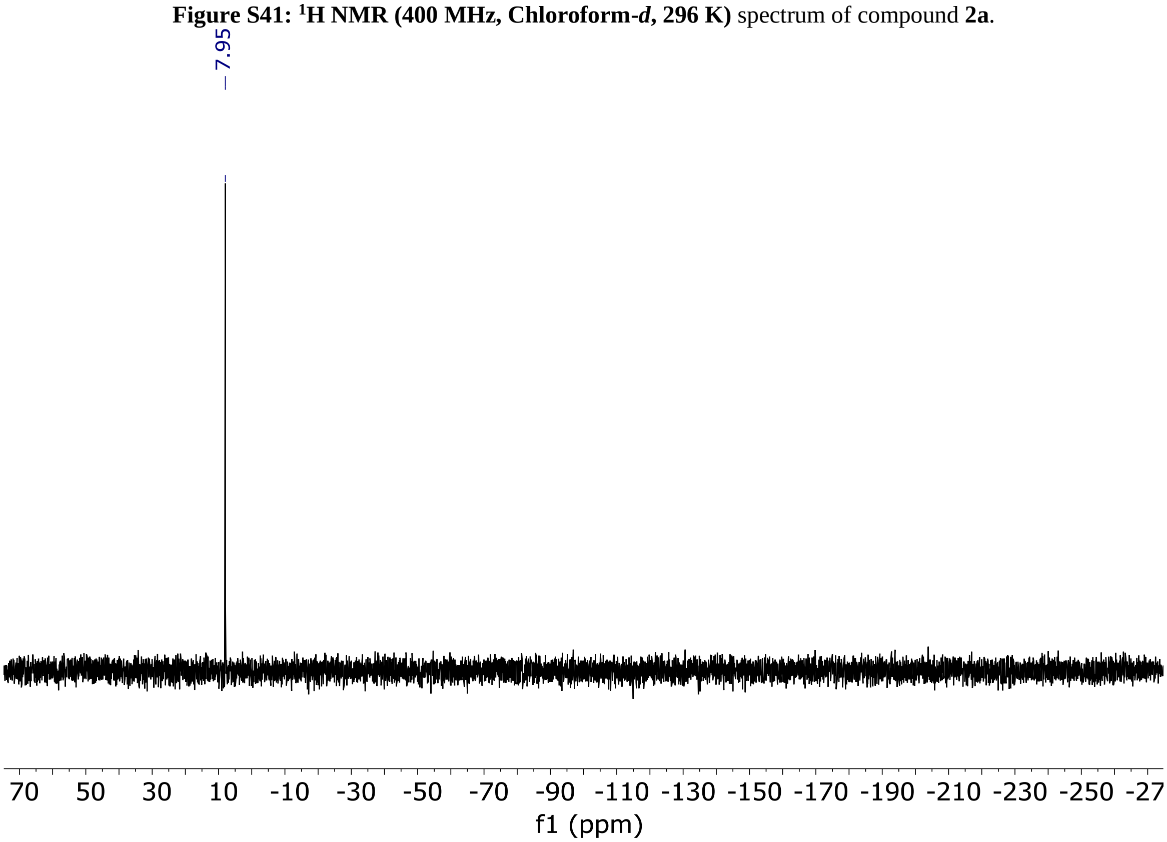

Figure S42: ^{29}Si NMR (79 MHz, Chloroform-*d*, 296 K) spectrum of compound **2a**.

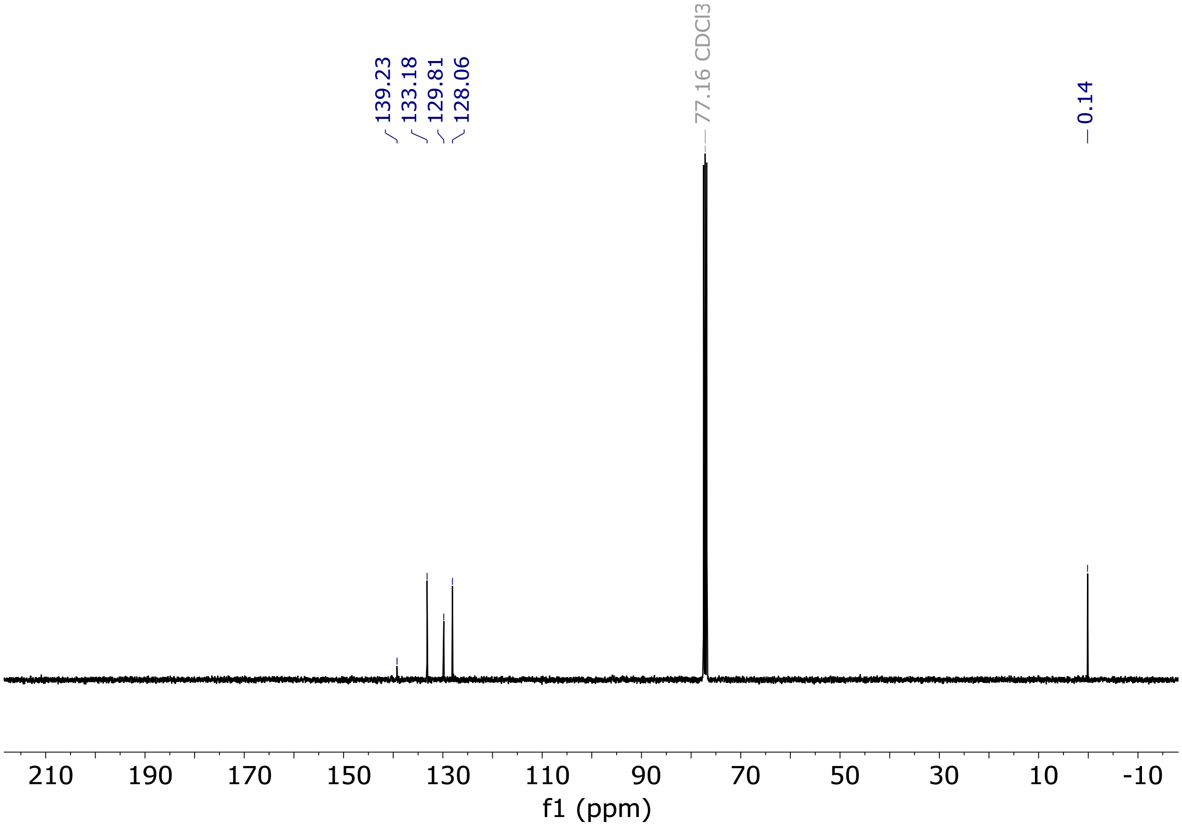

Figure S43: $^{13}C\{^{1}H\}$ NMR (101 MHz, Chloroform-*d*, 296 K) spectrum of compound **2a**.

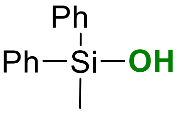

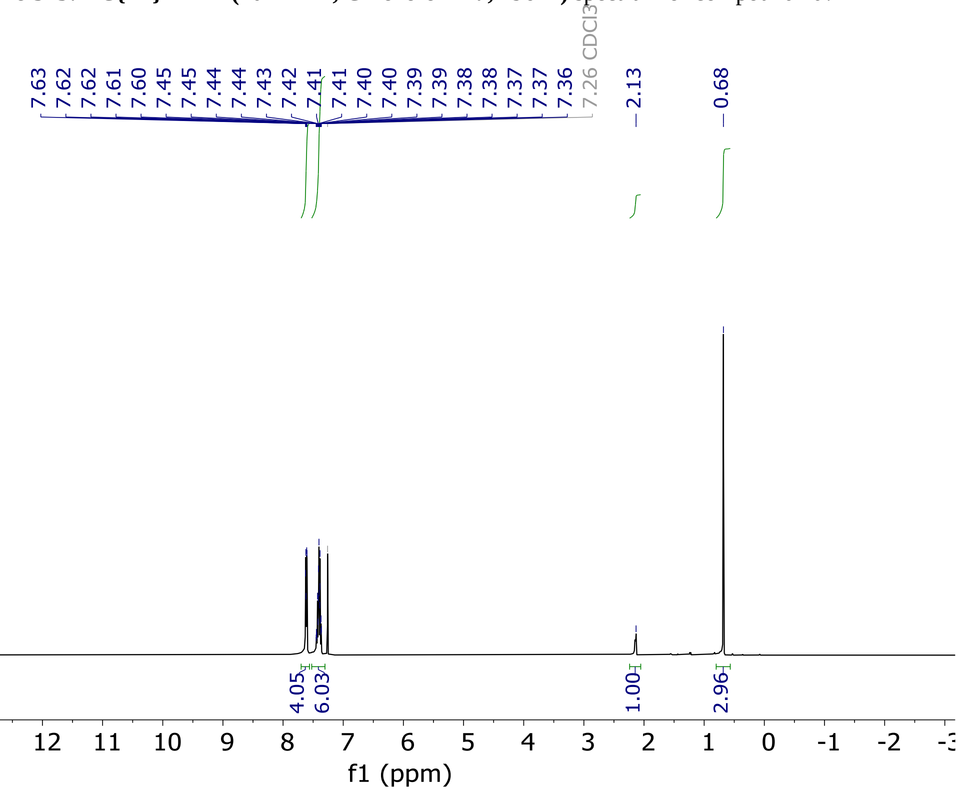

Figure S44: ^{1}H NMR (400 MHz, Chloroform-*d*, 296 K) spectrum of compound **2b**.

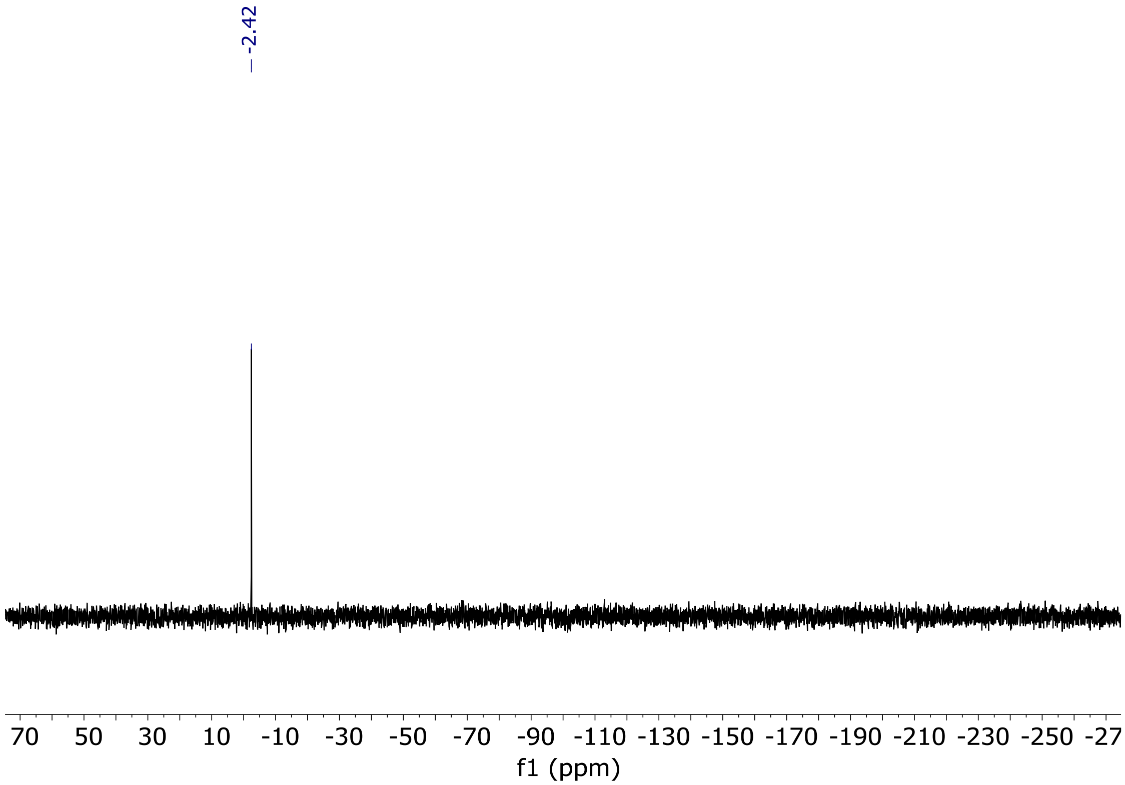

Figure S45: ^{29}Si NMR (79 MHz, Chloroform-*d*, 296K) spectrum of compound **2b**.

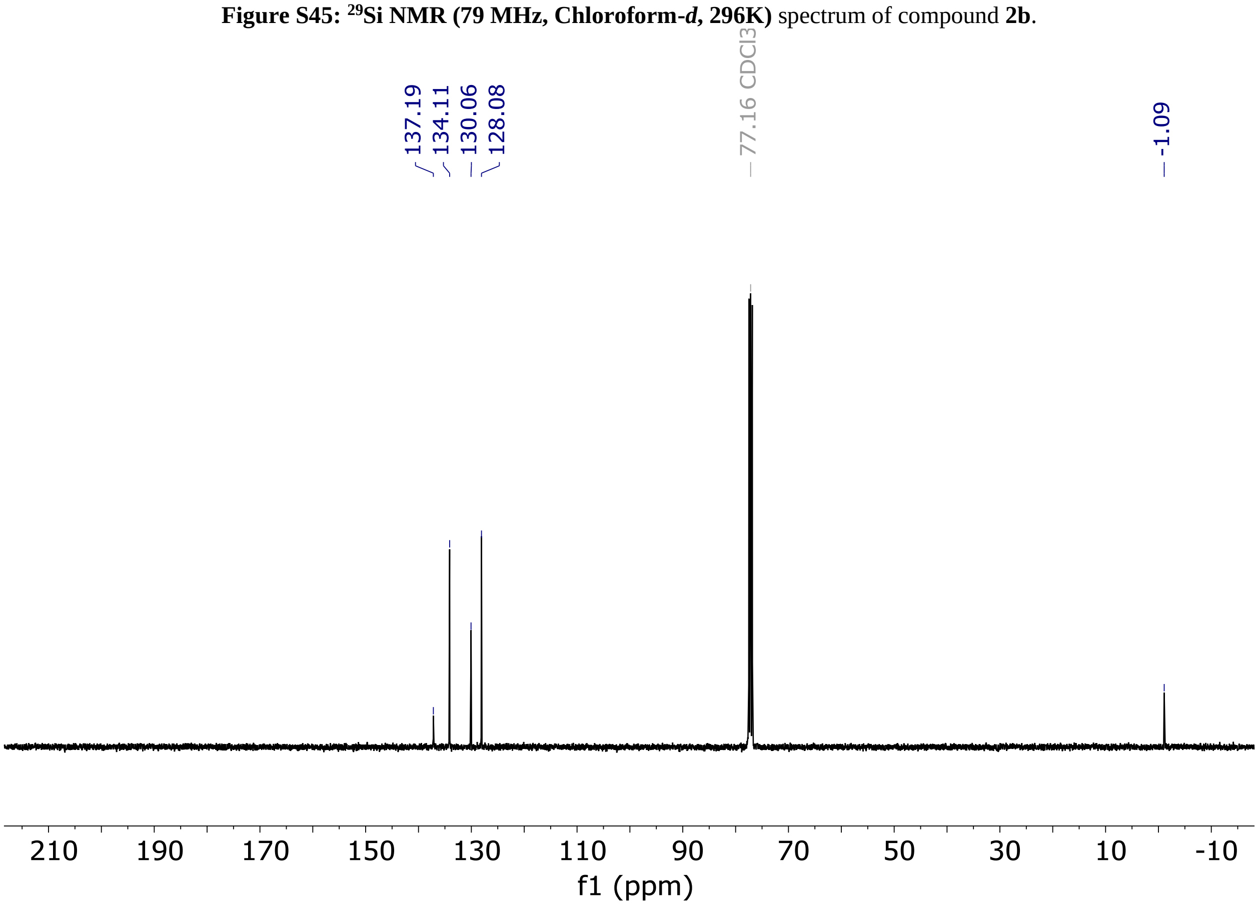

Figure S46: ^{13}C{^{1}H} NMR (101 MHz, Chloroform-*d*, 296 K) spectrum of compound **2b**.

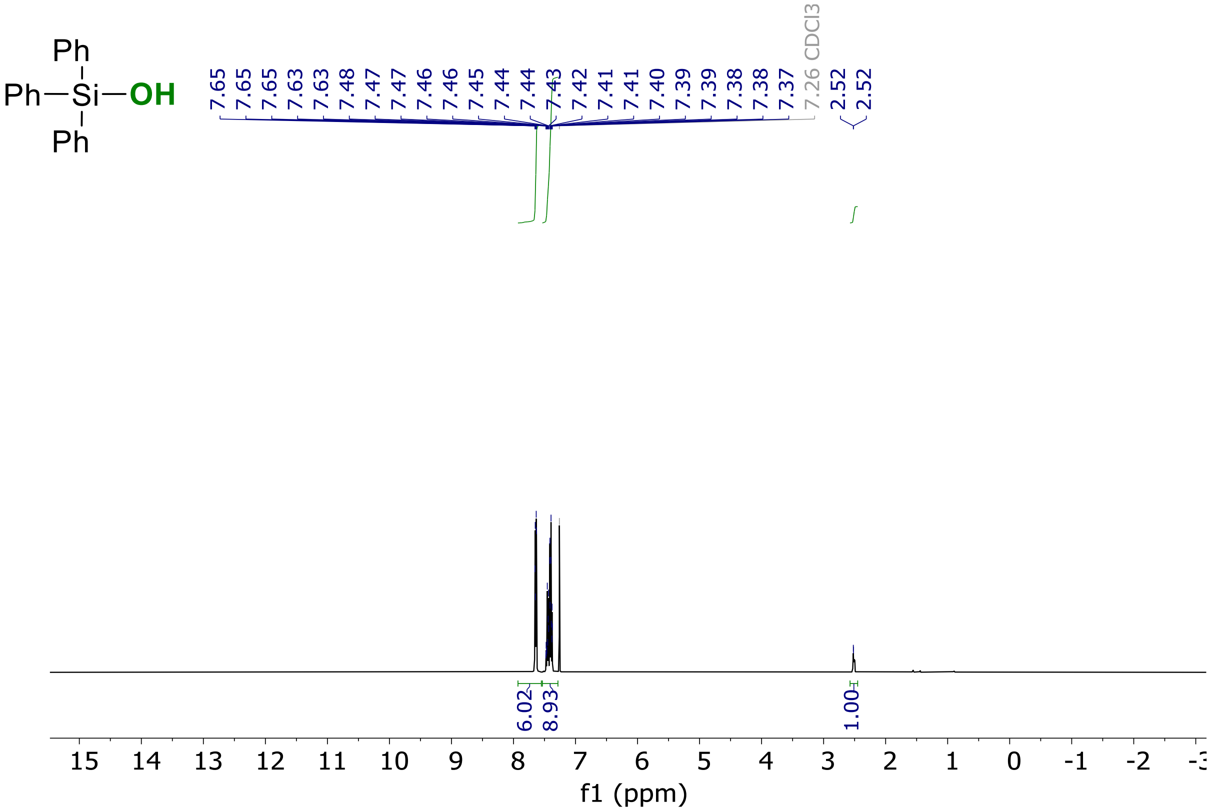

Figure S47: ^{1}H NMR (400 MHz, Chloroform-*d*, 296 K) spectrum of compound **2c**.

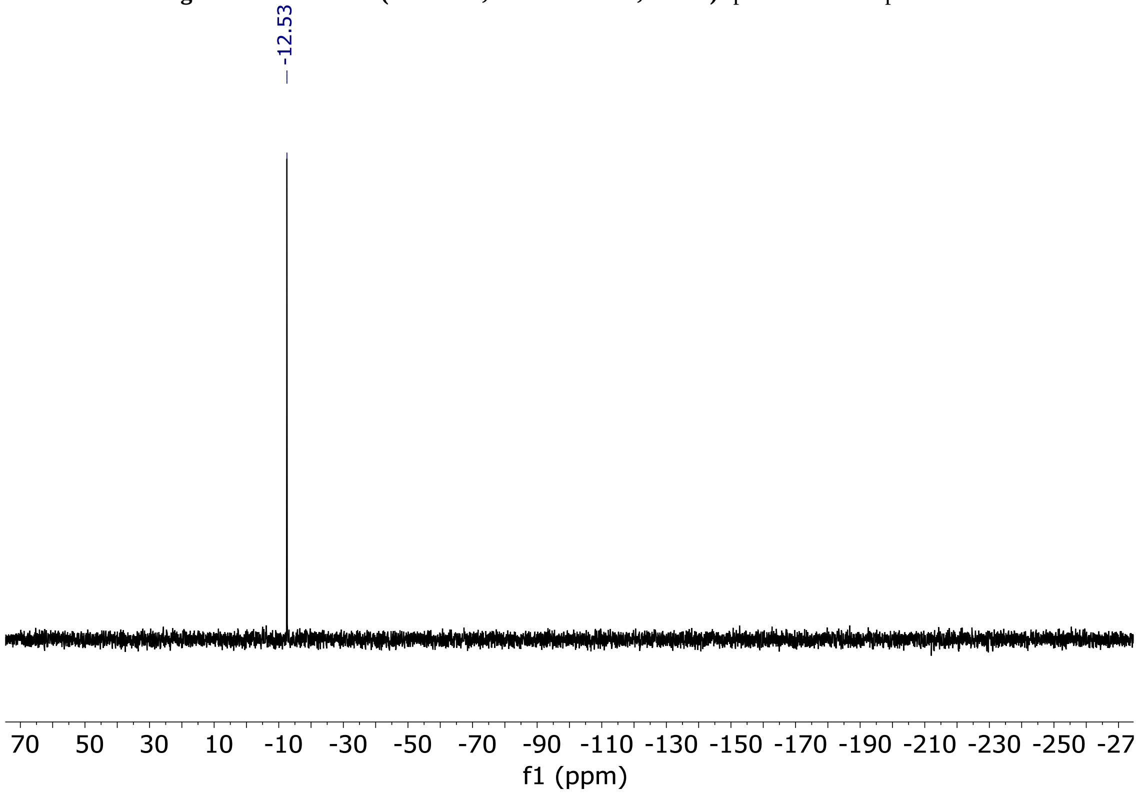

Figure S48: ^{29}Si NMR (79 MHz, Chloroform-*d*, 296K) spectrum of compound **2c**.

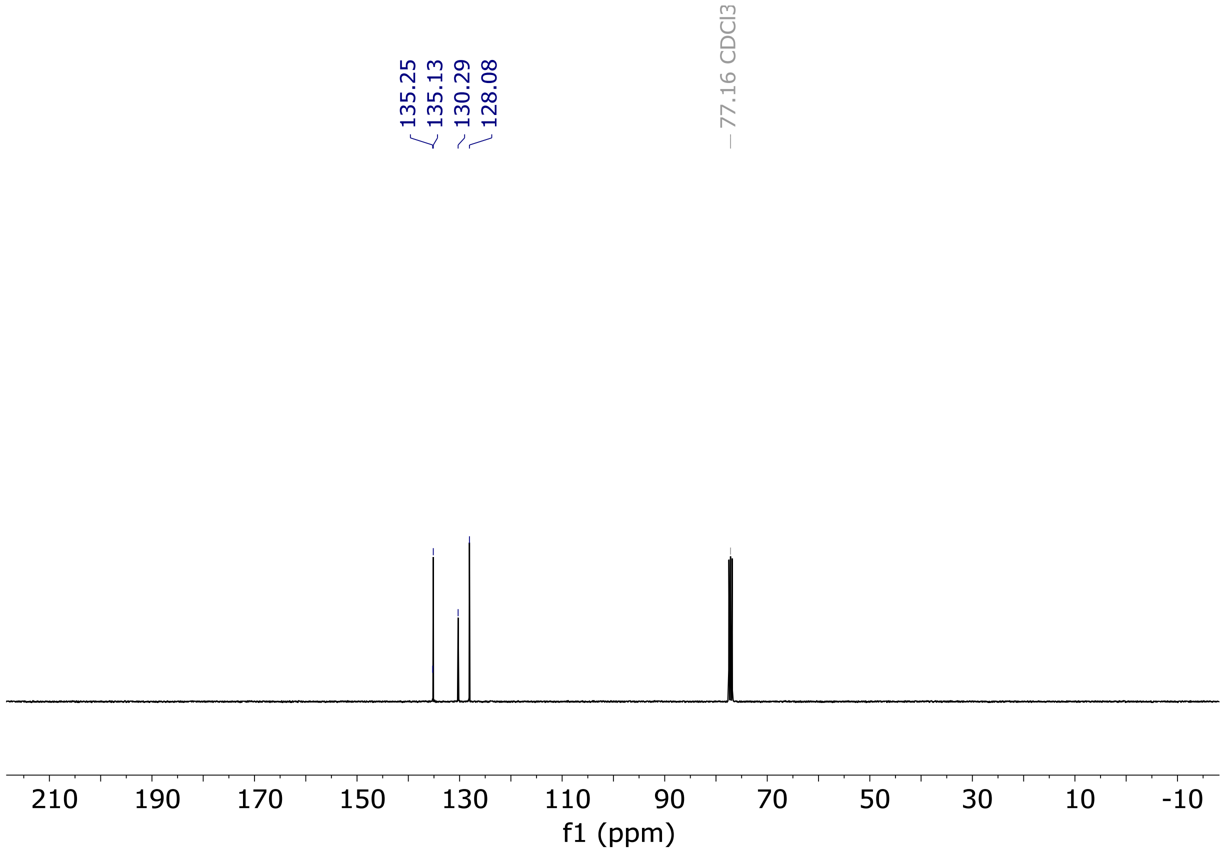

Figure S49: $^{13}C\{^{1}H\}$ NMR (101 MHz, Chloroform-*d*, 296 K) spectrum of compound **2c**.

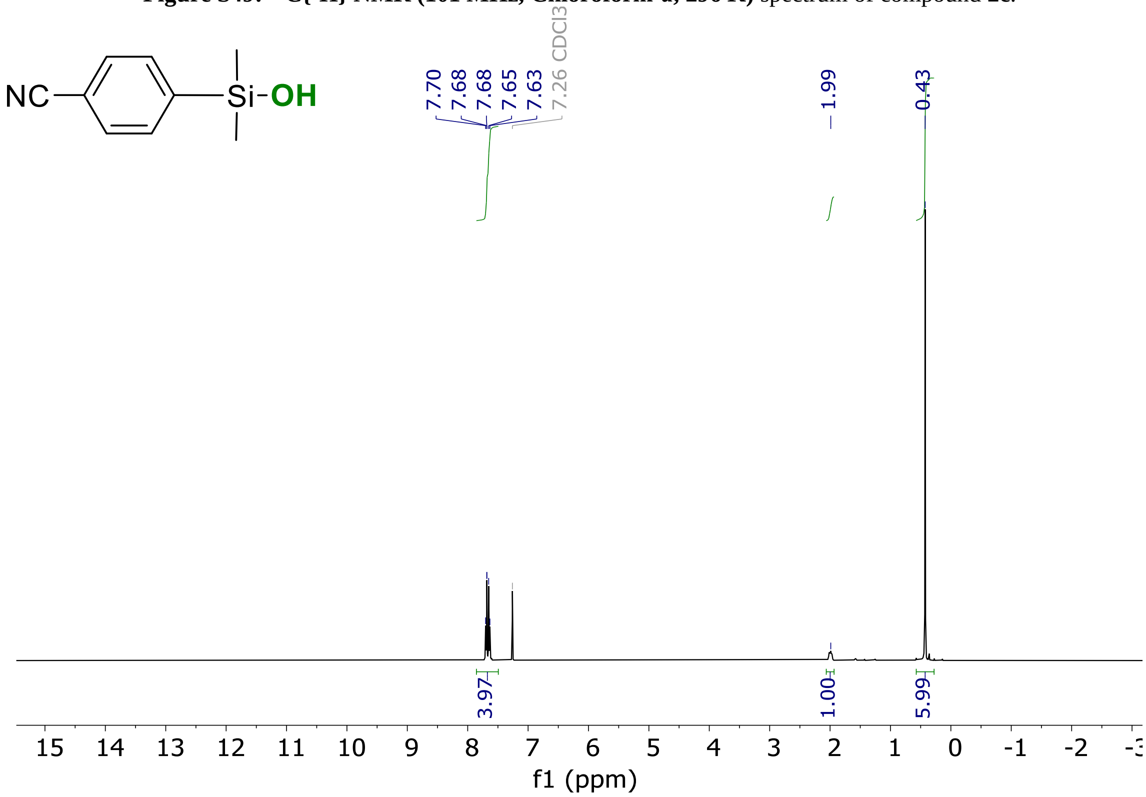

Figure S50: ^{1}H NMR (400 MHz, Chloroform-*d*, 296 K) spectrum of compound **2d**.

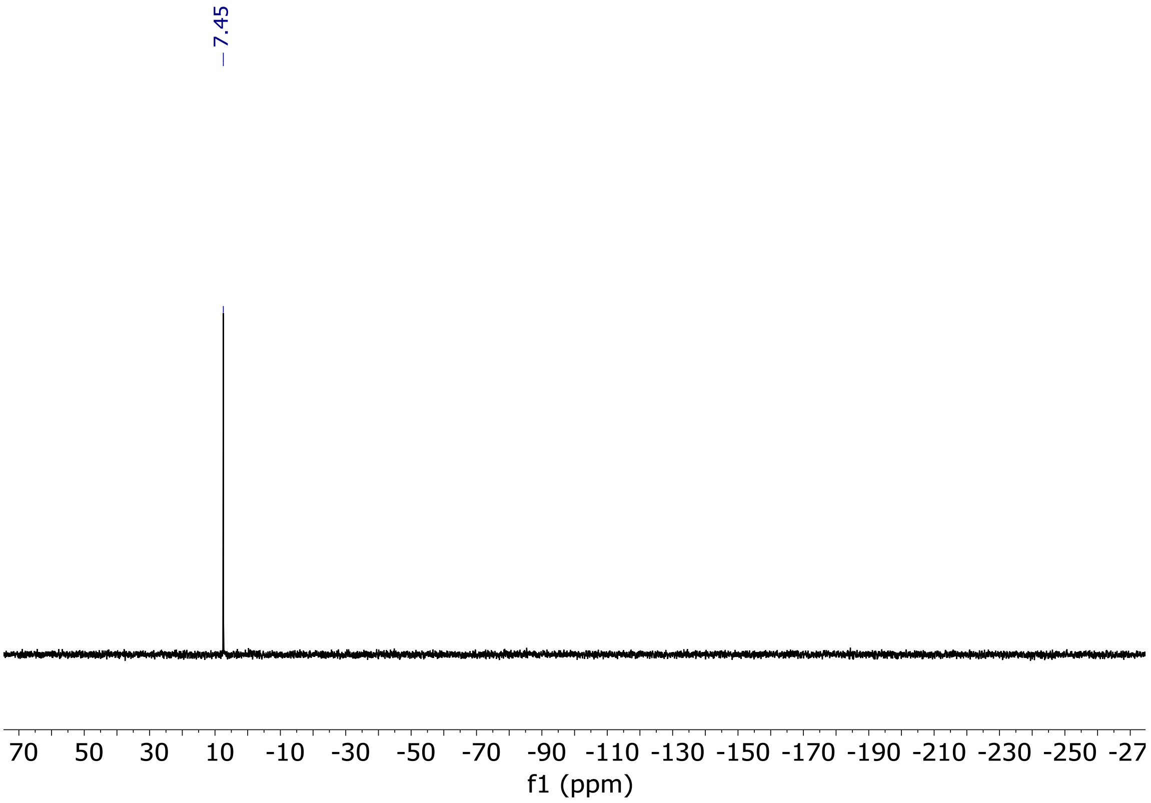

Figure S51: ^{29}Si NMR (79 MHz, Chloroform-*d*, 296K) spectrum of compound **2d**.

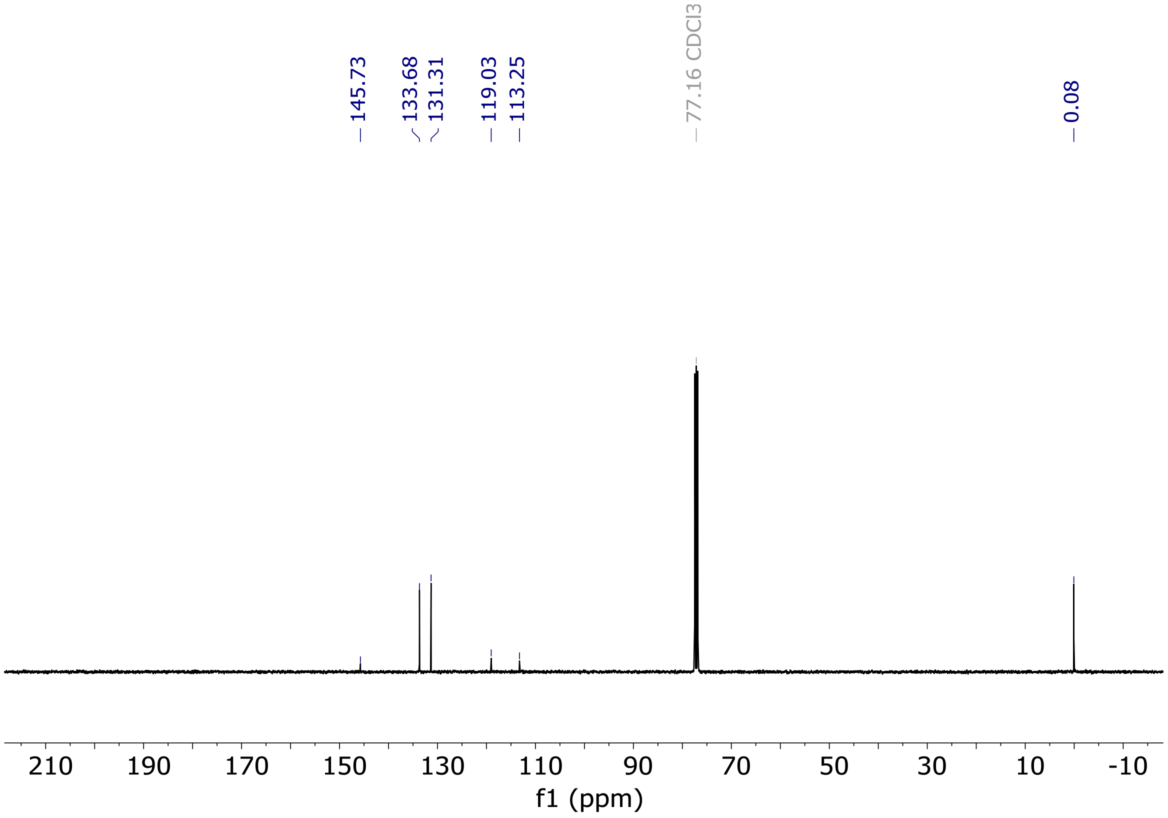

Figure S52: ^{13}C{^{1}H} NMR (101 MHz, Chloroform-*d*, 296 K) spectrum of compound **2d**.

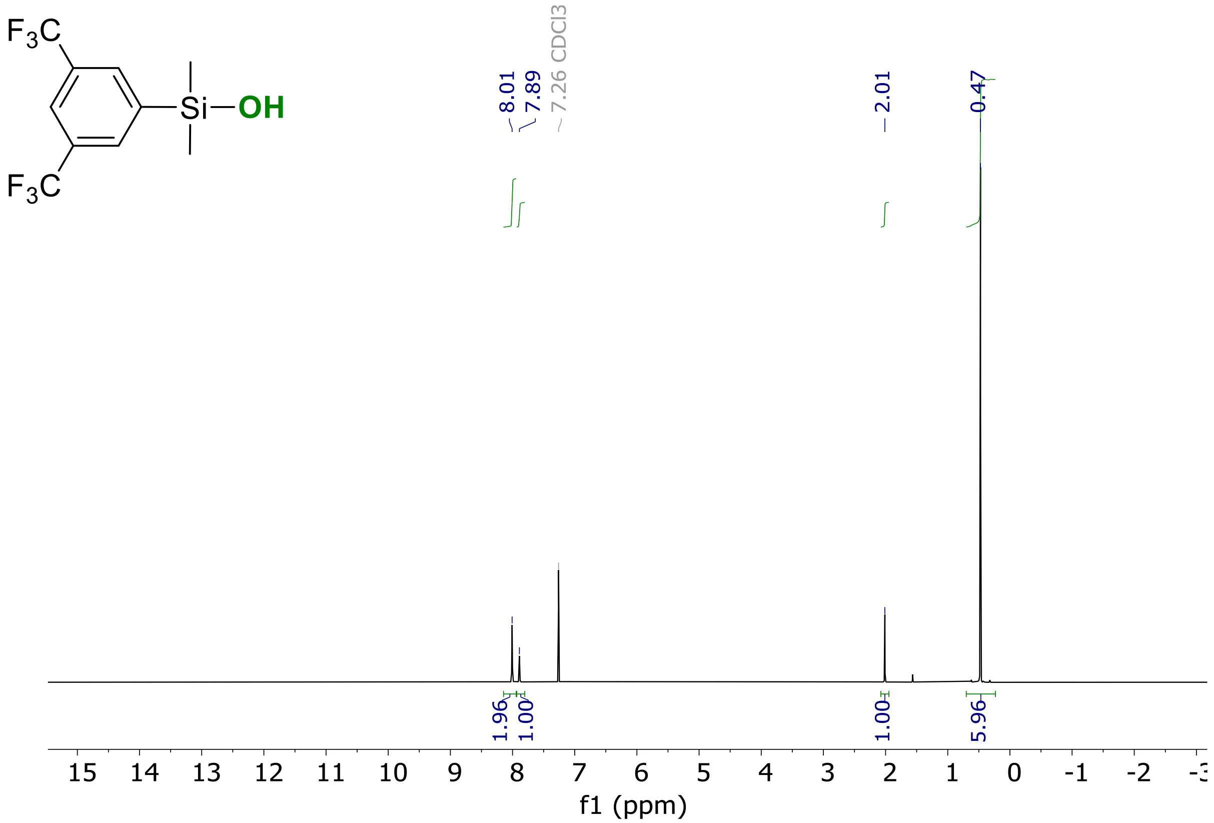

Figure S53: ^{1}H NMR (400 MHz, Chloroform-*d*, 296 K) spectrum of compound **2e**.

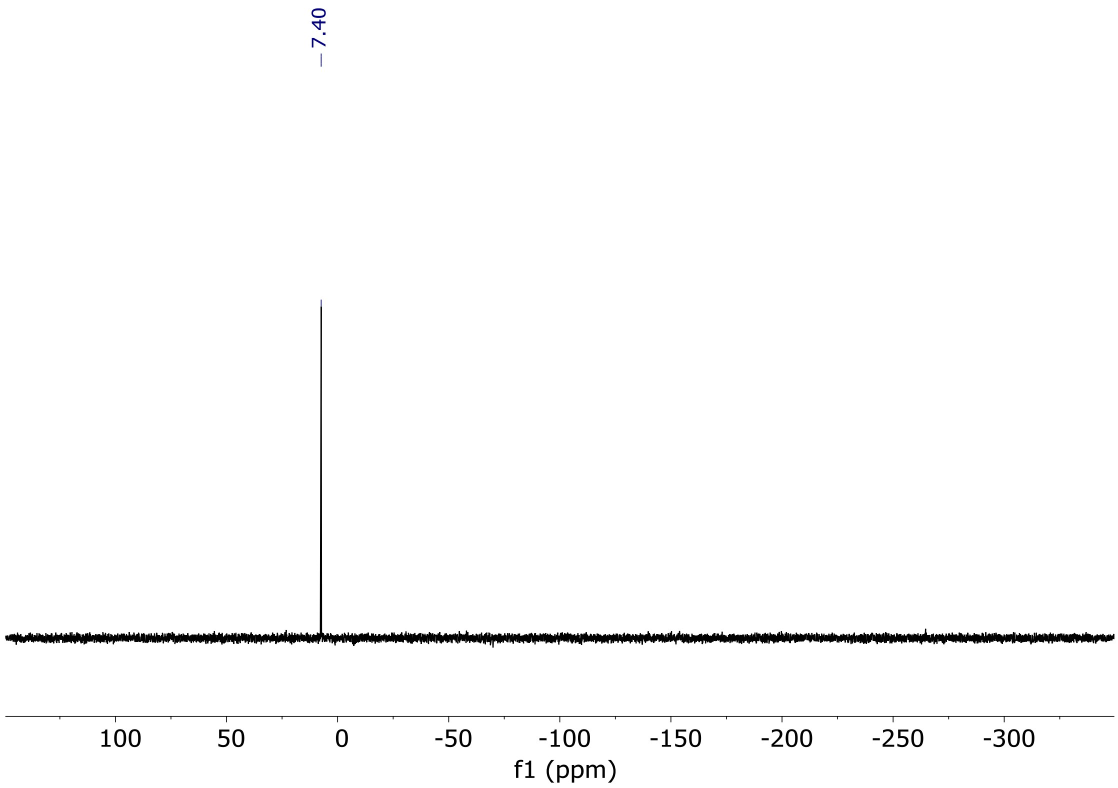

Figure S54: ^{29}Si NMR (79 MHz, Chloroform-*d*, 296K) spectrum of compound **2e**.

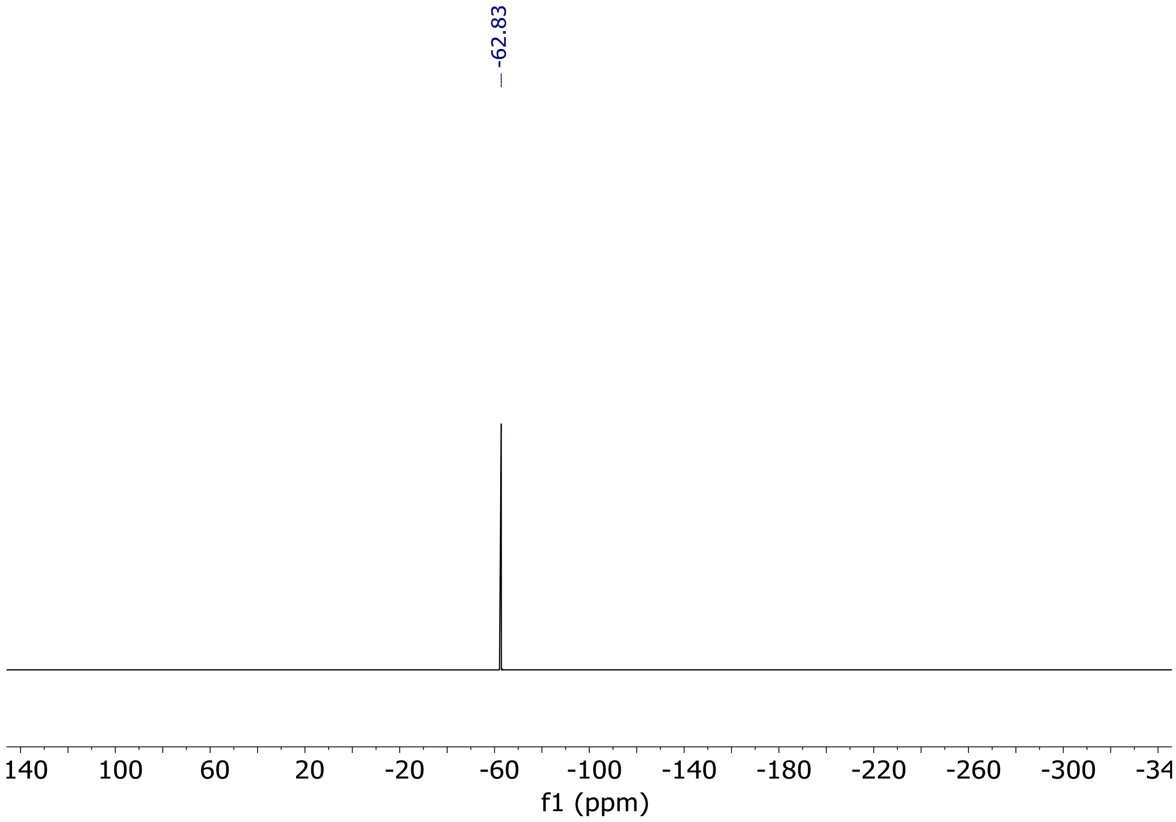

Figure S55: ^{19}F NMR (376 MHz, Chloroform-*d*, 296K): spectrum of compound **2e**.

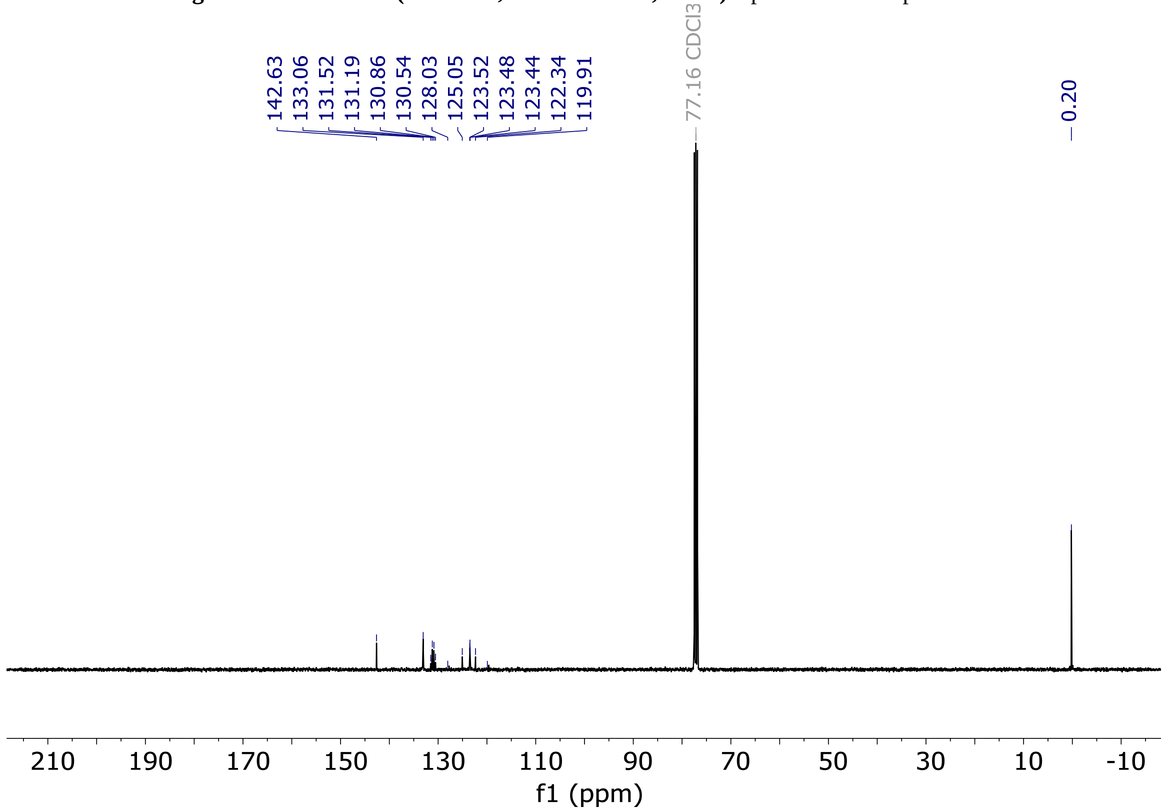

Figure S56: ^{13}C{^{1}H} NMR (101 MHz, Chloroform-*d*, 296 K) spectrum of compound **2e**.

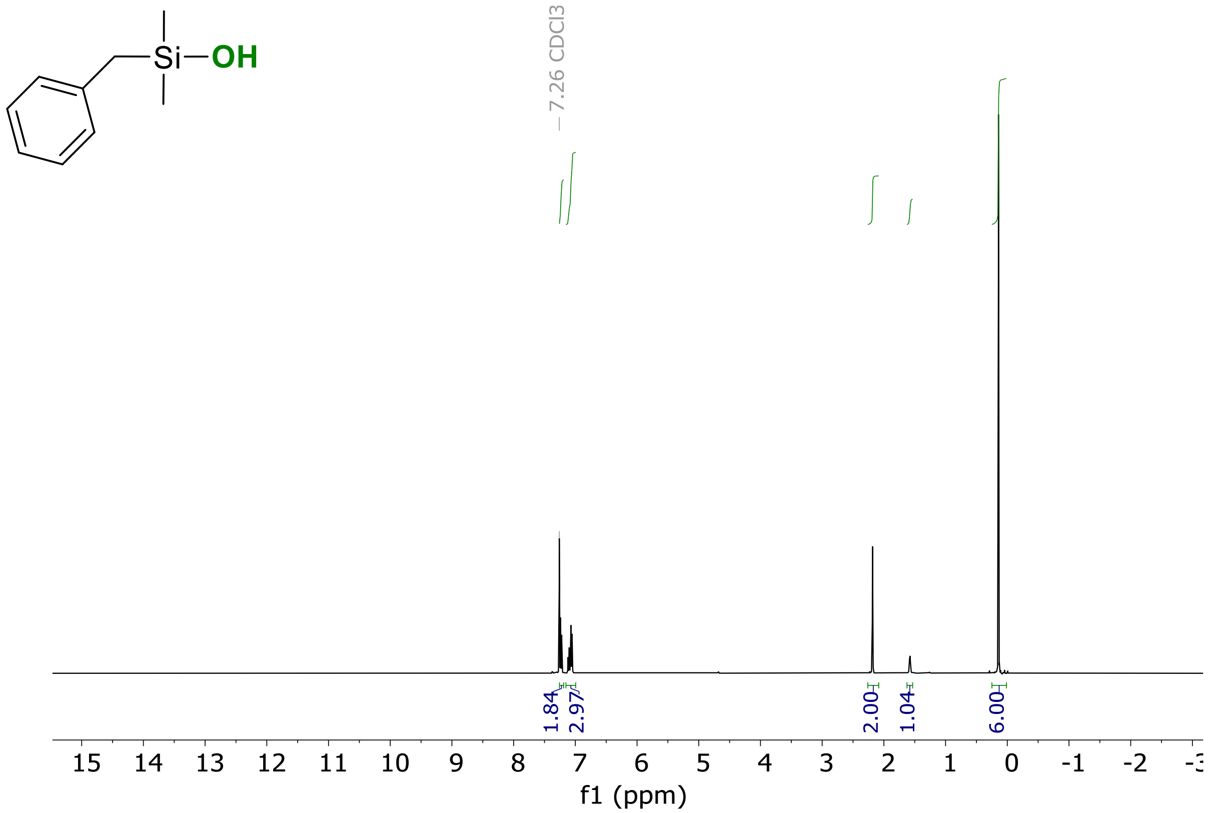

Figure S57: ^{1}H NMR (400 MHz, Chloroform-*d*, 296 K) spectrum of compound **2f**.

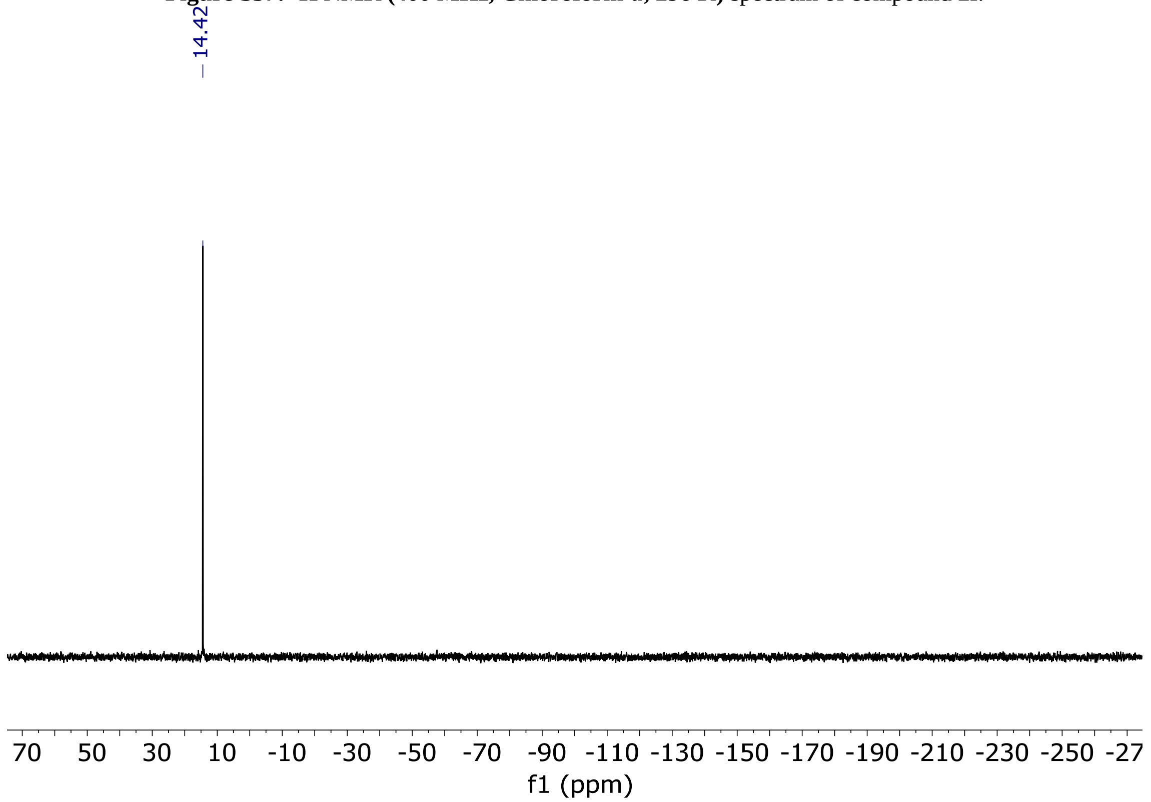

Figure S58: ^{29}Si NMR (79 MHz, Chloroform-*d*, 296K) Spectrum of compound **2f**.

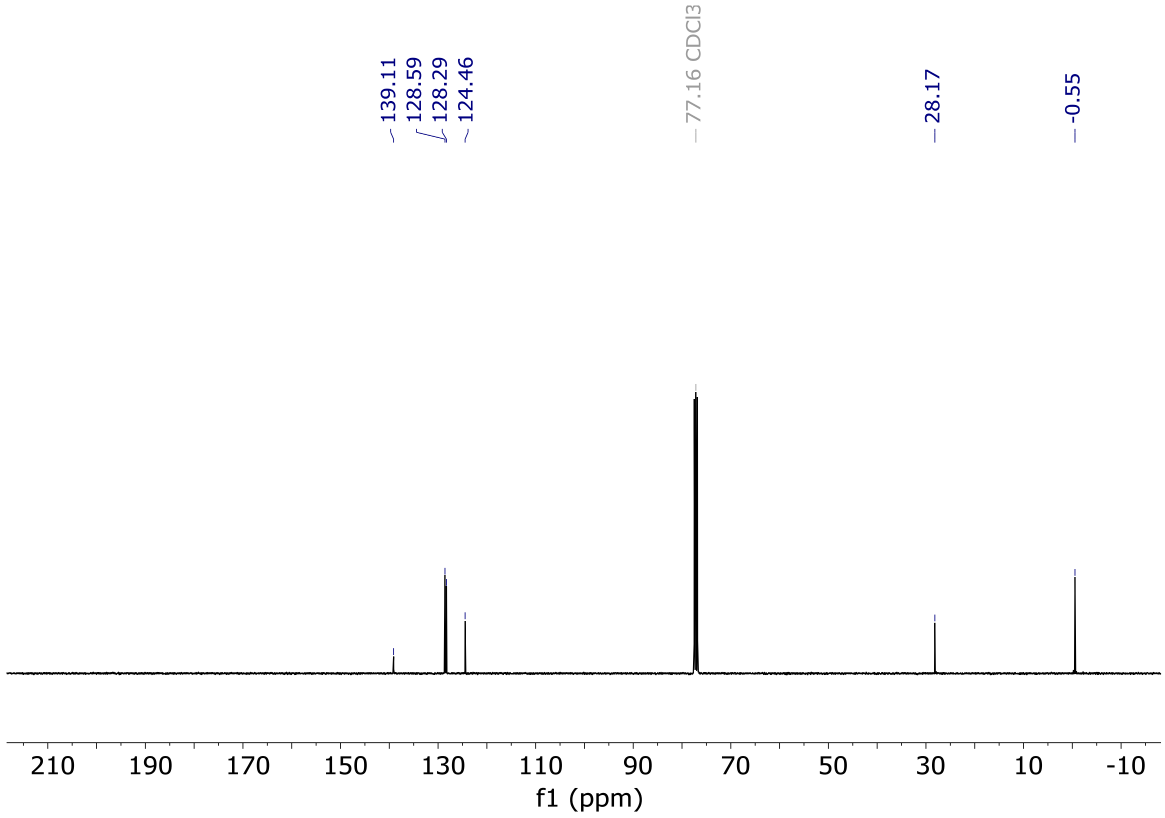

Figure S59: $^{13}C\{^{1}H\}$ **NMR (101 MHz, Chloroform-*d*, 296 K)** spectrum of compound **2f**.

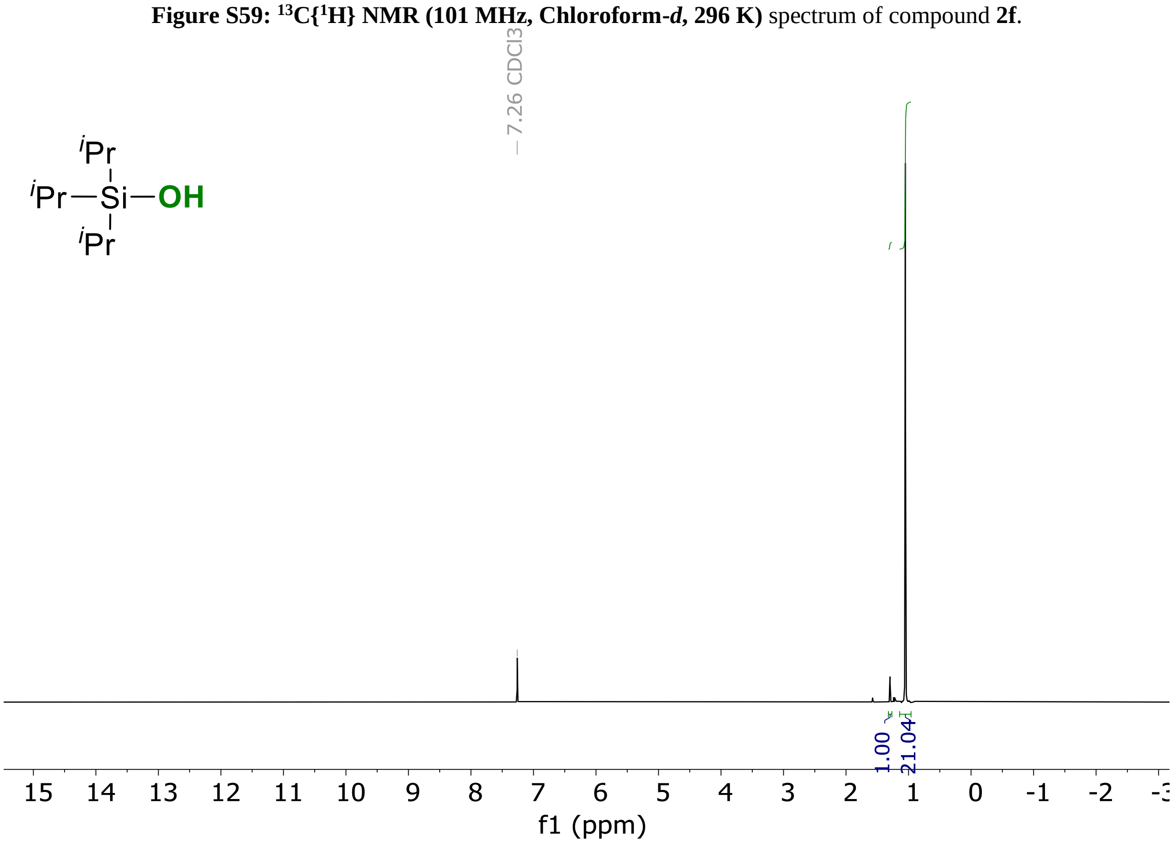

Figure S60: ^{1}H **NMR (400 MHz, Chloroform-*d*, 296 K)** spectrum of compound **2j**.

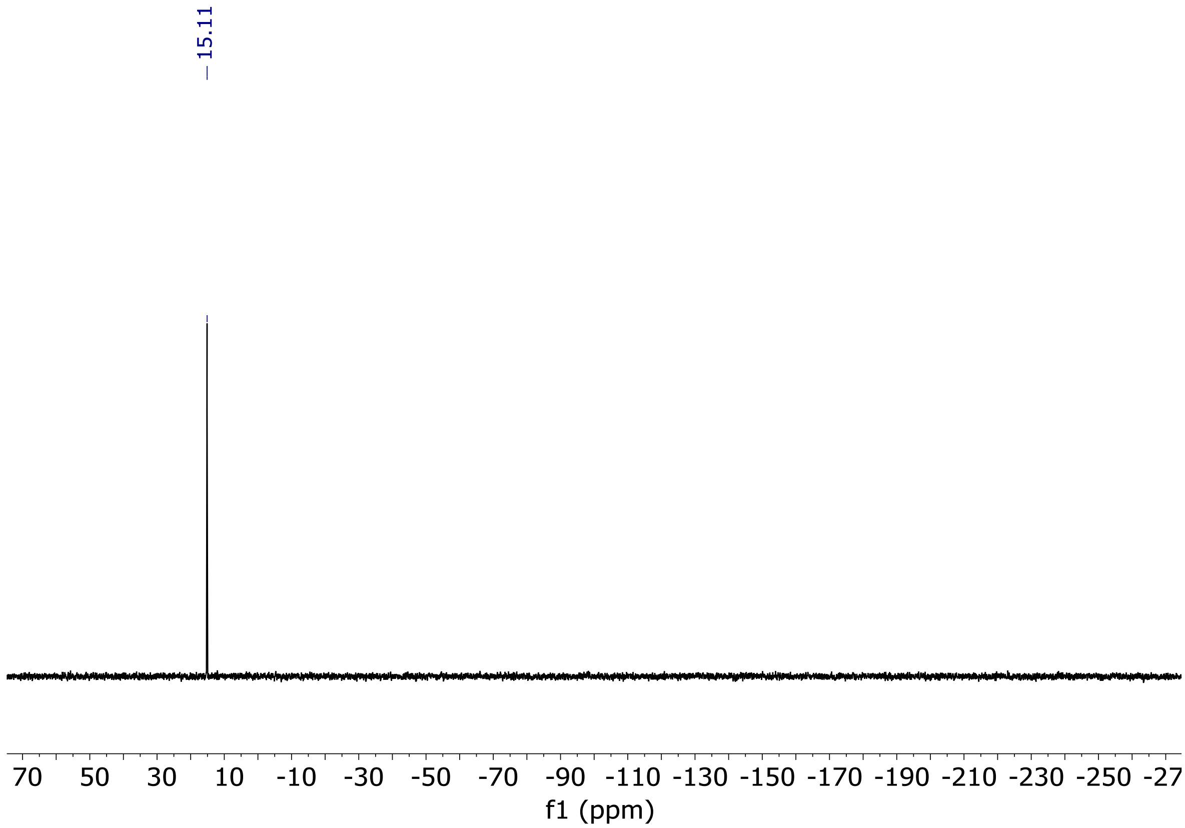

Figure S61: ^{29}Si NMR (79 MHz, Chloroform-*d*, 296K) spectrum of compound **2j**.

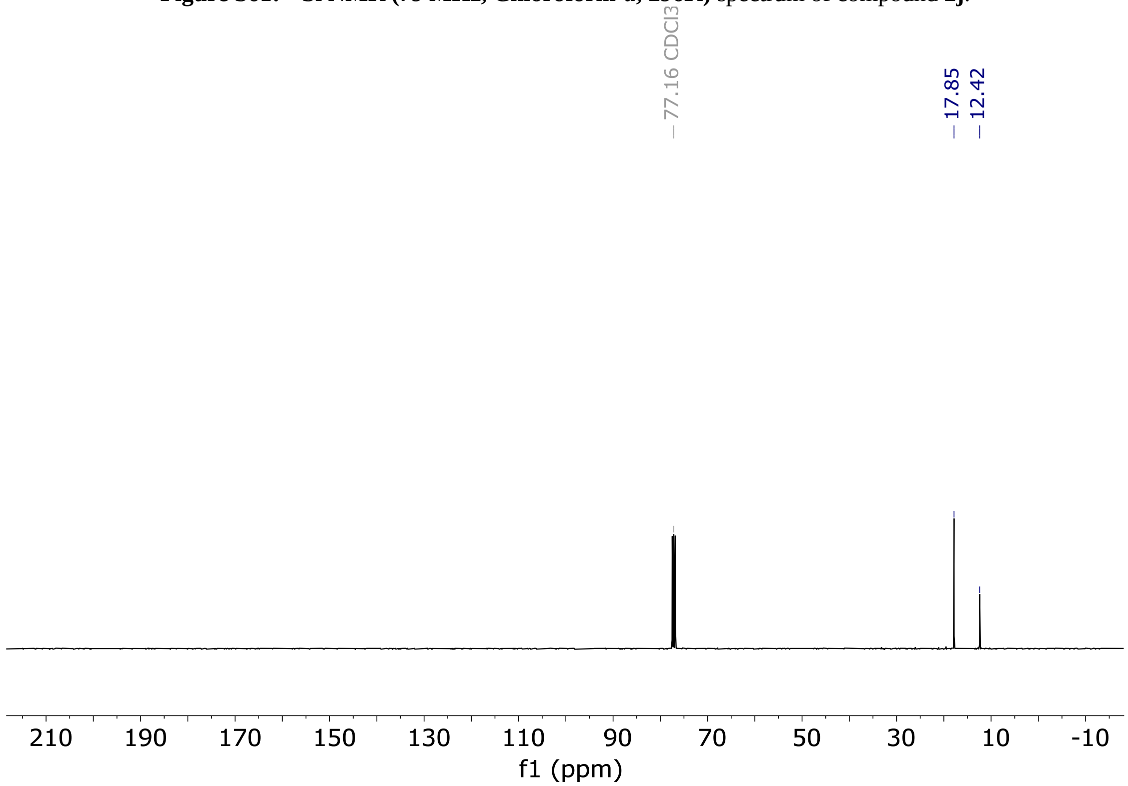

Figure S62: ^{13}C{^{1}H} NMR (101 MHz, Chloroform-*d*, 296 K) spectrum of compound **2j**.

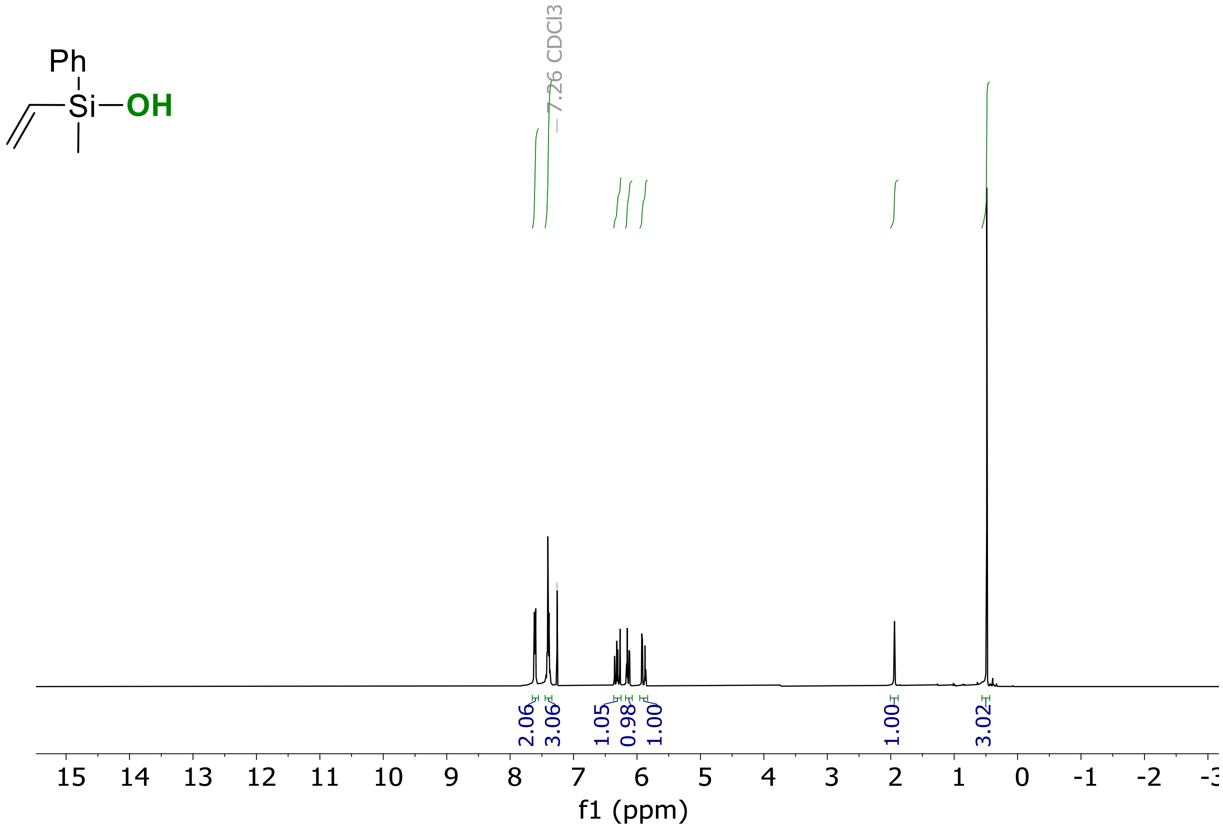

Figure S63: ^{1}H NMR (400 MHz, Chloroform-*d*, 296 K) spectrum of compound **2l**.

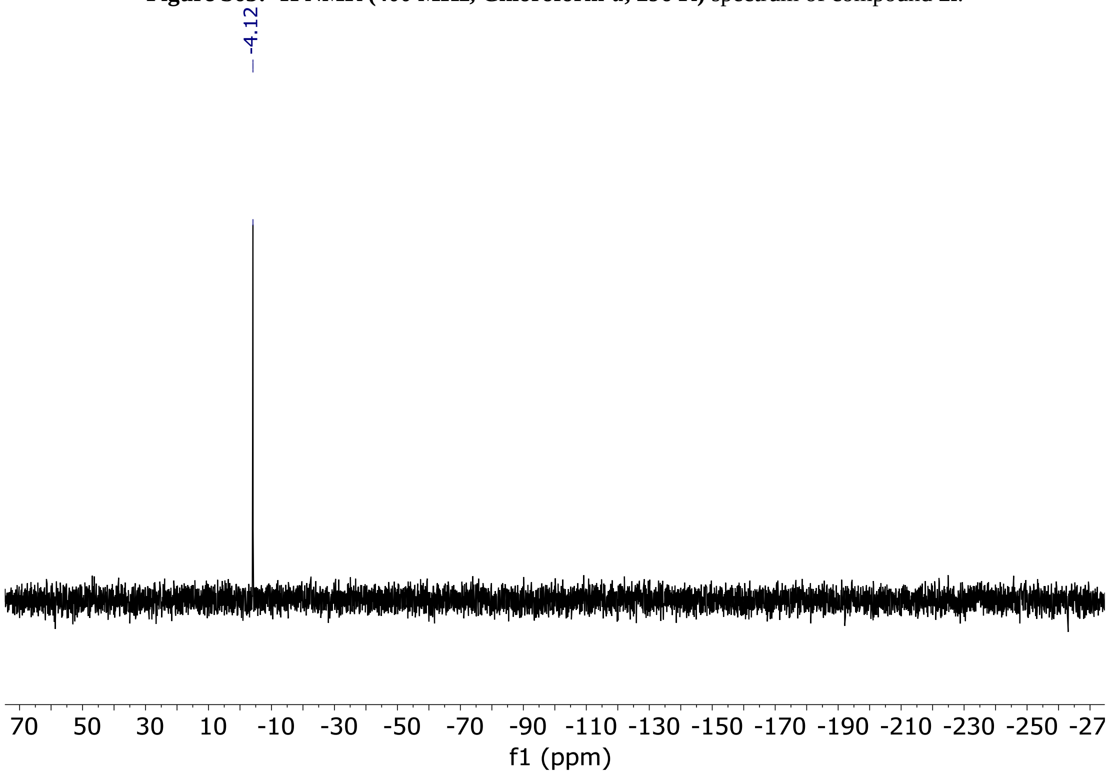

Figure S64: ^{29}Si NMR (79 MHz, Chloroform-*d*, 296K) spectrum of compound **2l**.

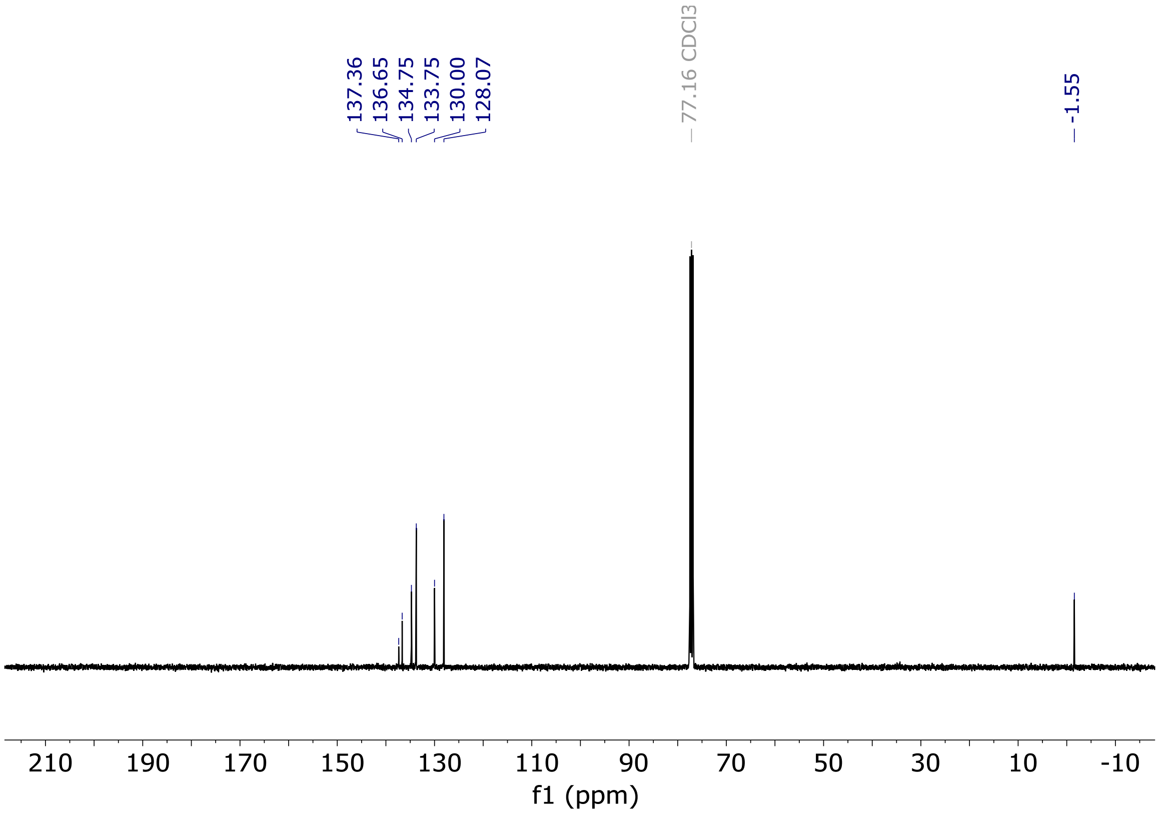

Figure S65: $^{13}C\{^{1}H\}$ NMR (101 MHz, Chloroform-*d*, 296 K) spectrum of compound **2l**.

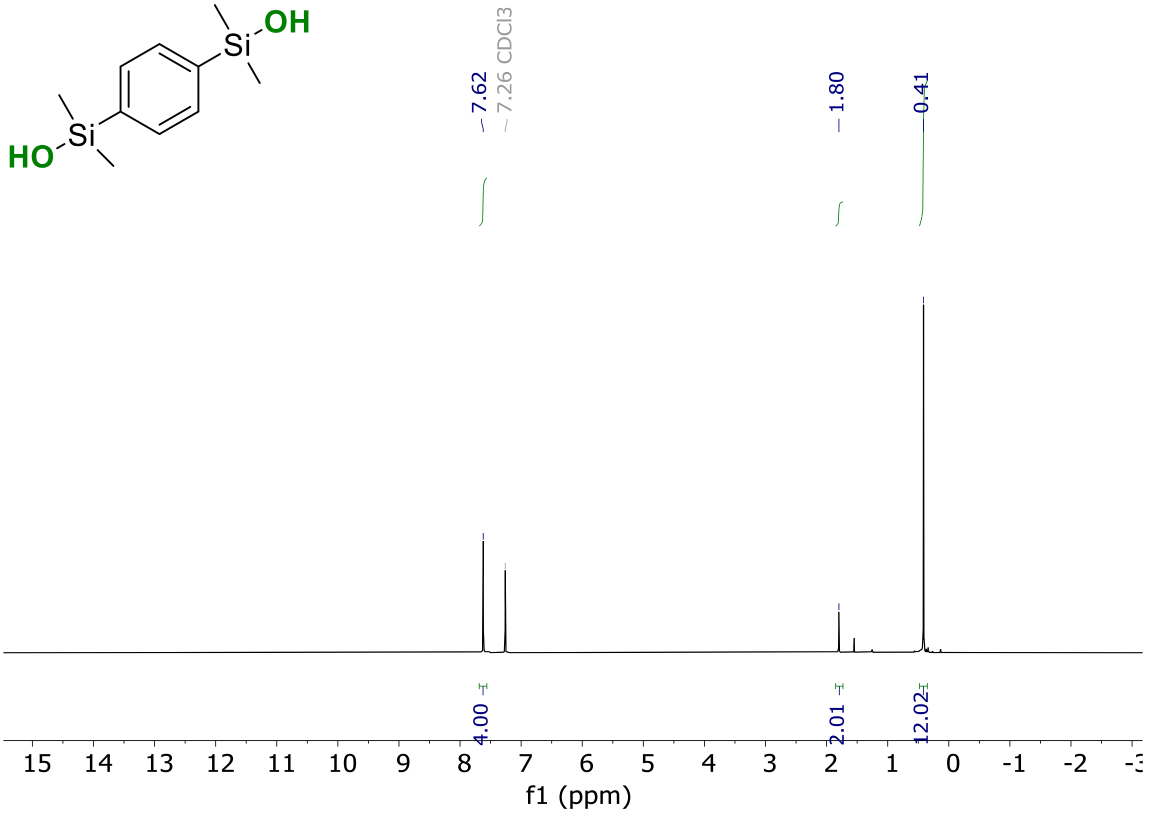

Figure S66: ^{1}H NMR (400 MHz, Chloroform-*d*, 296 K) spectrum of compound **2n**.

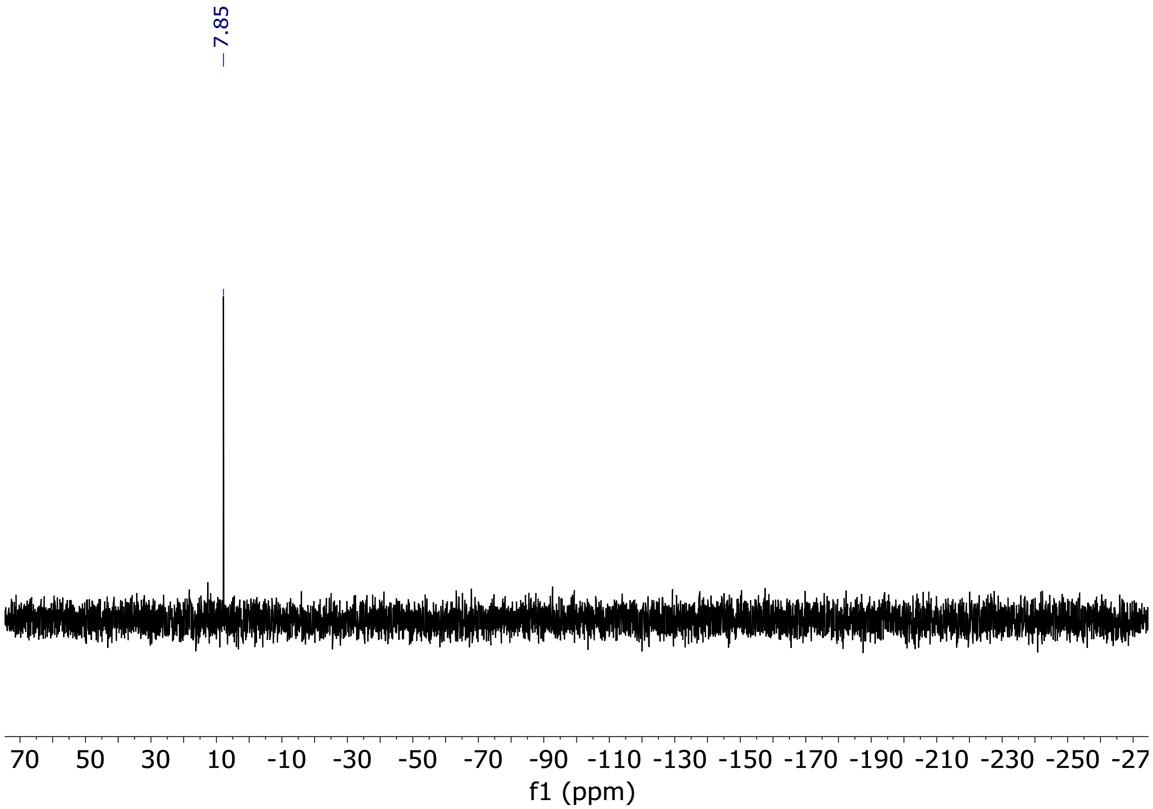

Figure S67: ^{29}Si NMR (79 MHz, Chloroform-*d*, 296K) spectrum of compound **2n**.

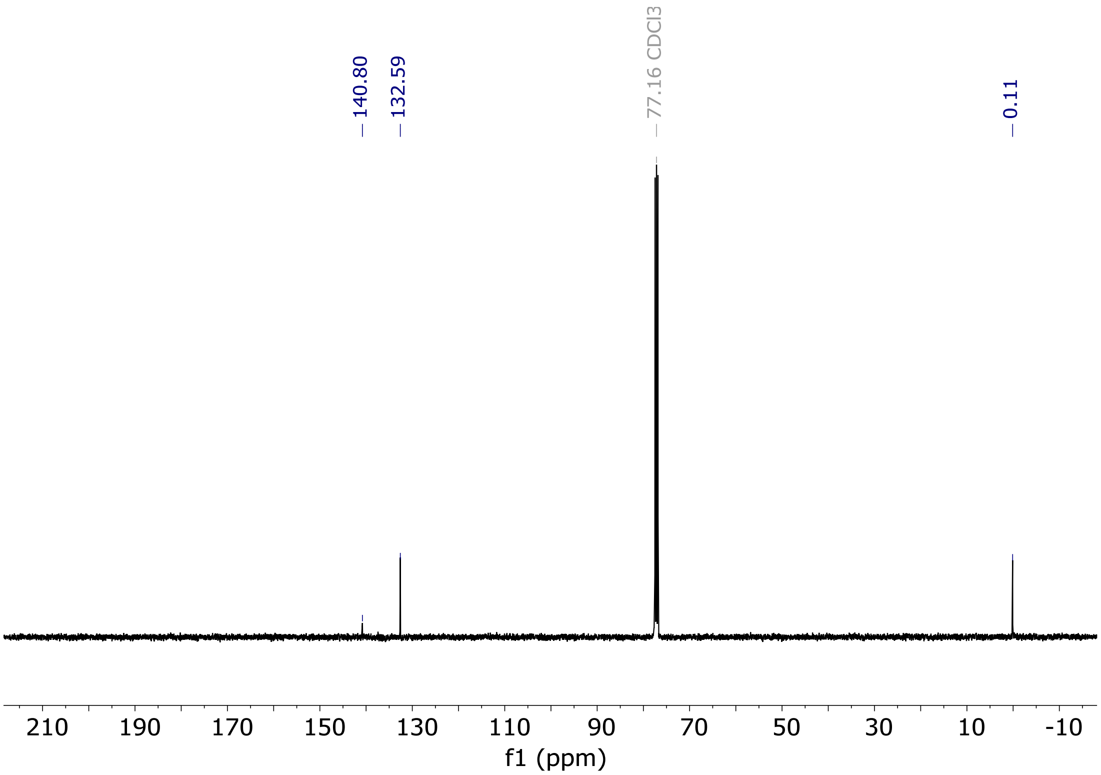

Figure S68: ^{13}C{^{1}H} NMR (101 MHz, Chloroform-*d*, 296 K) spectrum of compound **2n**.

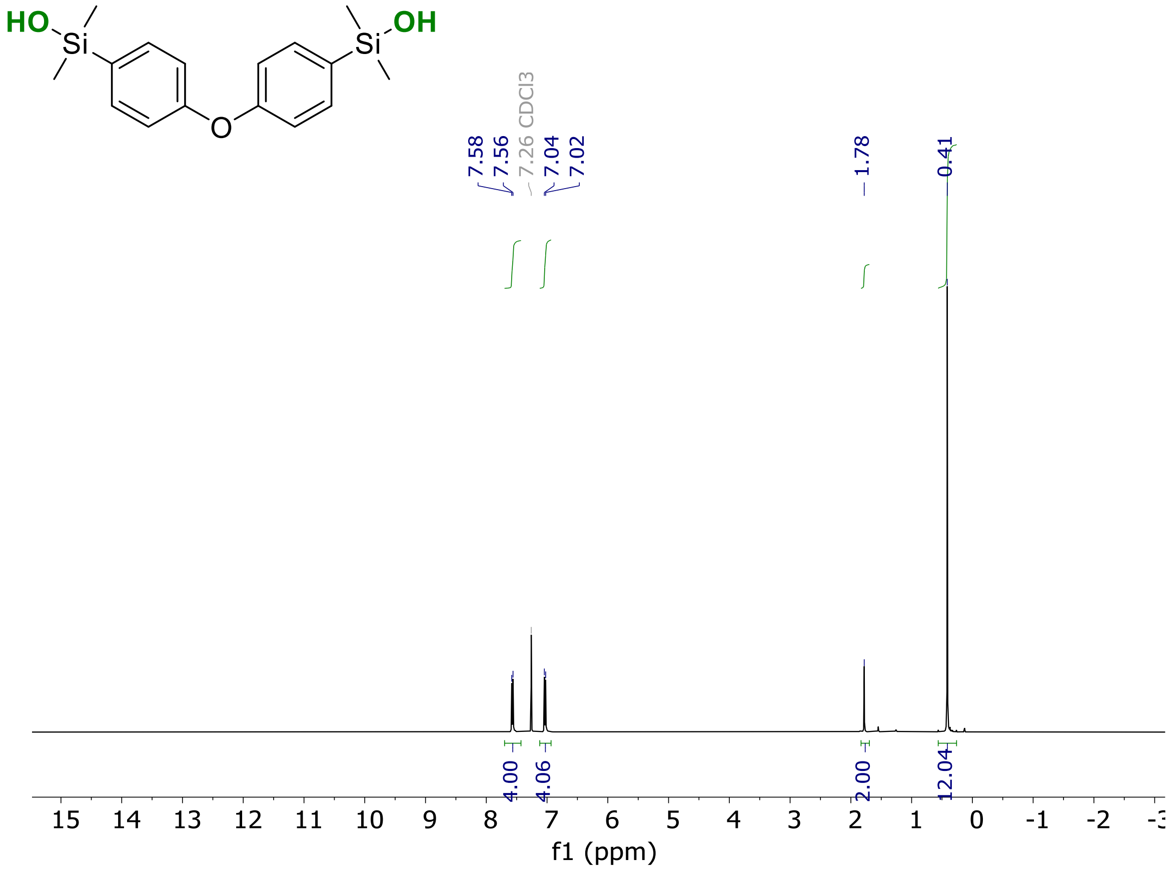

Figure S69: ¹H NMR (400 MHz, Chloroform-*d*, 296 K) spectrum of compound **2o**.

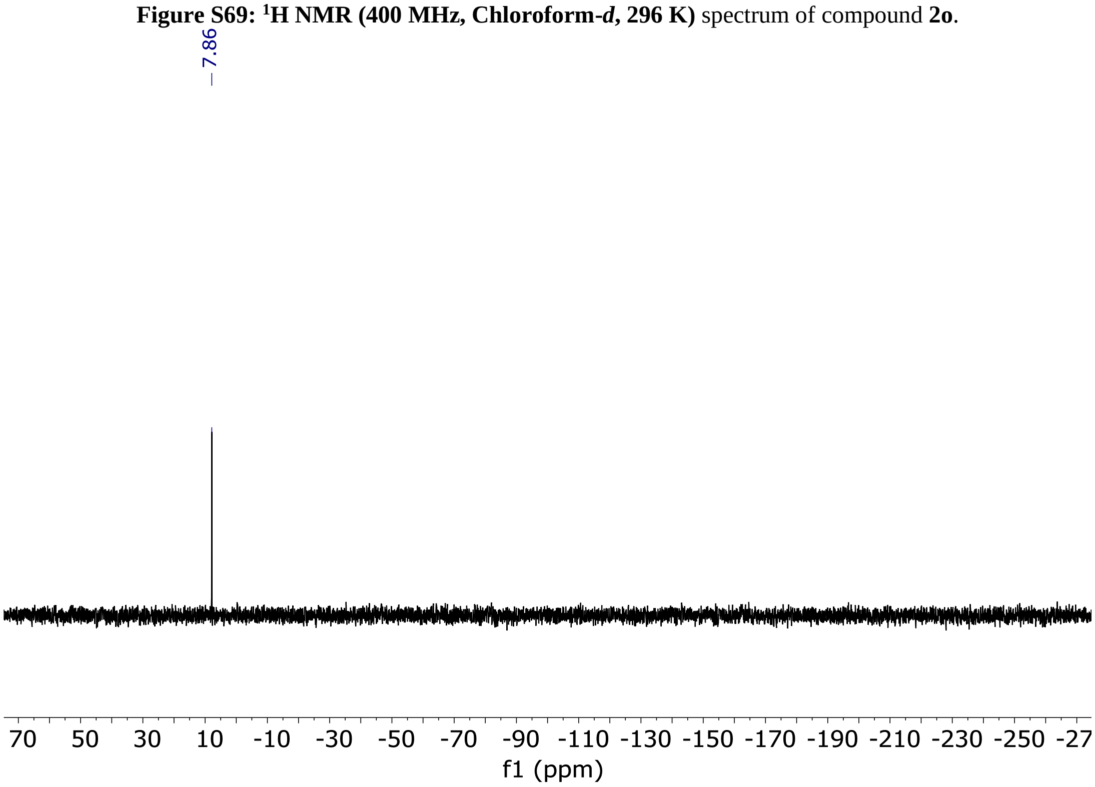

Figure S70: ²⁹Si NMR (79 MHz, Chloroform-*d*, 296K) spectrum of compound **2o**.

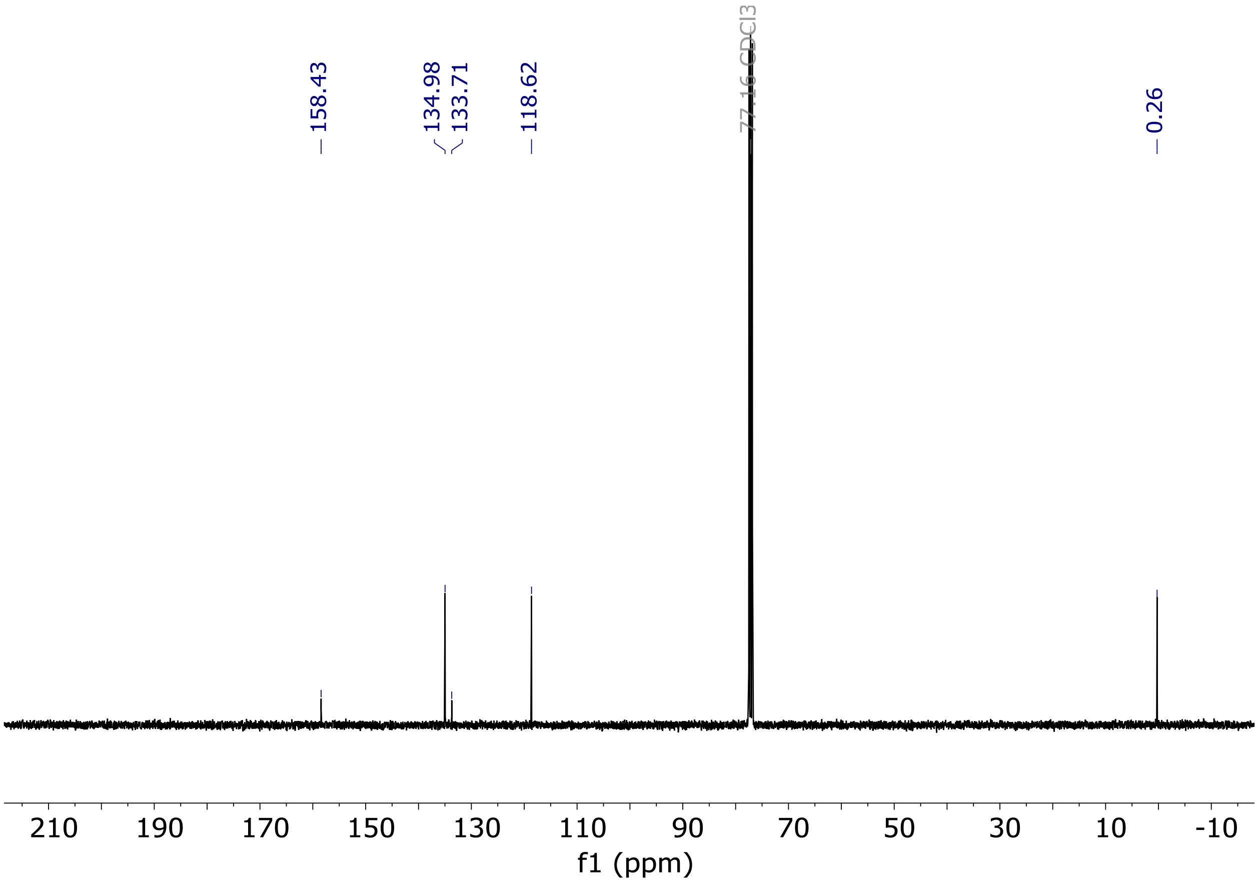

Figure S71: $^{13}C\{^1H\}$ NMR (101 MHz, Chloroform-*d*, 296 K) spectrum of compound **2o**.

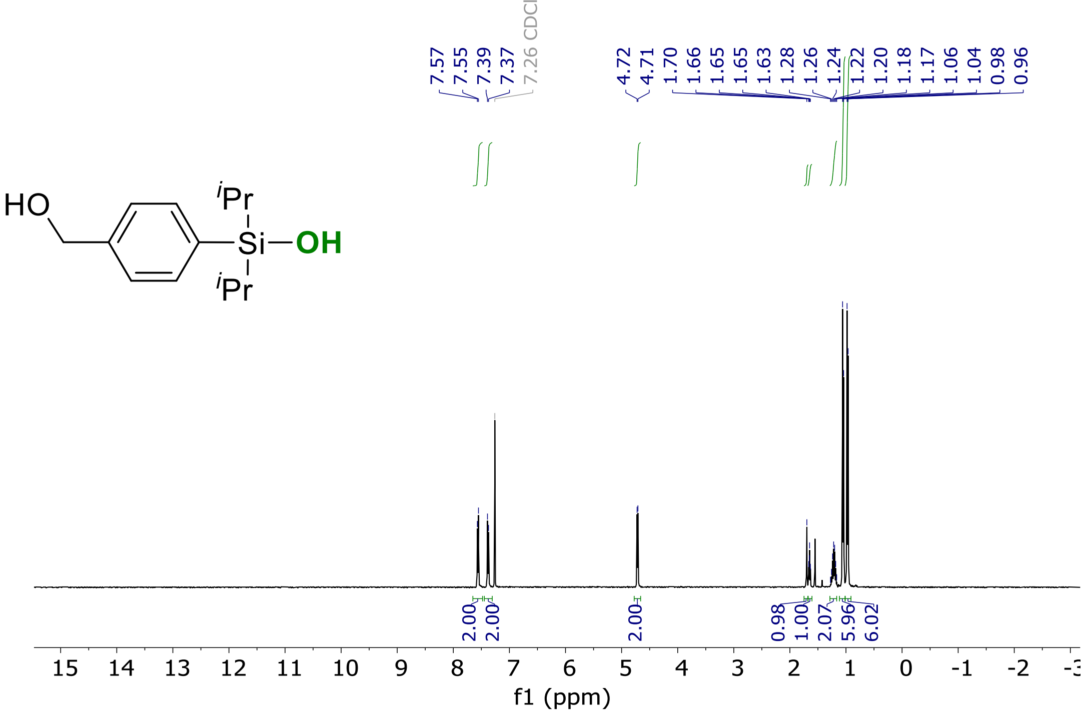

Figure S72: ^1H NMR (400 MHz, Chloroform-*d*, 296 K) spectrum of compound **2p**.

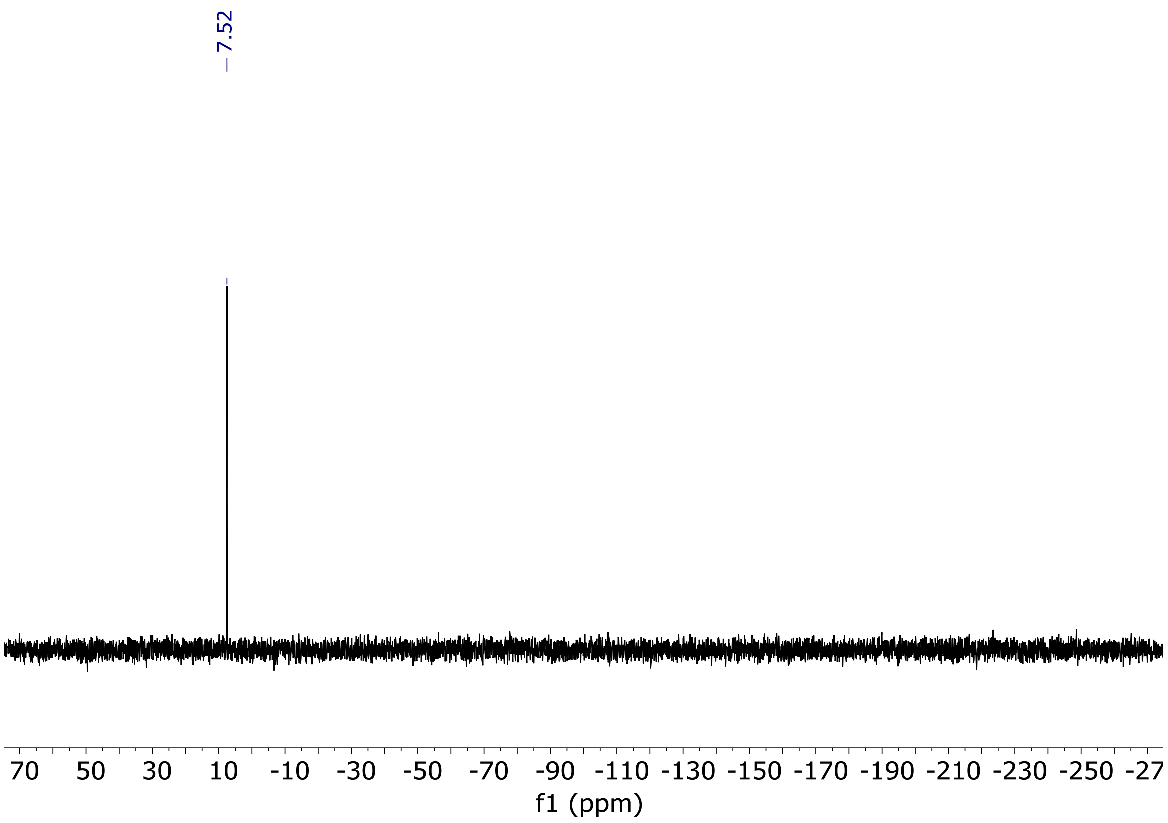

Figure S73: ^{29}Si NMR (79 MHz, Chloroform-*d*, 296K) spectrum of compound **2p**.

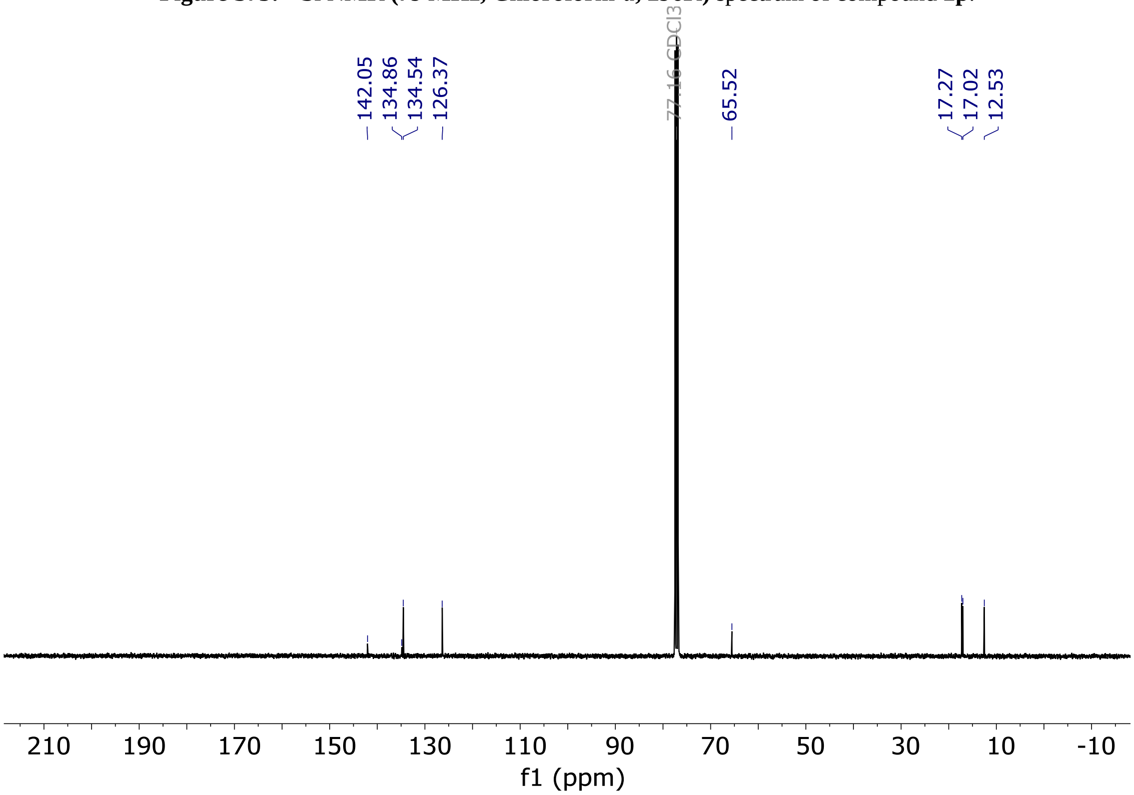

Figure S74: ^{13}C{^{1}H} NMR (101 MHz, Chloroform-*d*, 296 K) spectrum of compound **2p**.

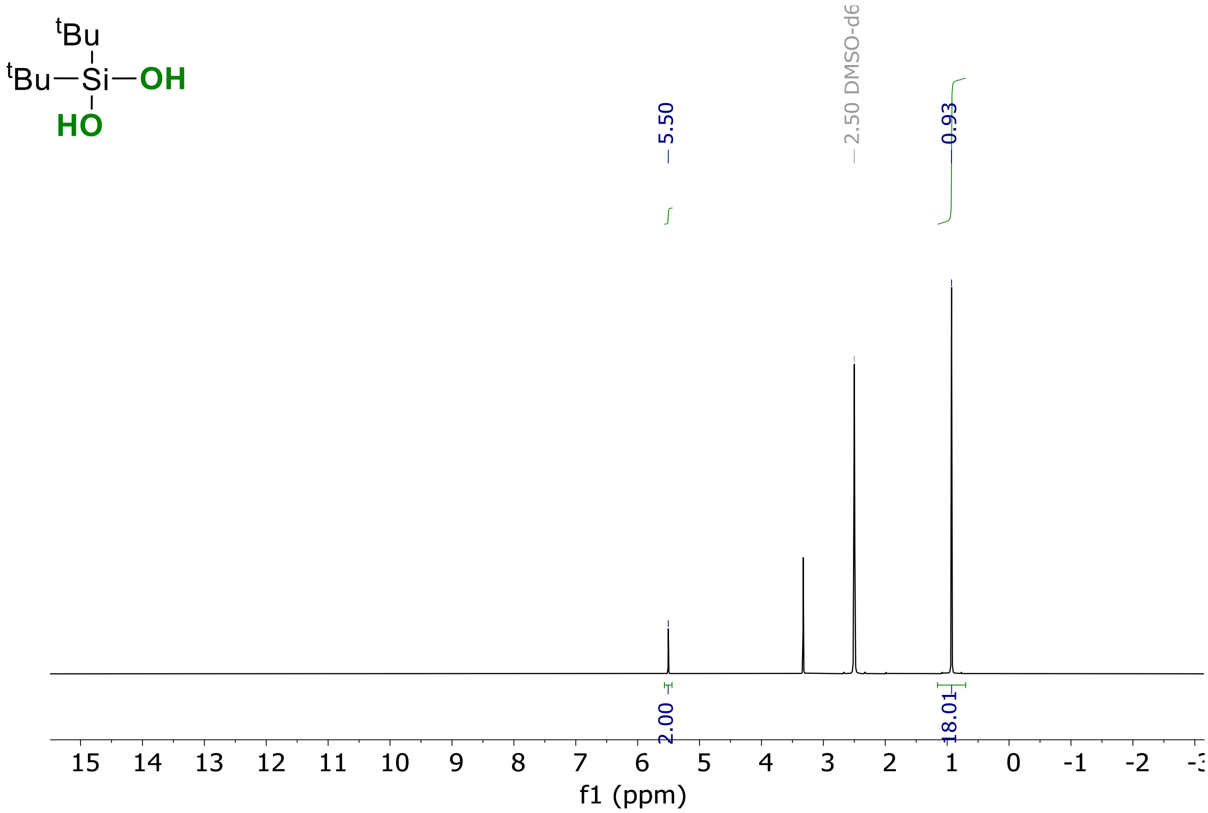

Figure S75: ^{1}H NMR (400 MHz, DMSO-*d*$_6$, 296 K) spectrum of compound **2q**.

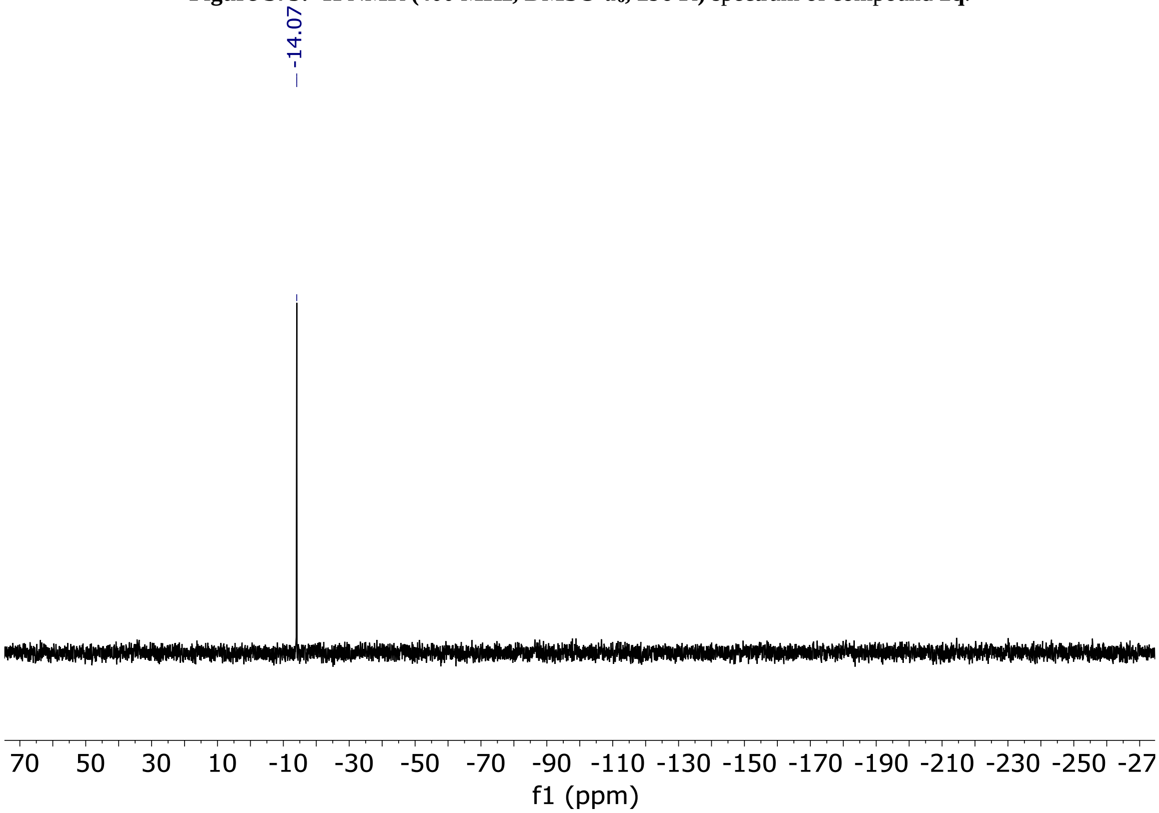

Figure S76: ^{29}Si NMR (79 MHz, DMSO-*d*$_6$, 296 K) spectrum of compound **2q**.

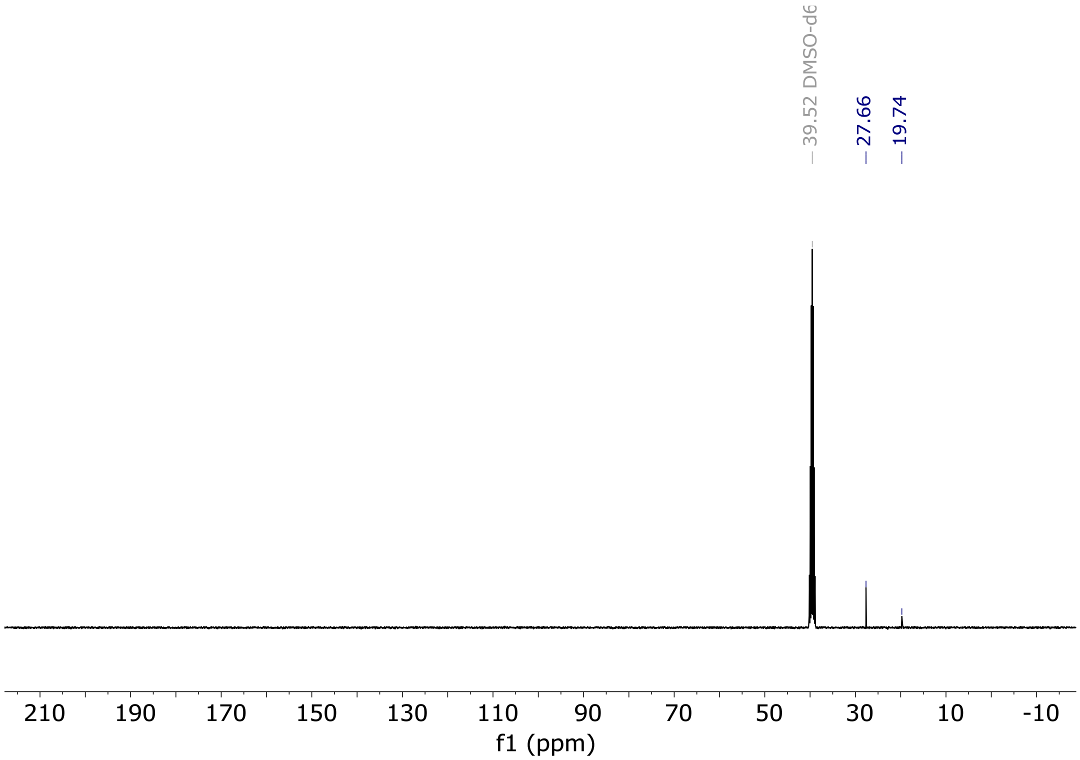

Figure S77: $^{13}C\{^{1}H\}$ NMR (101 MHz, DMSO-d_6, 296 K) spectrum of compound **2q**.

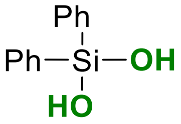

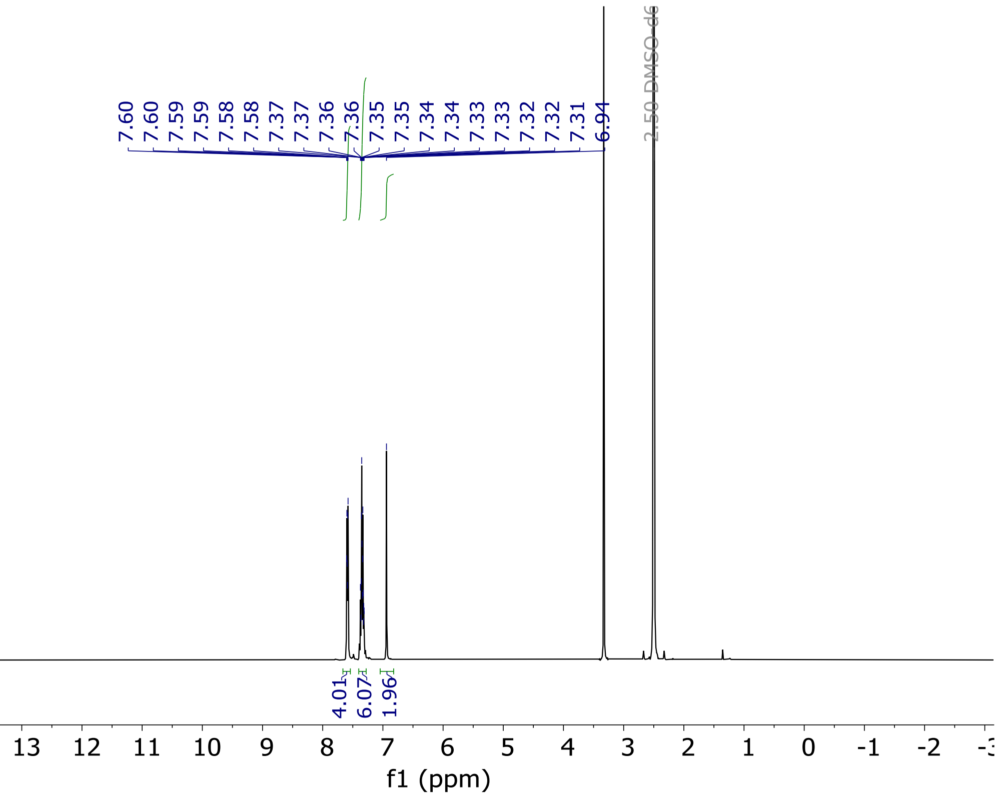

Figure S78: ^{1}H NMR (400 MHz, DMSO-d_6, 296 K) spectrum of compound **2r**.

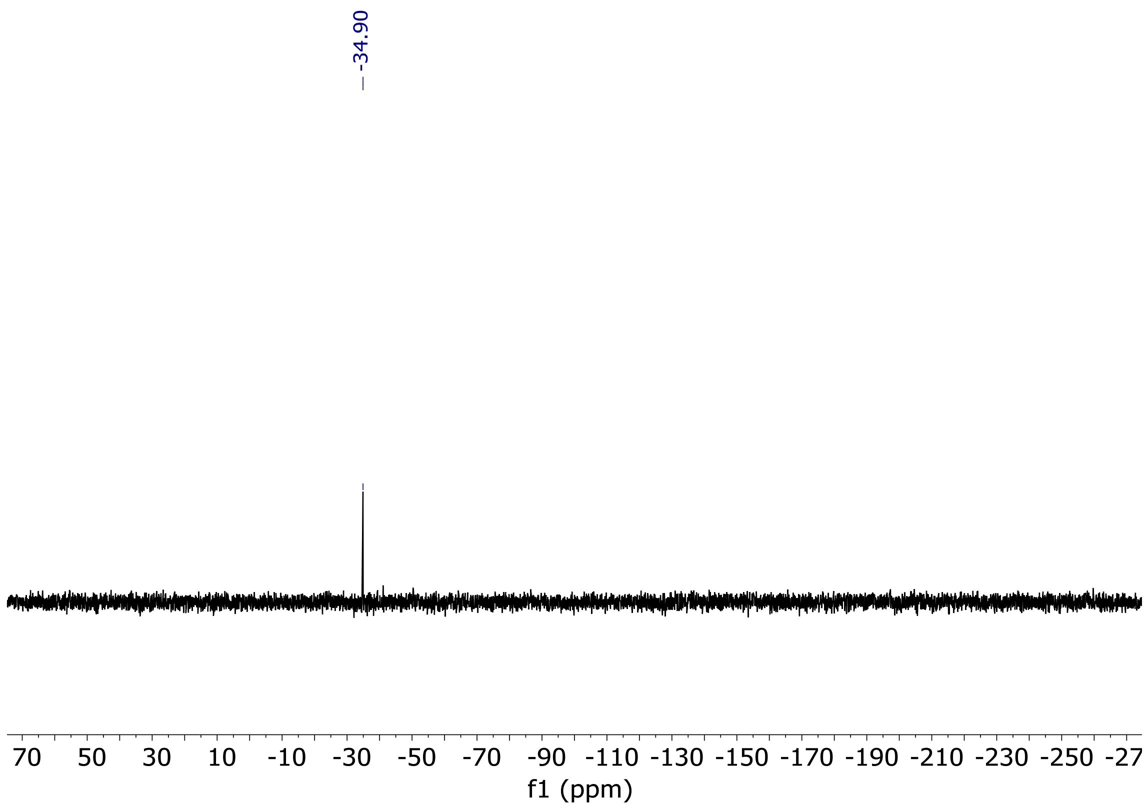

Figure S79: ^{29}Si NMR (79 MHz, DMSO-d_6, 296 K) spectrum of compound **2r**.

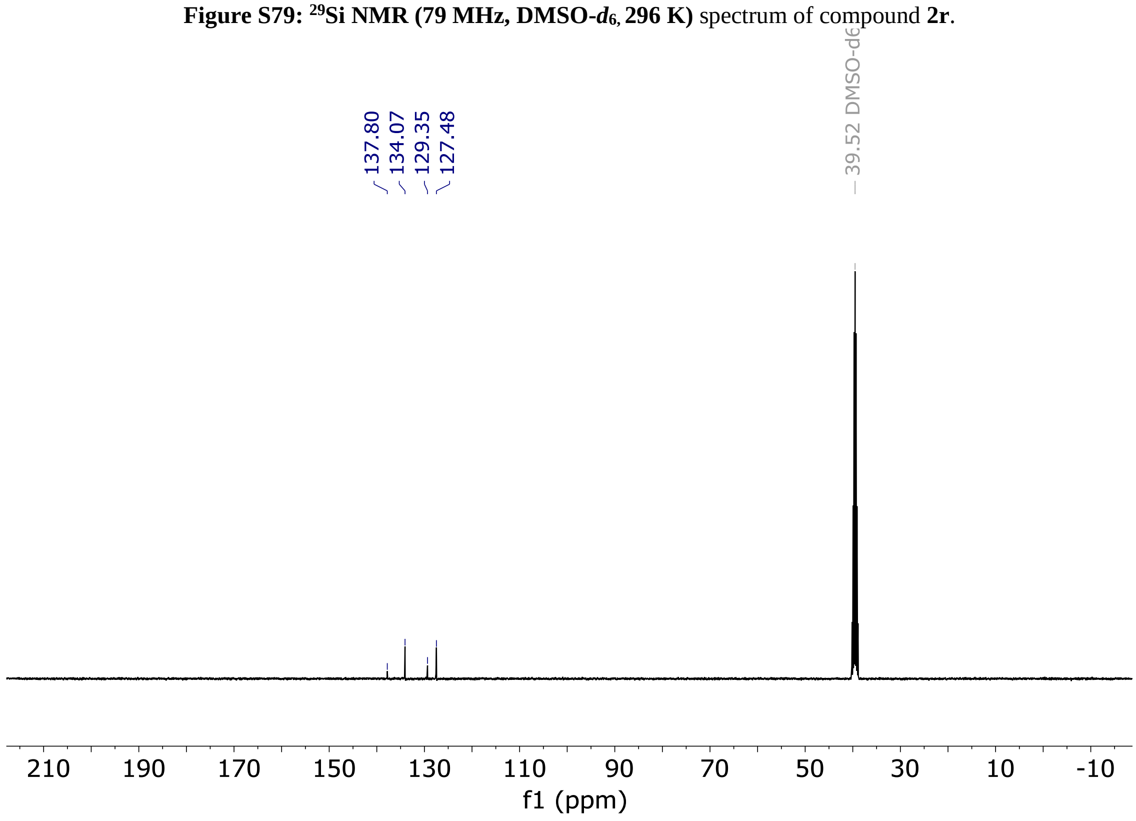

Figure S80: ^{13}C{^{1}H} NMR (101 MHz, DMSO-d_6, 296 K) spectrum of compound **2r**.

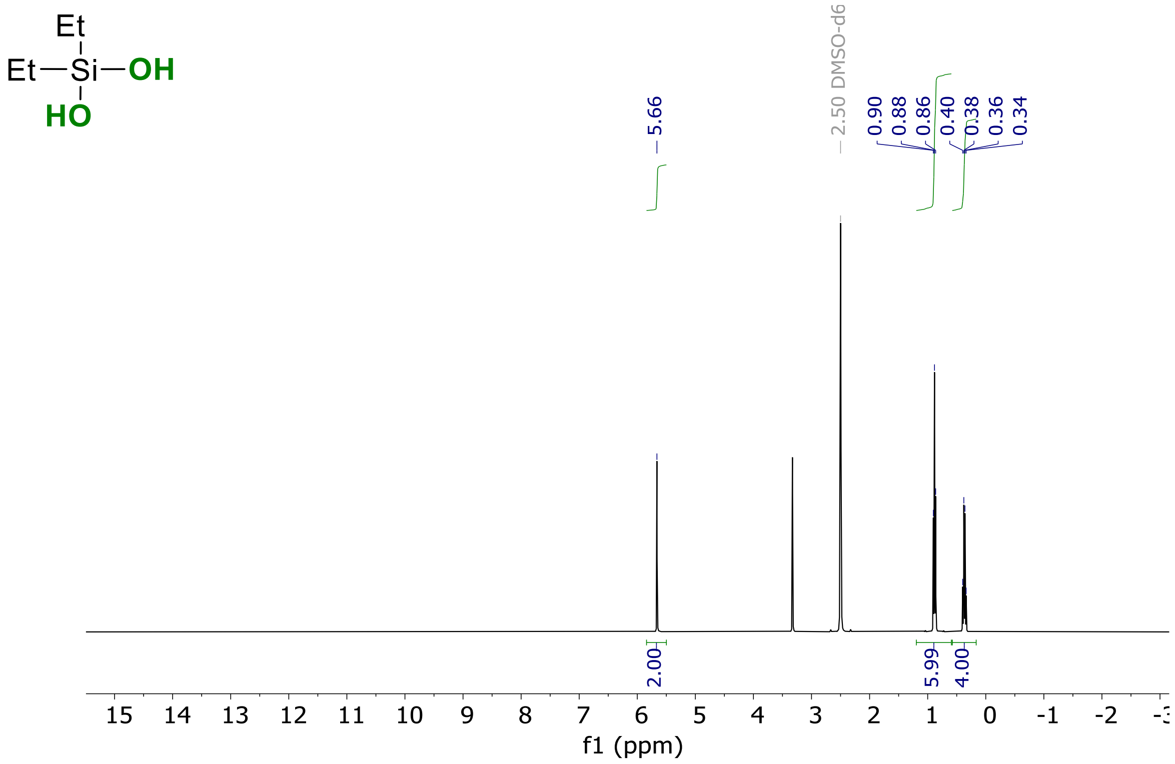

Figure S81: ^{1}H NMR (400 MHz, DMSO-d_6, 296 K) spectrum of compound **2s**.

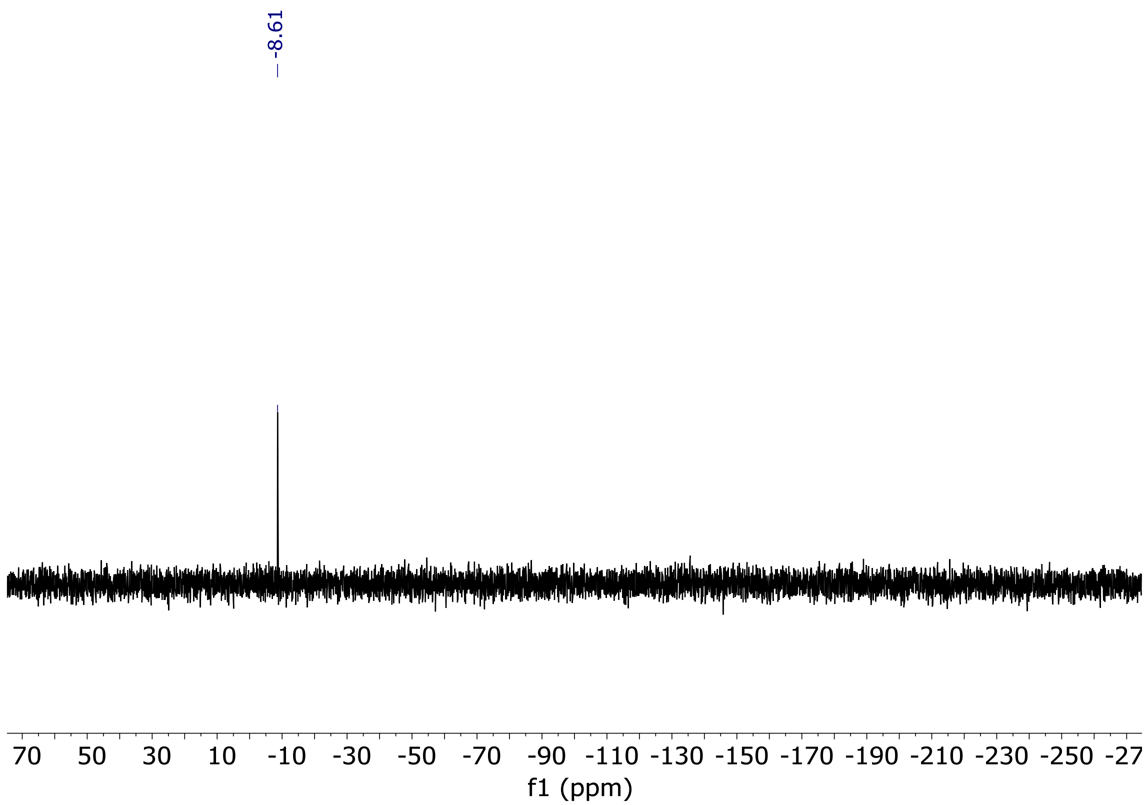

Figure S82: ^{29}Si NMR (79 MHz, DMSO-d_6, 296 K) spectrum of compound **2s**.

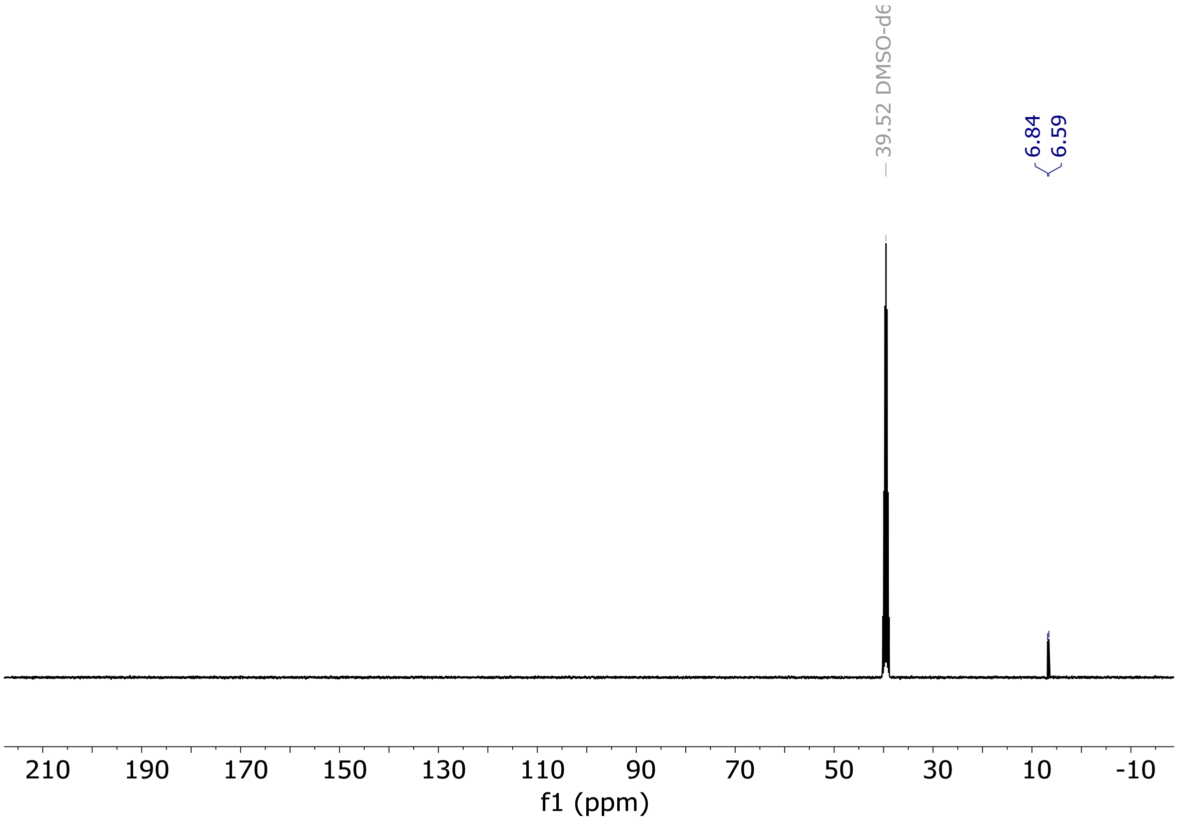

Figure S83: $^{13}C\{^{1}H\}$ NMR (101 MHz, DMSO-d_6, 296 K) spectrum of compound **2s**.

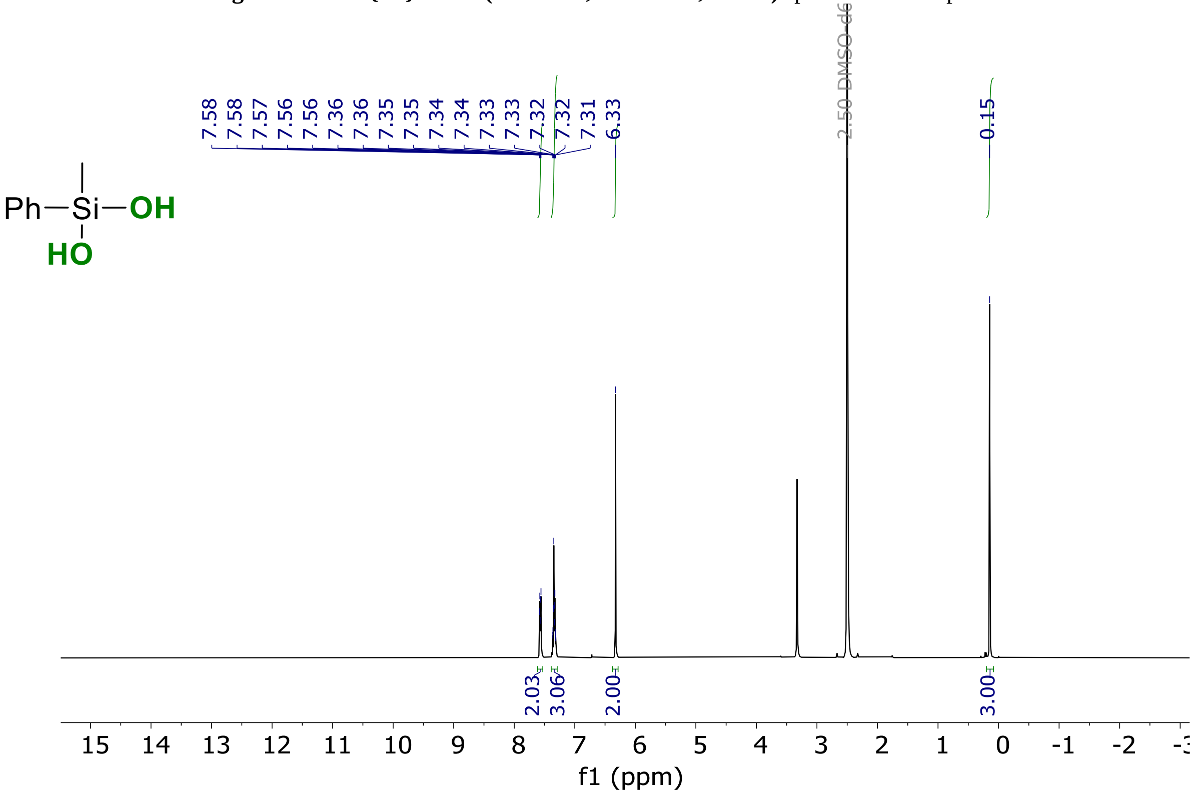

Figure S84: ^{1}H NMR (400 MHz, DMSO-d_6, 296 K) spectrum of compound **2t**.

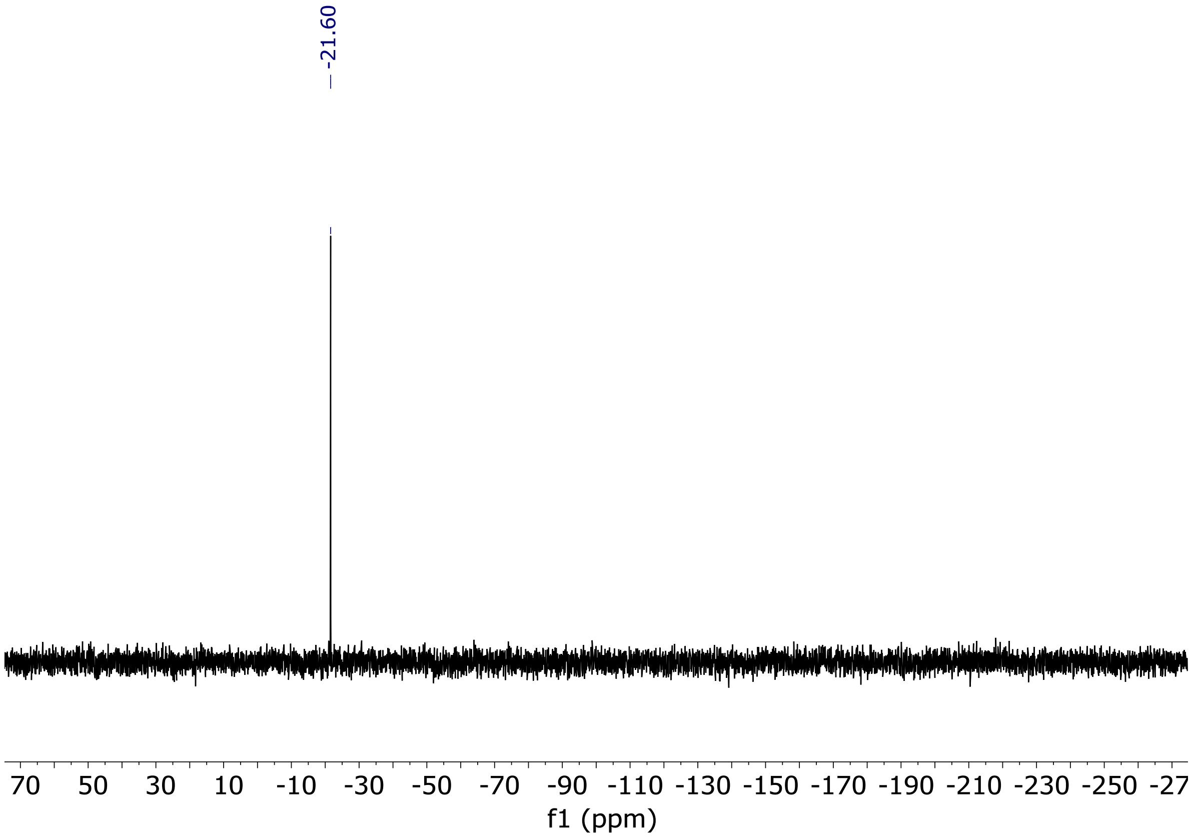

Figure S85: ^{29}Si NMR (79 MHz, DMSO-*d*$_6$, 296 K) spectrum of compound **2t**.

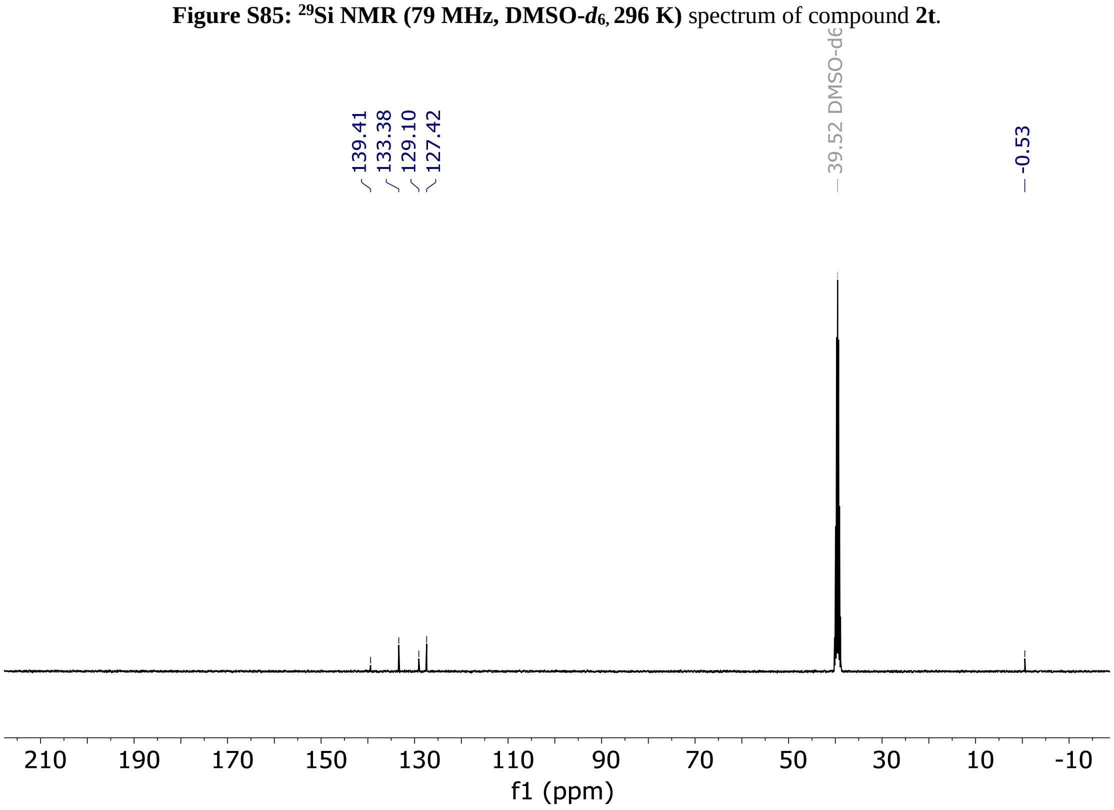

Figure S86: ^{13}C{^{1}H} NMR (101 MHz, DMSO-*d*$_6$, 296 K) spectrum of compound **2t**.

12.3. Failed substrates

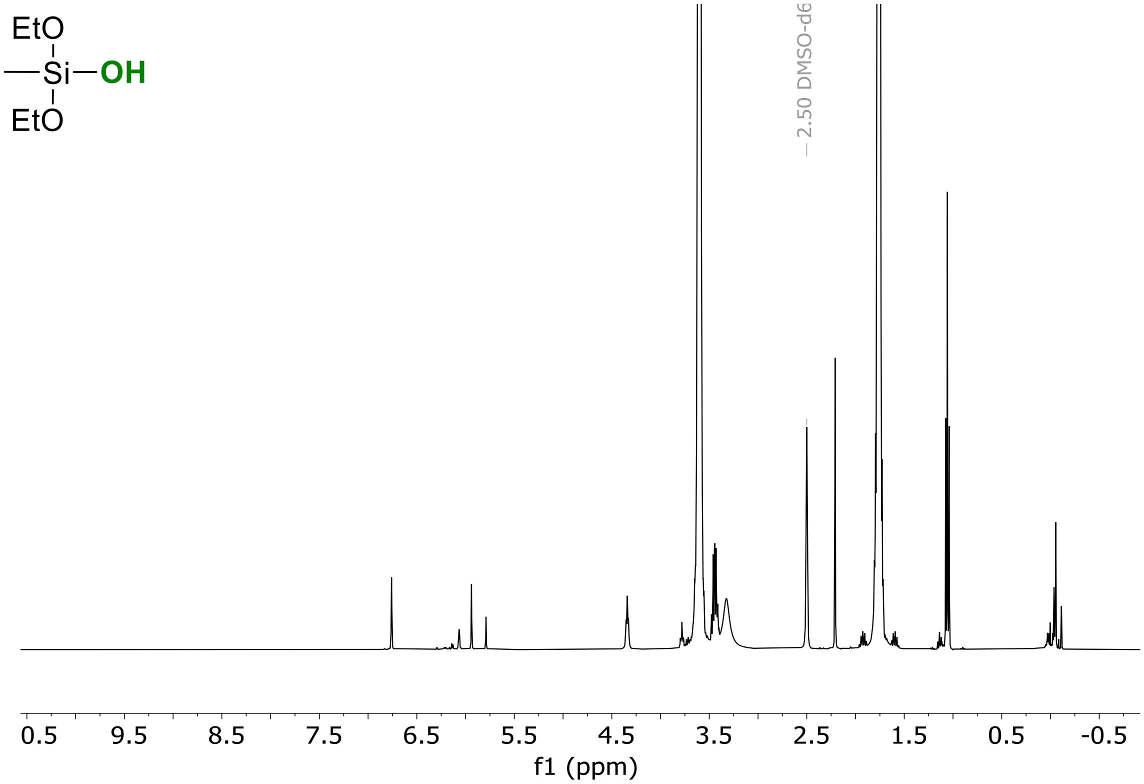

Figure S87: ^{1}H NMR (400 MHz, DMSO-*d$_6$* 298.1 K) spectrum of the crude reaction mixture of silane **2u** in the conditions reported in Section 3.5.

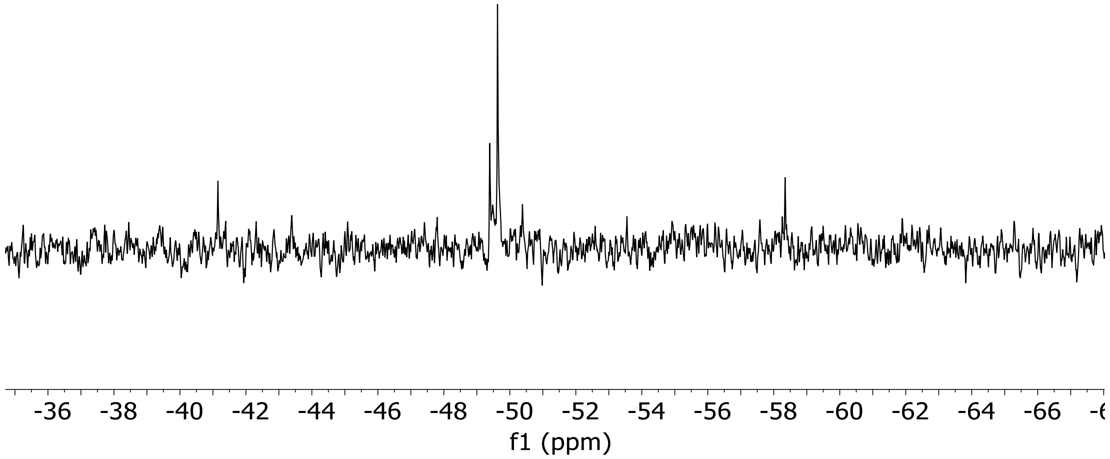

Figure S88: ^{29}Si NMR (79 MHz, DMSO-*d$_6$*, 296 K) spectrum of the crude reaction mixture of silane **2u** in the conditions reported in Section 3.5.

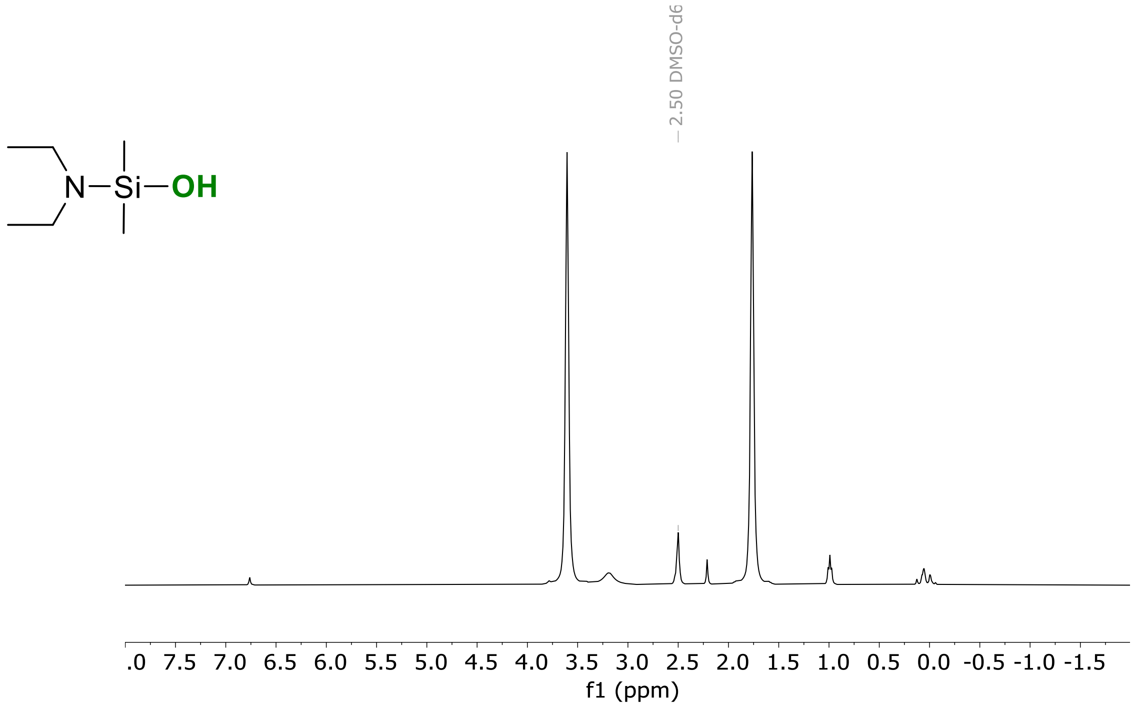

Figure S89: ^{1}H NMR (400 MHz, DMSO-*d₆* 298.1 K) spectrum of the crude reaction mixture of silane **2v** in the conditions reported in Section 3.5.

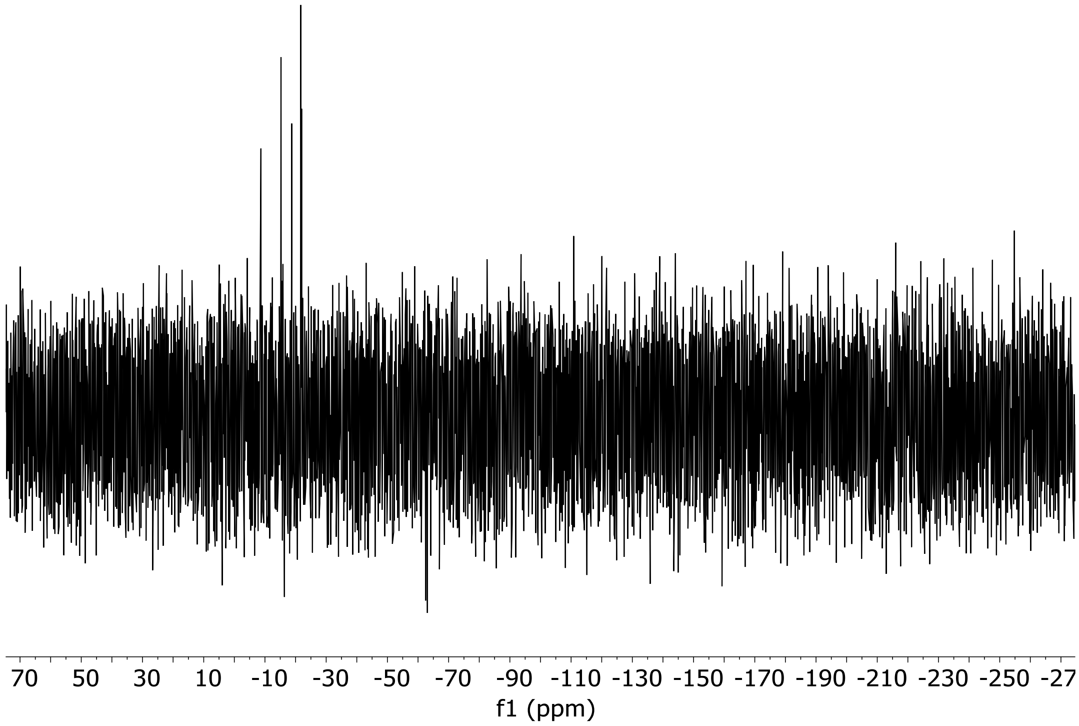

Figure S90: ^{29}Si NMR (79 MHz, DMSO-d_6, 296 K) spectrum of the crude reaction mixture of silane **2v** in the conditions reported in Section 3.5.

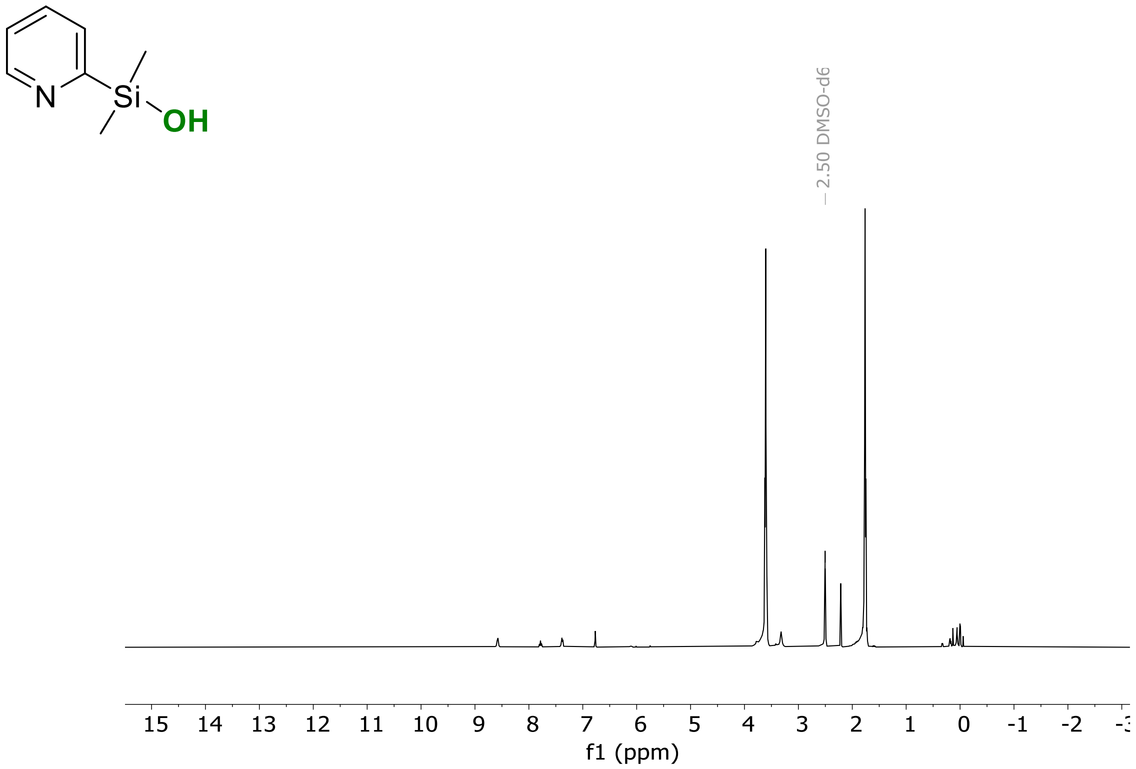

Figure S91: ^{1}H NMR (400 MHz, DMSO-*d*$_6$, 298.1 K) spectrum of the crude reaction mixture of silane **2z** in the conditions reported in Section 3.5.

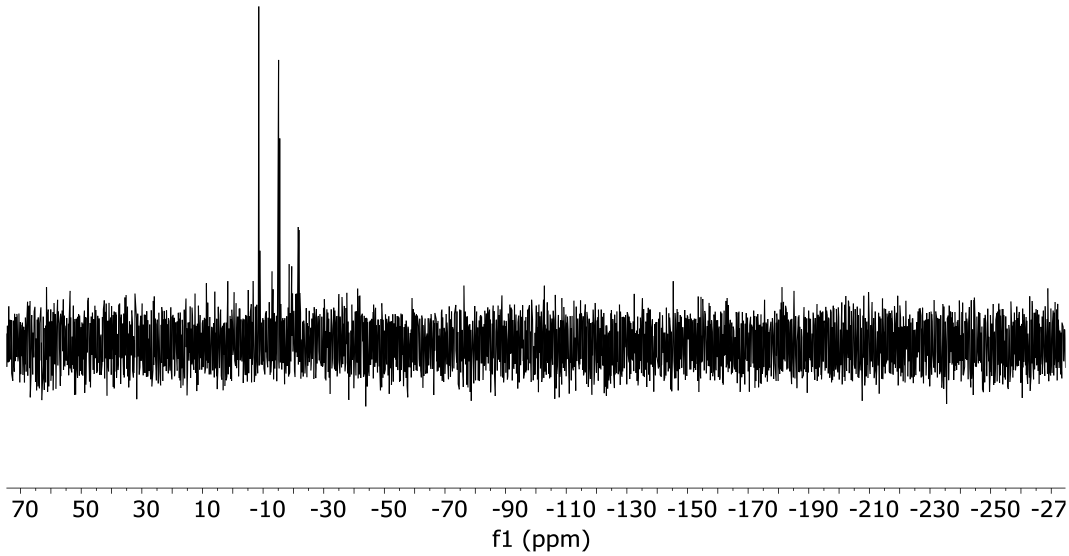

Figure S92: ^{29}Si NMR (79 MHz, DMSO-*d*$_6$, 296 K) spectrum of the crude reaction mixture of silane **2z** in the conditions reported in Section 3.5.

12.4. Gram scale reaction

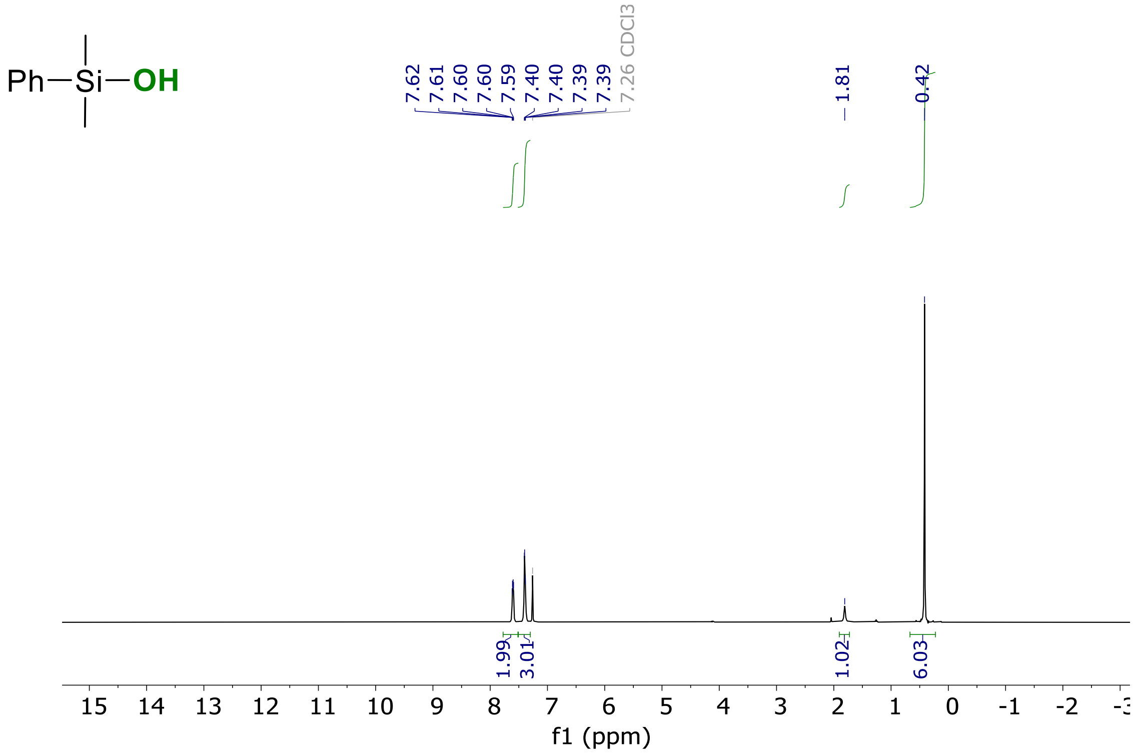

Figure S93: ¹H NMR (400 MHz, Chloroform-*d*, 296 K) spectrum of compound **2a** obtained from a preparative scale reaction.

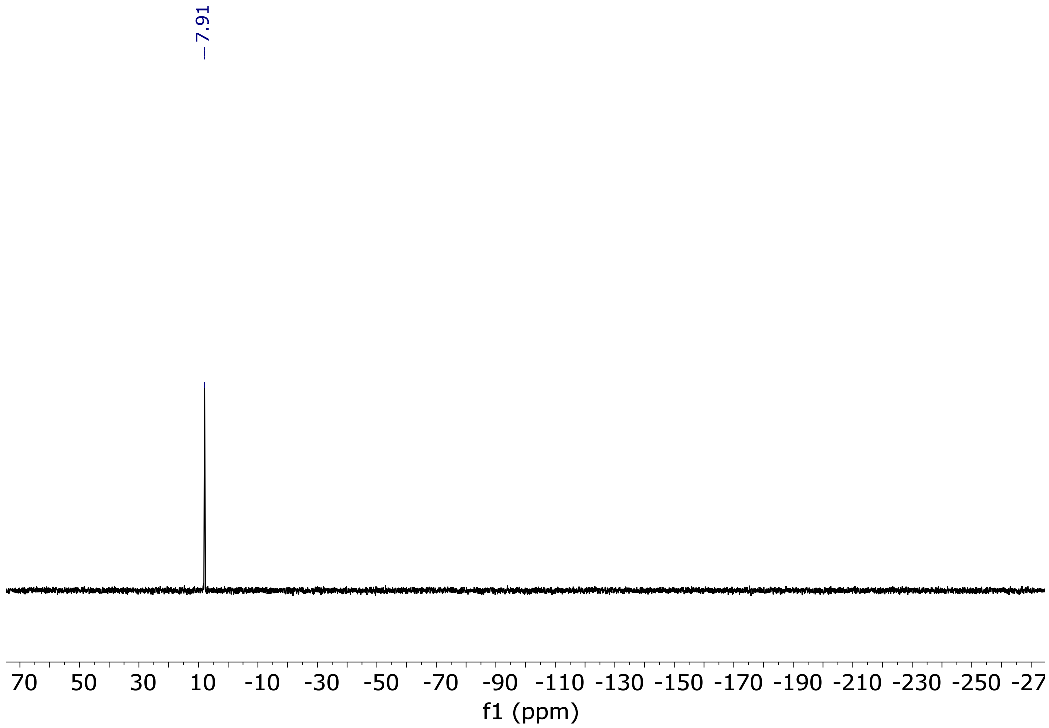

Figure S94: ²⁹Si NMR (79 MHz, Chloroform-*d*, 296 K) spectrum of compound **2a** obtained from a preparative scale reaction.

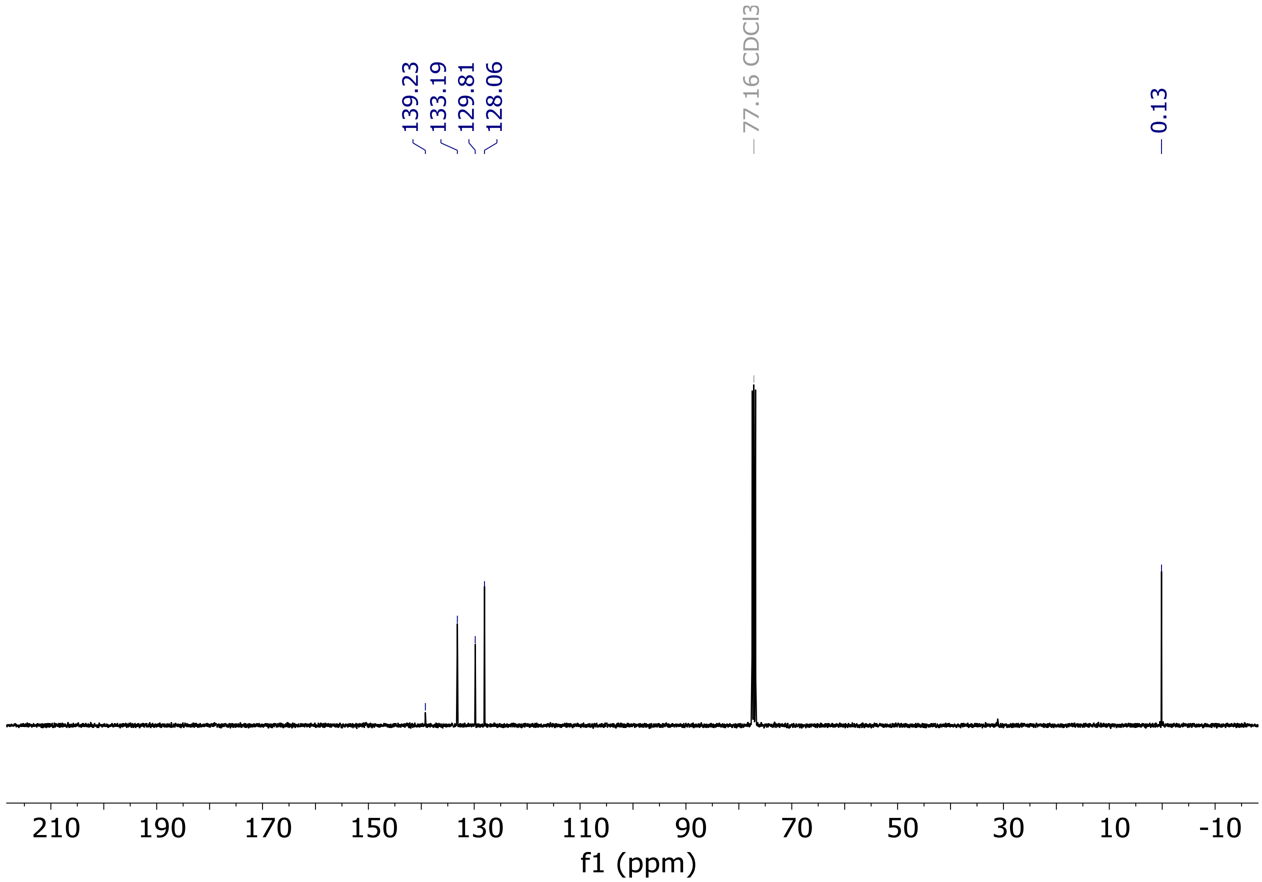

Figure S95: ^{13}C{^{1}H} NMR (101 MHz, Chloroform-*d*, 296 K) spectrum of compound **2a** obtained from a preparative scale reaction.

12.5. $PhMe_2SiD$

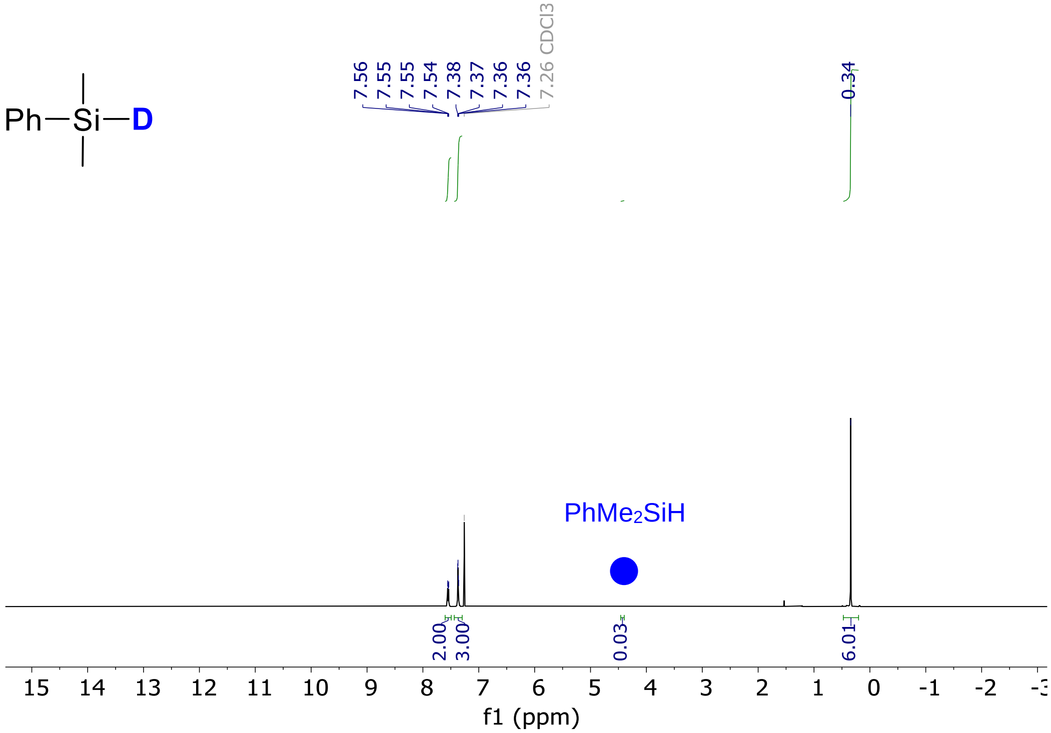

Figure S96: ^{1}H NMR (400 MHz, Chloroform-*d*, 296 K) spectrum of $PhMe_2SiD$.

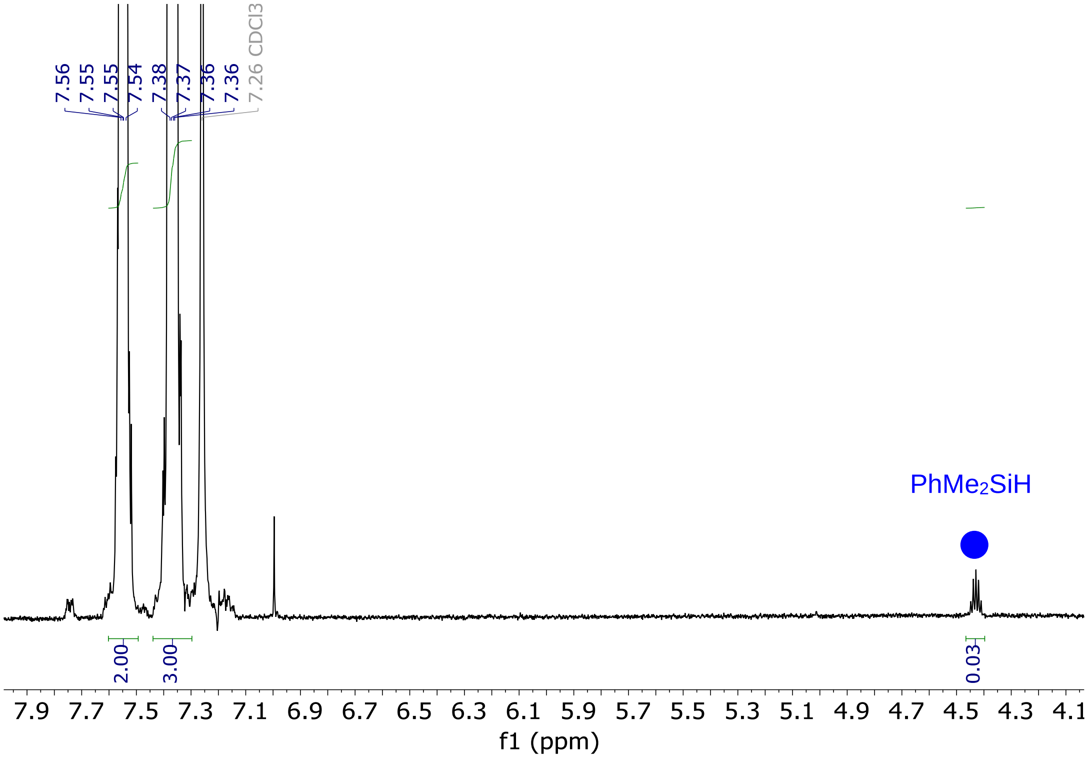

Figure S97: ^{1}H NMR (400 MHz, Chloroform-*d*, 296 K) spectrum**.** Detail of the spectrum of $PhMe_2SiD$.

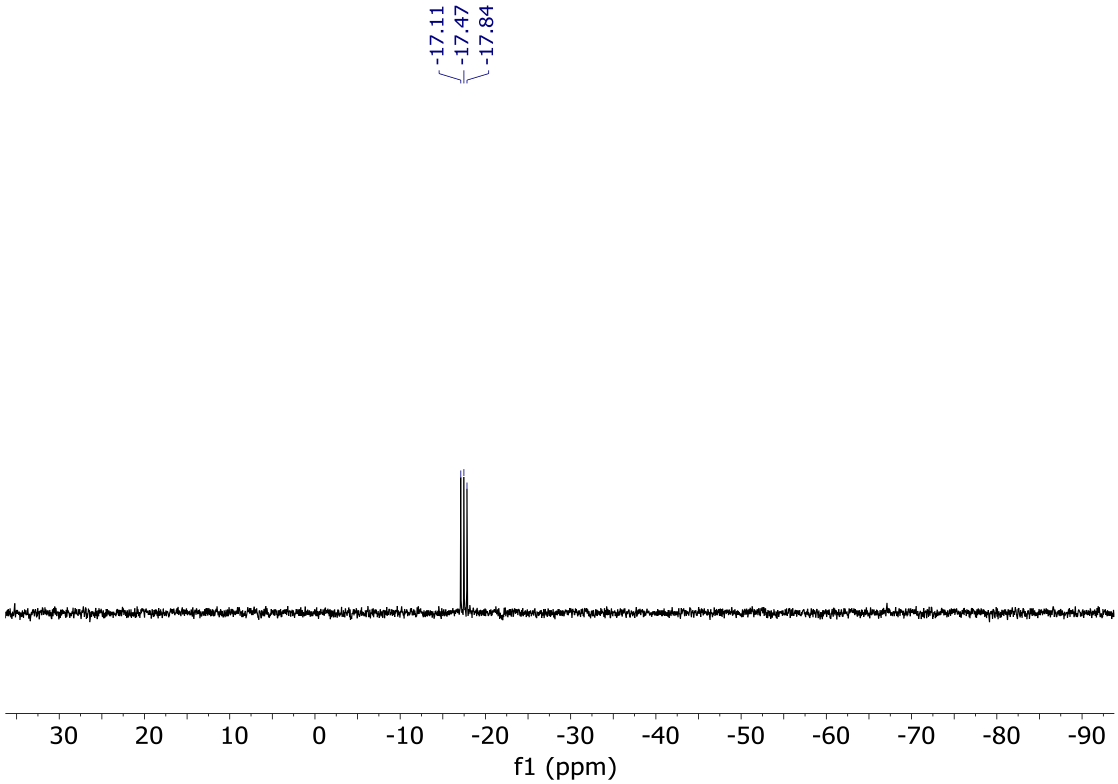

Figure S98: ^{29}Si NMR (79 MHz, Chloroform-*d*, 296 K) spectrum of $PhMe_2SiD$.

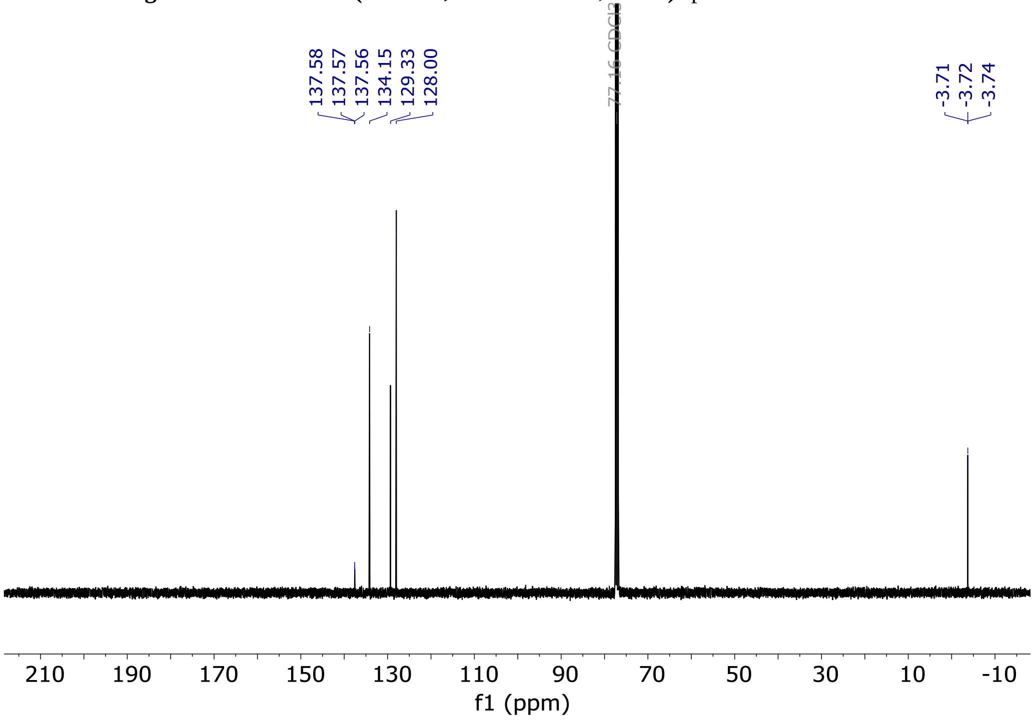

Figure S99: ^{13}C NMR (101 MHz, Chloroform-*d*, 296 K) spectrum of $PhMe_2SiD$.

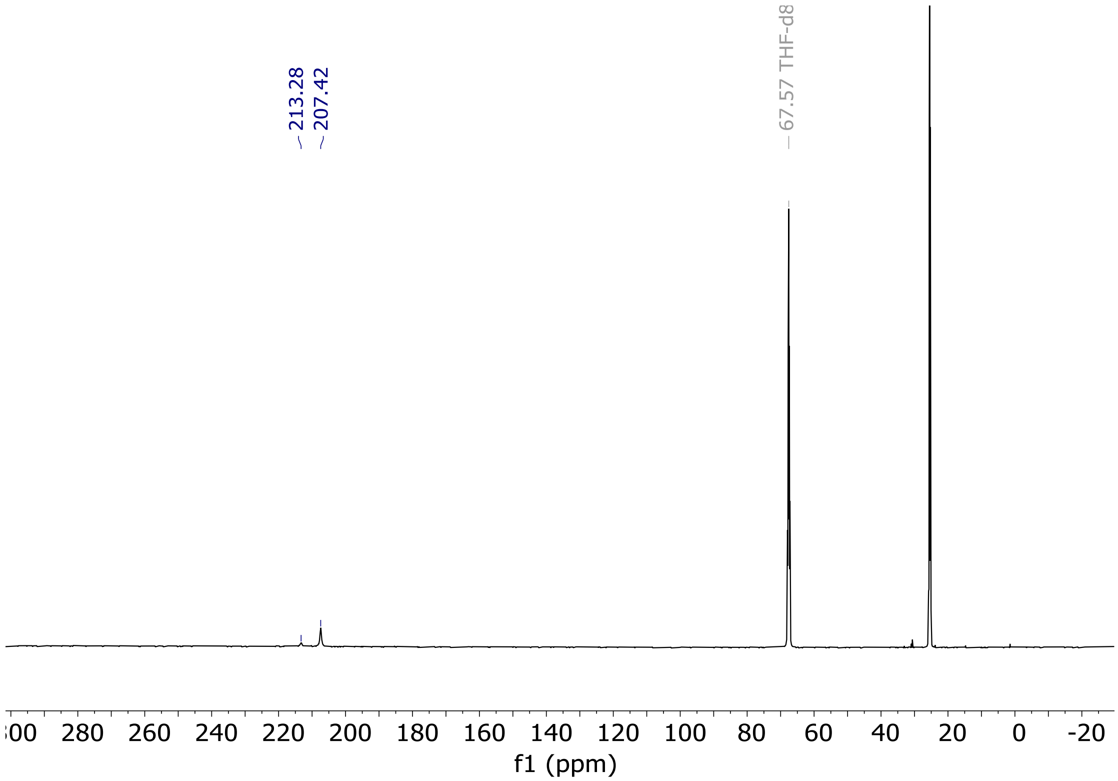

Figure S100: ^{13}C NMR (101 MHz, THF-d_8, 238 K) spectrum of [MnBr(^{13}CO)$_n$(CO)$_{5-n}$].

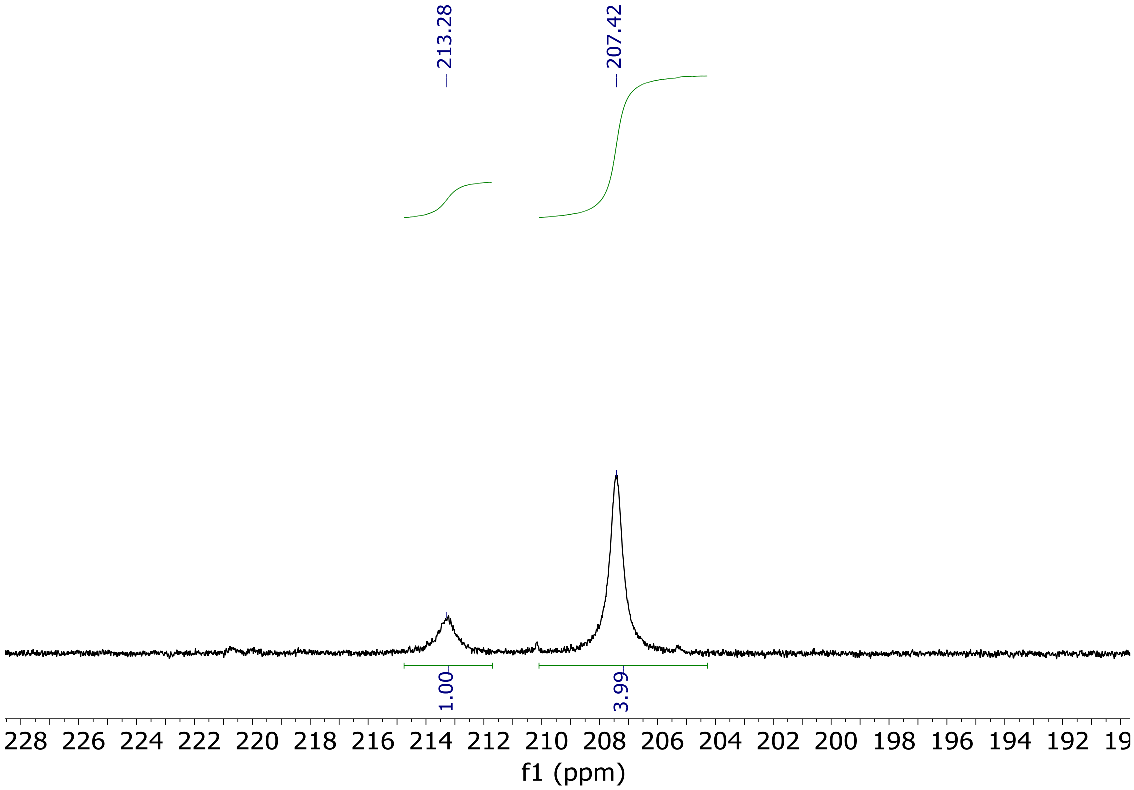

Figure S101: ^{13}C NMR (101 MHz, THF-d_8, 238 K) spectrum. Detail of the spectrum of [MnBr(^{13}CO)$_n$(CO)$_{5-n}$].

13. X-ray Crystallography and Refinement of Structures

Two colorless single crystals of dimensions 0.26 × 0.07 × 0.07 mm^3 (CCDC 2184831, CCDC 2184832) and 0.27 × 0.12 × 0.10 mm^3 (CCDC 2184833) were selected in polarized light under a microscope and covered with polyfluorinated polyether. The crystals were picked with nylon loops and mounted in the nitrogen cold gas stream of the diffractometer at 100 K. A Bruker D8 Venture diffractometer equipped with an IμS3 Diamond Mo-source (Mo-Kα radiation; λ=0.71073 Å) and an IμS3 Cu-source (Co-Kα radiation; λ=1.54178 Å), INCOATEC Helios mirror optics, and Photon III detector was used for data collection. Final cell constants were obtained from least squares fits of setting angles of several thousand strong reflections. Intensity data were corrected for absorption using intensities of redundant reflections using SADABS[12]. The structures were readily solved by Direct methods and subsequent difference Fourier techniques. The Bruker APEX3[13] software package was used to solve and refine the structures. All non-hydrogen atoms were anisotropically refined, and hydrogen atoms were placed at calculated positions and refined as riding atoms with isotropic displacement parameters.

The asymmetric unit of **2o** (CCDC 2190210) contained two crystallographically independent molecules of $C_{16}H_{22}O_3Si_2$ and a severely disordered chloroform molecule. The scattering contributions of the solvent were removed using the Platon/SQUEEZE program package[14] since an attempt to model the disorder was not completely satisfying. A total of 58 electrons in a void volume of 144 Å3 was found, which perfectly corresponds to the calculated number of electrons for chloroform.

Crystallographic details of data collection and structure refinement are shown in Table S13.

Table S13: Crystallographic data of complexes **Mn-5** and silanols **2p** and **2o**.

	**Mn-5**	**2p**	**2o**
**Formula**	$C_{44}H_{68}Br_6Mn_5O_{22}$	$C_{13}H_{22}O_2Si$	$C_{16}H_{22}O_3Si_2$
**M_r in g mol^{-1}**	1703.14	238.39	318.52
**Colour**	yellow	colorless	colorless
**Crystal system**	triclinic	orthorhombic	monoclinic
**Space group**	P-1	Pbca	$P2_1/n$
**a in Å**	10.9650(7)	8.0671(2)	12.9559(7)
**b in Å**	11.6978(7)	15.0932(4)	10.4977(6)
**c in Å**	13.6443(9)	22.4852(6)	28.982(2)
**α in °**	111.740(3)	90	90
**β in °**	101.903(3)	90	95.735(2)
**γ in °**	90.098(3)	90	90
**V in Å^3**	1584.8(2)	2737.76(12)	3922.0(4)
**Z**	1	8	8
**T in K**	100(2)	233(2)	85(2)
**Crystal size in mm^3**	0.13 × 0.10 × 0.08	0.14 × 0.08 × 0.02	0.155 × 0.140 × 0.035
**ρ_c in g cm^{-3}**	1.785	1.157	1.241
**F(000)**	843	1040	1360
**Diffractometer**	Bruker-D8 Venture	Bruker-D8 Venture	Bruker-D8 Venture
**$\lambda_{MoK\alpha}$ in Å**	0.71073	0.71073	0.71073
**θ_{min} in °**	1.881	2.699	2.06
**θ_{max} in °**	29.998	29.999	31.08
**Index range**	$-15 \leq h \leq 15$<br>$-16 \leq k \leq 16$<br>$-19 \leq l \leq 19$	$-11 \leq h \leq 10$<br>$-21 \leq k \leq 21$<br>$-31 \leq l \leq 31$	$-13 \leq h \leq 18$<br>$-15 \leq k \leq 15$<br>$-42 \leq l \leq 42$
**μ in mm^{-1}**	4.819	0.157	0.187
**Abs. correction**	multi-Scan	multi-Scan	multi-Scan
**Reflections collected**	71687	47682	112870
**Reflections unique**	9245	3992	12536
**R_{int}**	0.0825	0.0722	0.0420
**Reflections obs. [F>2σ(F)]**	7237	2998	10539
**Residual density in e Å^{-3}**	2.340/-0.680	0.357/-0.199	0.943/-0.357
**Params/restraints**	355/2	151/0	391/0
**GOOF**	1.092	1.082	1.054
**R_1 [I>2σ(I)]**	0.0477	0.0444	0.0530
**wR_2 (all data)**	0.1428	0.1205	0.1426
**CCDC**	2190208	2190209	2190210

13.1. Molecular Structure of Complex Mn-5 (CCDC 2190208)

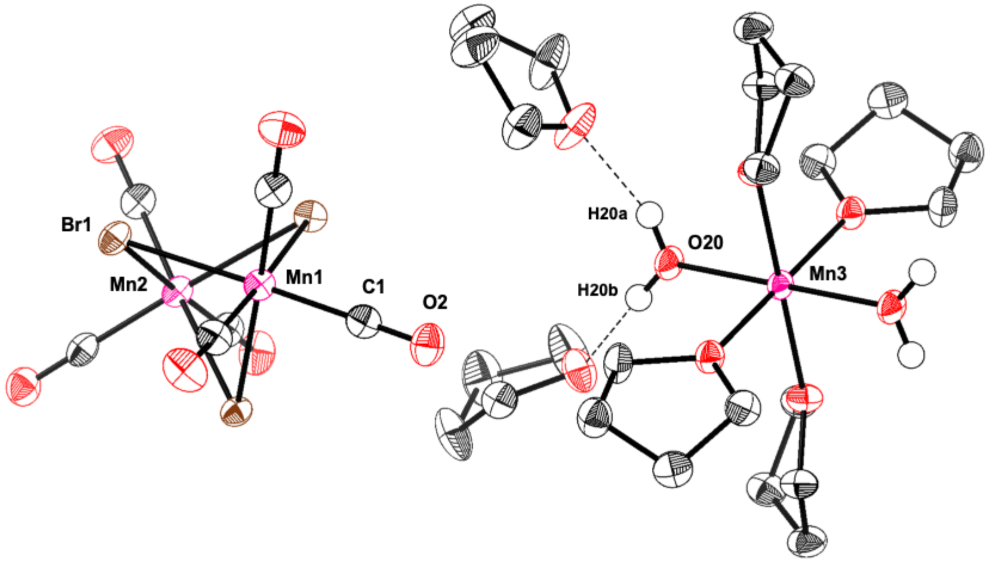

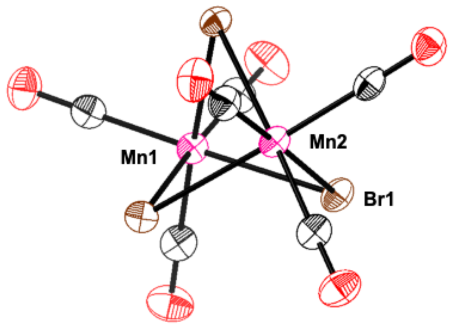

Molecular structure of complex **Mn-5**. Single crystals suitable for XRD were obtained by slow diffusion of Et_2O and pentane into a concentrated THF solution of the solid from the reaction described in Section S10.4. The molecular structure is shown with thermal ellipsoids drawn at the 40% probability level. For clarity reasons, most of the hydrogen atoms were omitted.

13.2. Molecular Structure of Silanol 2p (CCDC 2190209)

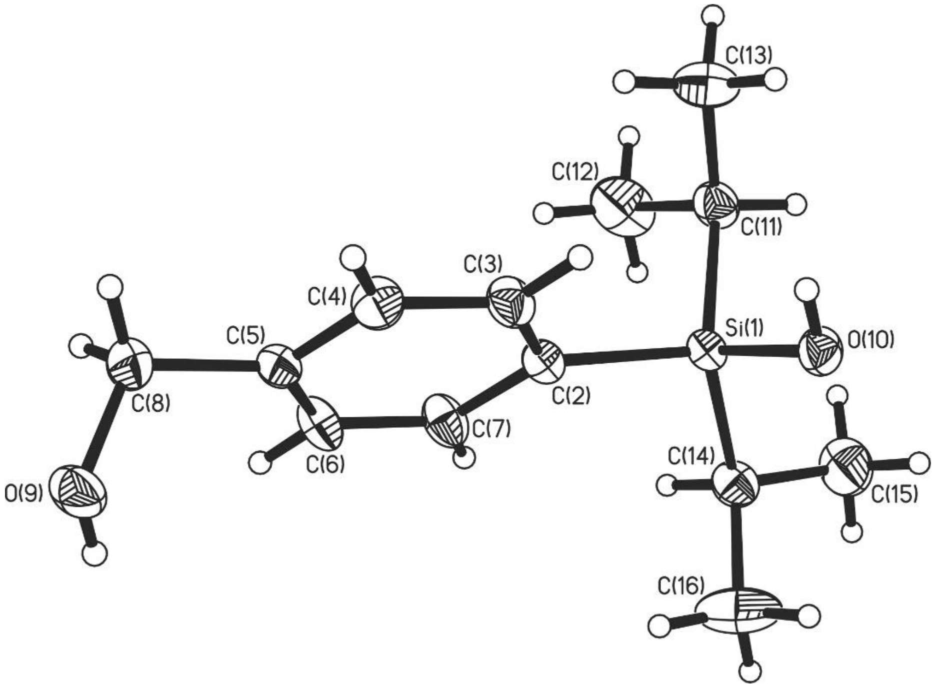

Molecular structure of the silanol **2p**. Single crystals suitable for XRD analysis were obtained by slowly cooling a $CHCl_3$ solution of **2p** from RT to -20 °C.

13.3. Molecular Structure of Silanol 2o (CCDC 2190210)

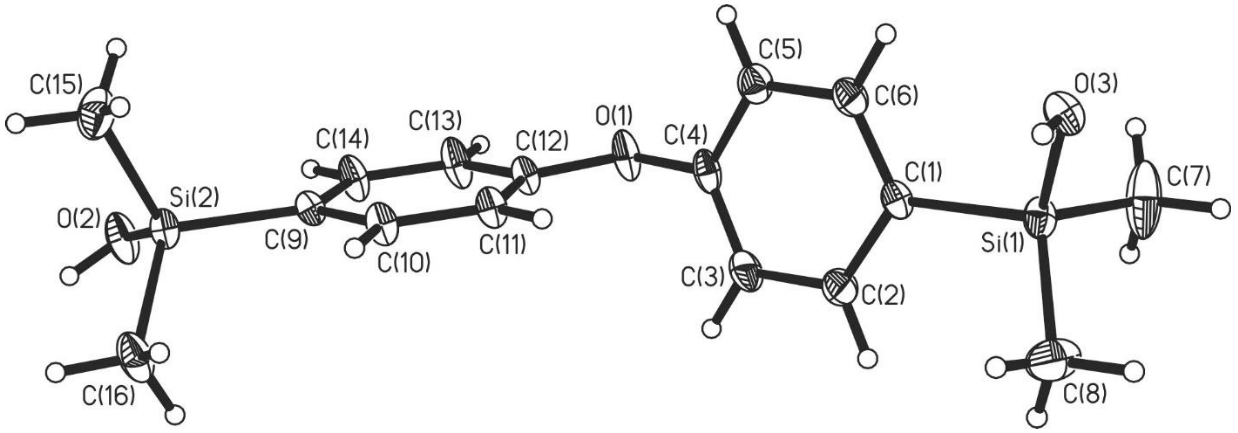

Single crystals suitable for XRD analysis were obtained by slowly cooling a $CHCl_3$ solution of **2o** from RT to -20 °C.

14. References

[1] A. Bruneau-Voisine, D. Wang, T. Roisnel, C. Darcel, J.-B. Sortais, *Catal. Commun.* **2017**, *92*, 1-4.

[2] A. Kaithal, M. Holscher, W. Leitner, *Angew. Chem., Int. Ed.* **2018**, *57*, 13449-13453.

[3] S. J. A. Pope, G. Reid, *J. Chem. Soc., Dalton Trans.* **1999**, 1615-1622.

[4] a) N. Gandhamsetty, S. Park, S. Chang, *J. Am. Chem. Soc.* **2015**, *137*, 15176-15184; b) Q. Dai, L. Liu, Y. Qian, W. Li, J. Zhang, *Angew. Chem., Int. Ed.* **2020**, *59*, 20645-20650.

[5] K. Wang, J. Zhou, Y. Jiang, M. Zhang, C. Wang, D. Xue, W. Tang, H. Sun, J. Xiao, C. Li, *Angew. Chem., Int. Ed.* **2019**, *58*, 6380-6384.

[6] S. E. Denmark, J. M. Kallemeyn, *Org. Lett.* **2003**, *5*, 3483-3486.

[7] H. Liang, L. J. Wang, Y. X. Ji, H. Wang, B. Zhang, *Angew. Chem., Int. Ed.* **2021**, *60*, 1839-1844.

[8] S. Bahr, S. Brinkmann-Chen, M. Garcia-Borras, J. M. Roberts, D. E. Katsoulis, K. N. Houk, F. H. Arnold, *Angew. Chem., Int. Ed.* **2020**, *59*, 15507-15511.

[9] P. D. Lickiss, R. Lucas, *J. Organomet. Chem.* **1996**, *521*, 229-234.

[10] J. F. Kannengiesser, M. Briesenick, D. Meier, V. Huch, B. Morgenstern, G. Kickelbick, *Chem. - Eur. J.* **2021**, *27*, 16461-16476.

[11] a) J. Bures, *Angew. Chem., Int. Ed.* **2016**, *55*, 2028-2031; b) J. Bures, *Angew. Chem., Int. Ed.* **2016**, *55*, 16084-16087; c) C. D. Nielsen, J. Bures, *Chem. Sci.* **2019**, *10*, 348-353.

[12] SADABS, Bruker–Siemens Area Detector Absorption and Other Correction, G.M. Sheldrick, University of Göttingen, Germany, 2012, Version 2012/1.

[13] ShelXTL MP Vers. 2013-2, Bruker AXS Inc., 1993-2013.

[14] A. L. Spek, *Acta Crystallogr. C Struct. Chem.* **2015**, *71*, 9-18.
